# Knockdown of SETD2 promotes erastin-induced ferroptosis in ccRCC

**DOI:** 10.1038/s41419-023-06057-8

**Published:** 2023-08-21

**Authors:** Wei Xue, Wengang Jian, Yuyang Meng, Tengda Wang, Licheng Cai, Yongchun Yu, Yipeng Yu, Zhinan Xia, Cheng Zhang

**Affiliations:** 1 Department of Urology, the First Affiliated Hospital of Harbin Medical University, Harbin, 150001 Heilongjiang China; 2 Department of Urology, the Fourth Affiliated Hospital of Zhejiang University School of Medicine, Yiwu, 322000 Zhejiang China

**Keywords:** Renal cell carcinoma, Tumour-suppressor proteins

## Abstract

Clear cell renal cell carcinoma (ccRCC) is the most common subtype of kidney cancer and is associated with poor prognosis. The histone H3 lysine 36 methyltransferase SET-domain-containing 2 (SETD2) has been reported to be expressed at low levels and frequently mutated in ccRCC. Ferroptosis, a form of death distinct from apoptosis and necrosis, has been reported in recent years in renal cancer. However, the relationship between SETD2 and ferroptosis in renal cancer is not clear. Here, we demonstrated that SETD2 was expressed at low levels in ccRCC and was associated with poor prognosis. Moreover, we found that knockdown of SETD2 increased lipid peroxidation and Fe^2+^ levels in tumor cells, thereby increasing the sensitivity of erastin, a ferroptosis inducer. Mechanistically, histone H3 lysine 36 trimethylation (H3K36me3) which was catalyzed by SETD2, interacted with the promoter of ferrochelatase (FECH) to regulate its transcription and ferroptosis-related signaling pathways. In conclusion, the presesnt study revealed that knockdown of the epigenetic molecule, SETD2, significantly increases the sensitivity of ferroptosis inducers which promotes tumor cell death, thereby indicating that SETD2 may be a potential therapeutic target for ccRCC.

## Introduction

Renal cell carcinoma is the most common malignant tumor of the kidney in adults and mainly arises from renal tubular epithelial cells [1]. ccRCC is the most common pathological subtype of renal cell carcinoma, accounting for approximately 75% of renal cell carcinomas [2]. Although early-stage kidney cancer can be cured by surgery, more than 30% of patients develop distant metastases before diagnosis because its early symptoms are not obvious, leading to a poorer prognosis [3, 4]. Moreover, its poor prognosis is also associated with its insensitivity to chemotherapy and lack of biomarkers [5]. Therefore, individualized treatment options are urgently needed for patients with ccRCC.

Because epigenetic alterations, such as DNA methylation, histone modifications and nucleosome remodelling all control gene expression, are involved in the development and progression of kidney cancer, epigenetically targeted therapies have the potential to become a new therapeutic prospect [6, 7]. The incidence of SETD2 inactivating mutations in cancer is highest in ccRCC [8]. SETD2 is expressed at low protein levels in kidney cancer, mainly due to chromosome 3 P deletion or translocation, which is the only known H3K36-unique trimethyltransferase [9, 10]. SETD2-mediated H3K36me3 has multiple biological functions, including transcription elongation, repression of cryptic transcription initiation, cotranscriptional RNA processing, alternative splicing, DNA mismatch repair and DNA double-strand break repair by homologous recombination [11–13]. In addition, non histone substrates of SETD2, including α-tubulin and STAT1, have been reported [14, 15]. Many studies have demonstrated the important role of SETD2 in maintaining chromatin stability and integrity [16].

FECH is a key enzyme in hemoglobin synthesis, that catalyzes the insertion of ferrous iron into photoporphyrin IX (PPIX) to form heme [17]. It has been reported that low or deficient expression levels of FECH cause the accumulation of iron ions and PPIX, which enhances ROS levels [17, 18]. In addition, FECH deficiency also affects transferrin expression and redistribution of iron in vivo [19–21]. A recent study has shown that FECH downregulation promotes ferroptosis in cancer cells [22].

Ferroptosis is a form of death that is distinct from apoptosis and necrosis and it is characterized by the accumulation of iron and lipid peroxidation resulting in the production of ROS and subsequent cell death [23, 24]. Importantly, ferroptosis has been reported in a variety of diseases, such as neuropsychiatric diseases, kidney degeneration, and ischemia‒reperfusion injury [25]. A recent study has also indicated that dysfunction of ferroptosis is closely related to tumor progression [26]. A previous study has investigated the impact of KEAP1-NRF2 pathway on ferroptosis in lung cancer [27], and lactate upregulates SCD1, which increases the resistance to ferroptosis in liver cancer [28]. BAP1 inhibits SLC7A11 expression in a deubiquitination-dependent manner, leading to increased ROS levels and ferroptosis [29]. CREB stimulates GPX4 transcription to inhibit ferroptosis in lung adenocarcinoma [30]. However, studies concerning ferroptosis in ccRCC are insufficient. Therefore, ferroptosis may also be relevant as a therapeutic target for renal cancer.

In the present study, we examined whether SETD2 is expressed at low levels in ccRCC and low SETD2 expression is associated with poor prognosis. Furthermore, we knocked down SETD2 and found that increased sensitivity to erastin promoted ferroptosis in ccRCC. Mechanistically, SETD2-mediated downregulation of H3K36me3 led to reduced expression of FECH at the transcriptional level, which increased ROS levels and the accumulation of iron ions. Theses findings may provide a new proof-of-concept that targeting an epigenetic factor for the comprehensive treatment of ccRCC.

## Materials and methods

### Cell lines, cell culture and tissue specimens

The human renal cancer cell lines (786-O, ACHN OS-RC-2, and A498) were all purchased from the Cell Resources Center, Shanghai Academy of Life Sciences, Chinese Academy of Sciences. All cell lines were incubated in the recommended medium supplemented with 10% heat-inactivated fetal bovine serum (Gibco, Beijing, China) and 1% streptomycin/penicillin (Keygen, Nanjing, China) at 37 °C and 5% CO_2_. All kidney cancer tissue samples were obtained from 40 patients who were treated by radical nephrectomy or preserved kidney surgery at the Department of Urology, First Affiliated Hospital of Harbin Medical University between 2010 and 2019. All diagnoses were confirmed by histopathological examination.

### Antibodies and reagents

The following antibodies were used for Western blot analysis: SETD2 (55377-1-AP, Proteintech), FECH (14466-1-AP, Proteintech), H3K36me3 (4909T, CST), H3 (ab177253, Abcam), transferrin (17435-1-AP, Proteintech), transferrin receptor protein 1 (10084-2-AP, Proteintech), GPX4 (67763-1-Ig,Proteintech), and GAPDH (60004-1-ig, Proteintech). The anti-SETD2 antibody (55377-1-AP) and anti-FECH antibody (14466-1-AP) for IHC were purchased from Proteintech. The anti-H3K36me3 antibody for ChIP-PCR was purchased from Abcam (61101). Erastin (HY-15763) and ferrostatin-1 (HY-100579) was purchased from MCE.

### siRNA and plasmid transfection

The siRNAs for SETD2 and FECH were purchased from GenePharma (Suzhou, China), and their sequences are shown in Supplementary Table S1. Transfection of the siRNA-SETD2 and siRNA-FECH vectors was performed using jetPRIME (PolyPlus, Shanghai, China). The plasmids (pENTER-FECH, pENTER-NC, pCMV3-FECH and pCMV3-NC) were purchased from Weizhen (Shandong, China) and SinoBiological (Beijing, China). The pCMV3-SETD2 and pCMV3-NC pcDNA3.1-3xFlag-C plasmids were purchased from Weizhen (Shandong, China) and SinoBiological (Beijing, China). Briefly, 1.2–1.3 × 10^6^ cells were seeded into a 6 cm culture dish to prepare for transfection, and ccells were collected for subsequent experiments after 47–72 h.

### Lentiviral vector construction and transfection

In brief, a short hairpin RNA (shRNA) homologous to SETD2 or a control shRNA was integrated into the pHBLV-U6 backbone. The shRNA sequences are listed in Supplementary Table S1. Cells were infected and subjected to puromycin selection before the experiments were performed.

### Quantitative real-time PCR (qRT‒PCR)

Total RNA from cells and tissues was extracted utilizing TRIzol reagent (Ambion, USA). Reverse transcription kits (TianGen, Beijing, China) and PCR kits (SYBR Green) (TianGen, Beijing, China) were used to perform qRT-PCR according to the manufacturer’s instructions. The primer sequences of primers are provided in Supplementary Table S1. The results were analyzed by the 2^−ΔΔCt^ method to quantify fold changes.

### Western blot analysis and immunohistochemistry (IHC)

Proteins from nude mouse renal cancer cells and tissues as well as tumor cell lines were extracted with RIPA buffer (Beyotime, Shanghai, China) supplemented with protease inhibitors. Equal amounts of proteins were separated by SDS‒PAGE and transferred to PVDF membranes. Membranes were blocked with 5% BSA for 2 h at room temperature and then incubated with the corresponding primary antibodies. For IHC, paraffin-embedded 4-μm-thick kidney cancer tissue sections were deparaffinized, subjected to antigen retrieval, blocked with 5% bovine serum albumin (BSA) for 2 h at room temperature, and incubated overnight with antibodies against SETD (1:200) and FECH (1:400) at 4 °C. Peroxidase-conjugated polymer was then added followed by incubation for 30 min. and DAB was then used for visualization (Beyotime, Shanghai, China).

### CCK-8 cell proliferation assay

The CCK-8 assay (MCE, China) was used to analyze cell activity. Transfected cells were cultured in 96-well plates (2000 cells/well) for 12 h, and 10% CCK8 solution was added at different time periods (0, 24, 48, and 72 h) according to the manufacturer’s protocol. The OD absorbance values were measured at 450 nm using a 96-well plate reader.

### Lipid ROS assay

Lipid ROS in tumor cells were detected with C11-BODIPY (C10445, Invitrogen) according to the manufacturer’s instruction. Briefly, treated tumor cells were incubated with 10 mmol/L C11-BODIPY in the dark at, 37 °C and 5% CO_2_, for 30 min. After washing three times with cold PBS, the C11-BODIPY green/red fluorescence (510 nm and 590 nm) was measured with a flow cytometer and fluorescence microscope.

### Iron assay

An Iron Assay Kit (ab83366, Abcam) was used to measure the Fe^2+^ content of cells, according to the manufacturer’s instruction. In brief, treated tumor cells were homogenized in 100 µl of iron assay buffer and centrifuged at 16,000 × *g* for 10 min at 4 °C. A standard iron mixture was prepared with 10 µl of standard iron and 990 µl of dd water, and this mixture was added to the experimental and standard wells in a 96-well plate to a final volume of 100 µl. Then, 5 µl of iron reducing agent was added to each well and incubated for 30 min at 37 °C. Then, 100 µl of iron probe was added followed by incubation for 60 min at 37 °C in the dark. Finally, the absorbance was measured at 593 nm (A593).

### MDA assay

An MDA Assay Kit (ab118970, Abcam) was used to measure the MDA content of cells according to the manufacturer’s instructions. Standards were prepared with 407 µl of dd water and 10 µl of standard solution to form 0.1 M MDA, and the treated tumor cells (2 × 10^6^) were washed three times with cold PBS before lysis with MDA lysis solution containing BHT. The above mixture was processed by a Donce homogenizer and then centrifuged at 16,000 × *g* at 4 °C for 10 min. Then, 200 µl of the standard group and 200 µl of the experimental group were added to 600 µl of TBA followed by incubation at 95 °C for 10 min. After cooling the samples to room temperature in an ice bath, the absorbance was measured at 532 nm (A532).

### Chromatin immunoprecipitation assay (ChIP)

The chromatin immunoprecipitation assay with ACHN and OS-RC-2 cells was performed using the SimpleChIP® Plus Enzymatic Chromatin IP Kit (Magnetic Beads) according to the manufacturer’s instructions. First, 1 × 10^7^ cells were immobilized with 1% formaldehyde, and 1.25 M glycine was added to terminate the reaction. Cells were washed with cold PBS containing protease inhibitor and collected into a centrifuge tube. Cells were then sonicated in IP mixed medium and centrifuged at 14,000 × *g* for 30 min at 4 °C. The above supernatant was centrifuged and mixed with protein A/G agarose beads, and the chromatin in the supernatant was immunoprecipitated overnight with antibodies against H3K36me3 or IgG. The protein A/G agarose beads were then washed with wash buffer containing LiCl/detergent and TE buffer. The beads were heated overnight at 65 °C in a buffer containing 1% SDS and 0.1 M NaHCO_3_ to crosslink the beads in reverse. The beads were then treated with 1 ml of RNaseA for 15 min at 37 °C and digested with 2 ml of proteinase K solution for 1 h at 37 °C. Finally, DNA was purified by extraction with LiCl, phenol/chloroform, and ethanol, and the specific FECH promoter region was amplified with a DNA template. Supplementary Table S1 lists the primer sequences designed to detect specific promoters.

### Xenograft tumor growth

We randomized 5-week-old male nude mice to group them according to experimental grouping needs. They were bred for a week so they can adapt to their environment. A total of 3 × 10^7^ stable SETD2-knockdown (shSETD2) or control (shCtrl) ACHN cells were injected into the posterior ventral sides of 6-week-old male nude mice purchased from Charles River Laboratories (Beijing, China). After 8 days, tumors were visible, their volumes were measured. Erastin was intratumorally injected into the mice at a dose of 5 mg/kg once every three days. After 42 days, the mice were sacrificed, and the tumors were removed. The tumor weights and volumes were measured. The animal protocol complied with the regulations of the Animal Ethics Committee of Harbin Medical University.

### TCGA data

RNA-sequencing expression (level 3) profiles and corresponding clinical information for KIRC were downloaded from the TCGA dataset (TCGA, https://portal.gdc.cancer.gov/). Statistical analyses were performed using R software v4.0.3 (R Foundation for Statistical Computing, Vienna, Austria). One-way analysis of variance (ANOVA) was conducted, and genes were considered differentially expressed using a log_2_ fold change cut-off value 1 and p value cut-off 0.01.

### Statistical analysis

Statistical analyses were performed using GraphPad software (version: 7.0). Data are expressed as the mean ± standard deviation (SD). The statistical difference of two groups was compared through the *t*-test, significant difference among three groups or more was tested with Kruskal–Wallis test. *P* < 0.05, **P < 0.01, ***P < 0.001.

## Results

### SETD2 expression is downregulated in ccRCC tumors and correlates with progression and prognosis

**Fig. 1:**
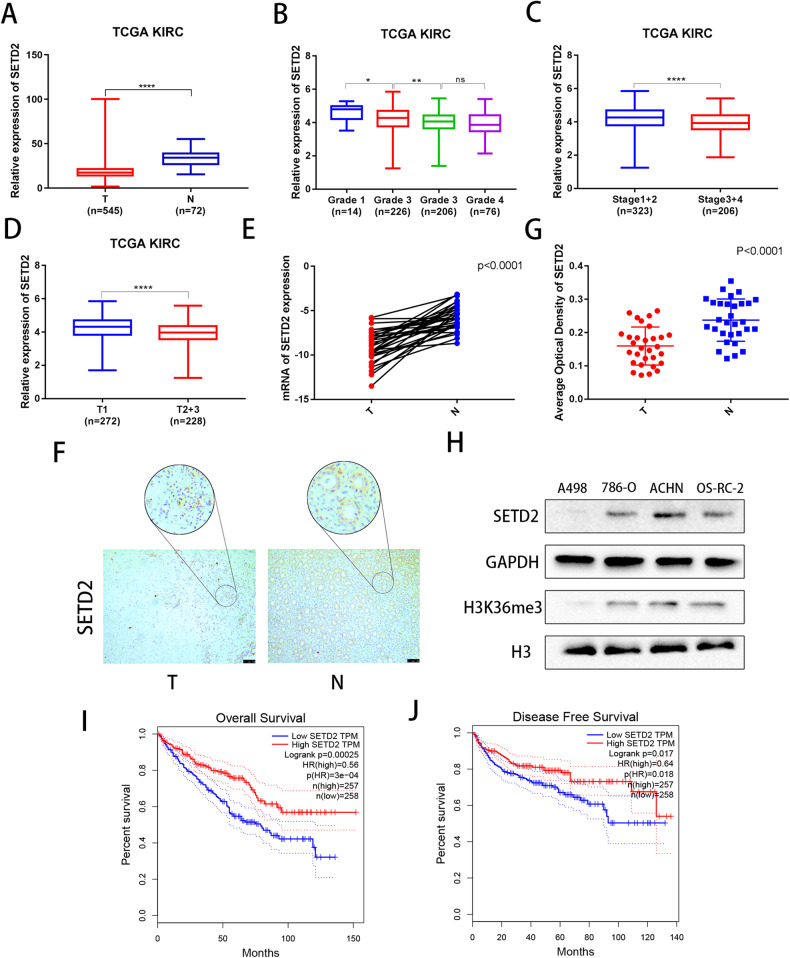
SETD2 expression is downregulated in renal tumors and correlates with progression. **A** The mRNA expression of SETD2 in normal tissues and ccRCC tissues from TCGA database. **B**–**D** Correlation analysis of SETD2 mRNA expression and histological grade, clinical TNM stage, as well as depth of invasion n (T stage). **E** SETD2 mRNA expression in 32 pairs of ccRCC samples. Mean ± SD is shown. Statistical analysis was conducted using a *t* test (*p < 0.05) **F**, **G** Immunohistochemical verification of SETD2 protein levels in 30 pairs of cancer and adjacent nontumor tissues. **H** Western blot verification of SETD2 and H3K36me3 protein levels in A498, 786-O, ACHN and OS-RC-2 cell lines. **I**, **J** Overall survival (OS) Kaplan–Meier curve and disease free survival DFS Kaplan–Meier curve for SETD2 based on TCGA. *P < 0.05, **P < 0.01, ***P < 0.001.

It has been shown that the epigenetic molecule, SETD2, plays a key role in the progression of various tumors [31]. To assess the clinical role of SETD2 in ccRCC progression, we first investigated the expression pattern of SETD2 in ccRCC using TCGA database. SETD2 was significantly differentially underexpressed in tumor tissues compared to normal tissues (Fig. 1A). SETD2 expression was also correlated with many features of tumor progression, including histological grade, clinical TNM stage, and invasion depth (T stage), which implied that low SETD2 expression was associated with high histological grade, clinical TNM stage, and T stage (Fig. 1B–D). To further investigate SETD2 expression in ccRCC, an qRT‒PCR experiment was performed with paired normal and ccRCC tissue samples from patients (Fig. 1E), and the results were consistent with those of TCGA database. Moreover, we verified the differential expression of SETD2 in normal and ccRCC tissues from patients using immunohistochemistry (Fig. 1F, G). Subsequently, we examined the expression levels of SETD2 protein in several renal cancer cell lines (A498, ACHN, 786-O, and OS-RC-2), and its expression was consistent with H3K36me3 (Fig. 1H). It should be noted that all four of these cell lines were 3p-deleted [32], and the remaining copy of SETD2 was only mutated in A498. Finally, we created a survival curve of the predicted SETD2 using The Cancer Genome Atlas (TCGA) database, and survival analysis indicated that a low level of SETD2 expression was significantly correlated with shorter OS and DFS in ccRCC patients (Fig. 1I, J). Taken together, these results suggested that SETD2 is expressed at low levels in ccRCC and low SETD2 expression is associated with poor prognosis.

### SETD2 knockdown promotes lipid peroxidation, promotes Fe^2+^ accumulation, and increases erastin sensitivity in ccRCC

**Fig. 2:**
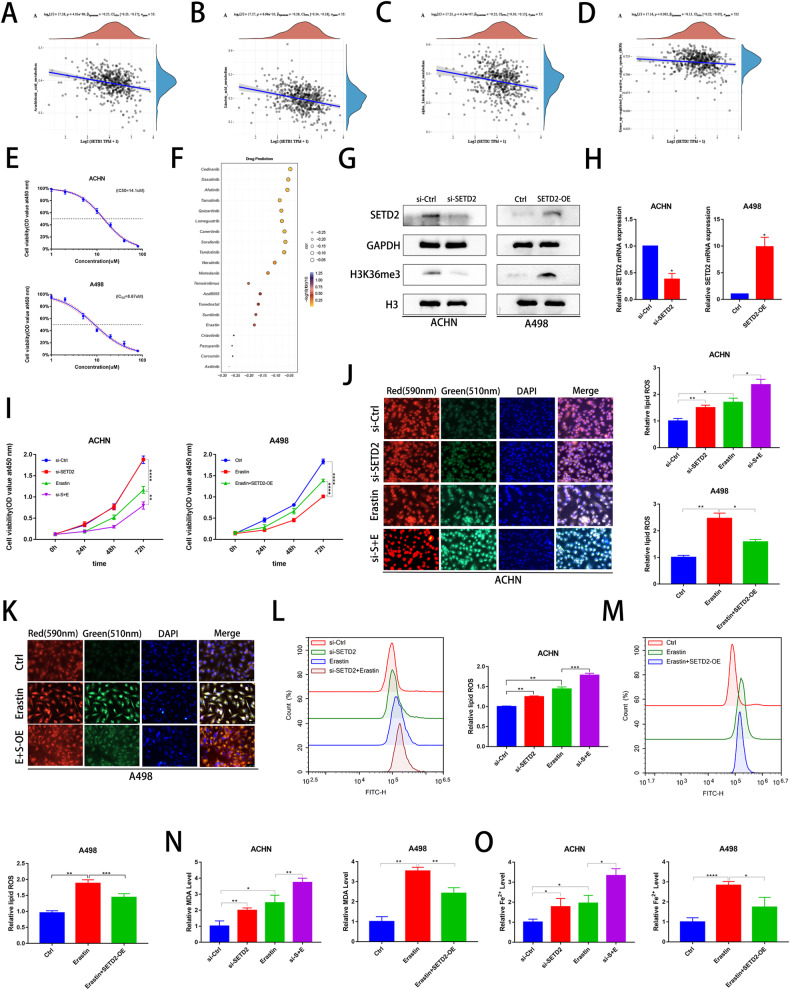
SETD2 knockdown promotes ROS levels, promotes Fe^2+^ accumulation, and increases erastin sensitivity. **A**–**D** Correlation analysis of SETD2 mRNA expression and arachidonic acid, linoleic acid, α-linolenic acid as well as ROS. **E** The IC50 of erastin was detected in the ACHN and A498 cell lines. **F** The sensitivity of SETD2 to various drugs from the CTRP database. **G**, **H** Western blot and qRT‒PCR examined the efficiency of SETD2 knockdown and overexpression in ACHN and A498 cell lines, respectively. **I** CCK-8 assays were used to analyse the effect in the si-Ctrl, si-SETD2, erastin as well as si-S + E groups on cell viability in ACHN and the effect in the Ctrl, erastin as well as erastin+SETD2-OE groups on cell viability in A498. **J**–**M** Immunofluorescence (the green to red ratio represents lipid peroxidation levels) and flow cytometry was used to detect lipid peroxidation in the si-Ctrl, si-SETD2, erastin as well as si-S + E groups in ACHN and in the Ctrl, erastin as well as erastin+SETD2-OE groups in A498. **N**, **O** Levels of MDA and Fe^2+^ were analyzed in the si-Ctrl, si-SETD2, erastin as well as si-S + E groups in ACHN and in the Ctrl, erastin as well as erastin+SETD2-OE groups in A498. *P < 0.05, **P < 0.01, ***P < 0.001.

To further explore the function of SETD2 in ccRCC, we performed GO analysis using data from TCGA database, and we found that low SETD2 expression was more effective in promoting lipid-related metabolism (Supplementary Fig. S2A). Moreover, we found that SETD2 expression was negatively correlated with many gene expression of polyunsaturated fatty acids (PUFAs; arachidonic acid, linoleic acid, and α-linolenic acid) and ROS level (Fig. 2A–D). It has been reported that SETD2 depletion results in excessive reactive oxygen species (ROS) [33]. Because lipid peroxidation is an important feature of ferroptosis, we investigated whether knockdown of SETD2 promotes lipid peroxidation. We treated ACHN and A498 (SETD2^−/−^) cells with a concentration gradient of erastin (ferroptosis inducer), and the half inhibitory concentrations (IC50) were 14.1 µM and 8.87 µM, respectively (Fig. 2E), which implied that SETD2 may affect the sensitivity of erastin. We used the Cancer Therapeutics Response Portal (CTRP) database and found that SETD2 was associated with many targeted drugs, such as sorafenib, axitinib, sunitinib, and erastin (Fig. 2F). Subsequently, the efficiency of SETD2 overexpression or knockdown was verified using western blot and qRT‒PCR analyses in A498 and ACHN cells (Fig. 2G, H). We performed a CCK8 assay and found that knockdown of SETD2 increased erastin sensitivity in ACHN cells, conversely, restoration of SETD2 expression also inhibited erastin sensitivity in A498 cells (Fig. 2I). We also overexpressed SETD2 in ACHN cell lines and found that overexpression of SETD2 decreased the sensitivity of tumor cells to erastin (Supplementary Fig. S2B). We then measured intracellular lipid ROS levels by IF and FCM in the presence of knockdown or overexpression of SETD2. As expected, knockdown of SETD2 increased the induction of erastin on ROS in ACHN and A498 cell lines (Fig. 2J–M). Finally, we demonstrated that knockdown of SETD2 also increased the erastin effects on Fe^2+^ and MDA, whereas overexpression of SETD2 decreased its effects (Fig. 2N, O). Together, these results demonstrated that SETD2 knockdown promotes erastin-induced accumulation of lipid ROS and Fe^2+^ in ccRCC.

### Ferrostatin-1 reverses the ability of SETD2 knockdown to increase sensitivity to erastin-induced ferroptosis

**Fig. 3:**
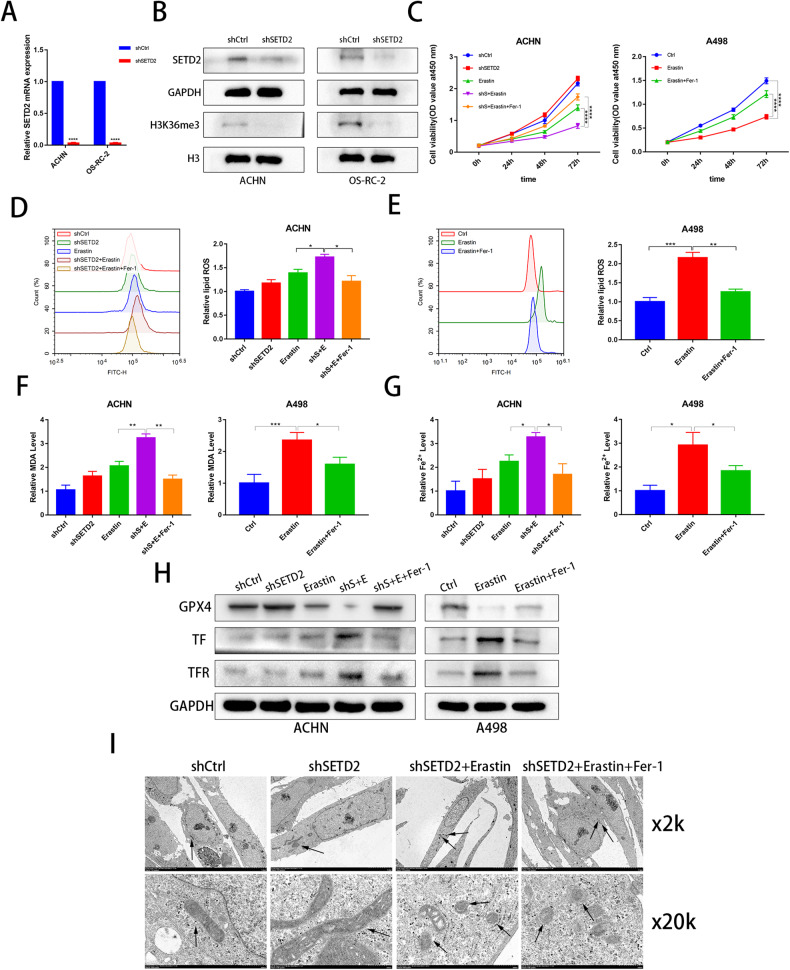
Ferrostatin-1 reverses the ability of SETD2 knockdown to increase sensitivity to erastin-induced ferroptosis. **A**, **B** qRT‒PCR and western blot examined the efficiency of SETD2 knockdown transfected with the SETD2-specific shRNA. **C** CCK-8 assays were used to analyse the effect in the shCtrl, shSETD2, erastin, shS+E as well as shS+E+Fer-1 groups on cell viability in ACHN and the effect in the Ctrl, erastin as well as erastin+Fer-1 groups on cell viability in A498. **D**, **E** Flow cytometry was used to detect lipid peroxidation in the shCtrl, shSETD2, erastin, shS+E as well as shS+E+Fer-1 groups in ACHN and in the Ctrl, erastin as well as erastin+Fer-1 groups in A498. **F**, **G** Levels of MDA and Fe^2+^ were analyzed in the shCtrl, shSETD2, erastin, shS+E as well as shS+E+Fer-1 groups in ACHN and in the Ctrl, erastin as well as erastin+Fer-1 groups in A498. **H** GPX4, TF and TFR protein was detected in the shCtrl, shSETD2, erastin, shS+E as well as shS+E+Fer-1 groups in ACHN and in the Ctrl, erastin as well as erastin+Fer-1 groups in A498. **I** Electron microscopy imaging was conducted in the shCtrl, shSETD2, shS+E as well as shS+E+Fer-1 groups in ACHN, Black arrows marked mitochondrial changes. *P < 0.05, **P < 0.01, ***P < 0.001.

Lipid peroxidation and Fe^2+^ alterations are the most important hallmarks of ferroptosis. Knockdown of SETD2 alone did not significantly alter tumor cell activity (Fig. 2I). Thus, knockdown of SETD2 enhanced erastin-induced tumor cell death via ferroptosis. Furthermore, we established stable SETD2 knockdown cell lines through lentivirus transduction, and the knockdown efficiency was verified using qRT‒PCR analysis or WB analysis for SETD2 (Fig. 3A, B). Fer-1 (a ferroptosis inhibitor) reversed shSETD2-enhanced erastin-induced ferroptosis (Fig. 3C). Similarly, ROS, MDA, and Fe^2+^ levels were reversed by fer-1 when shSETD2 was combined with erastin (Fig. 3D–G). Erastin combined with shSETD2 induced ferroptosis inhibited GPX4 expression and increased TF and TFR expression to a greater extent than either treatment alone (Fig. 3H). Interestingly, shSETD2 alone was able to increase GPX4 protein expression, which is possible that it was compensatorily to balance the redox homeostasis of the cancer cells. We next used electron microscopy imaging in ACHN cells to demonstrate that shSETD2 significantly enhanced erastin-induced ferroptosis-specific changes in mitochondria, such as the disappearance of the mitochondrial crest and increased density of the mitochondrial membrane, and these changes were reversed by fer-1 (Fig. 3I). Taken together, these results indicated that shSETD2 increases sensitivity to erastin-induced ferroptosis and that these changes are reversed by fer-1.

### Knockdown of SETD2 increases erastin-induced ferroptosis sensitivity mainly through the H3K36me3/FECH pathway in ccRCC

**Fig. 4:**
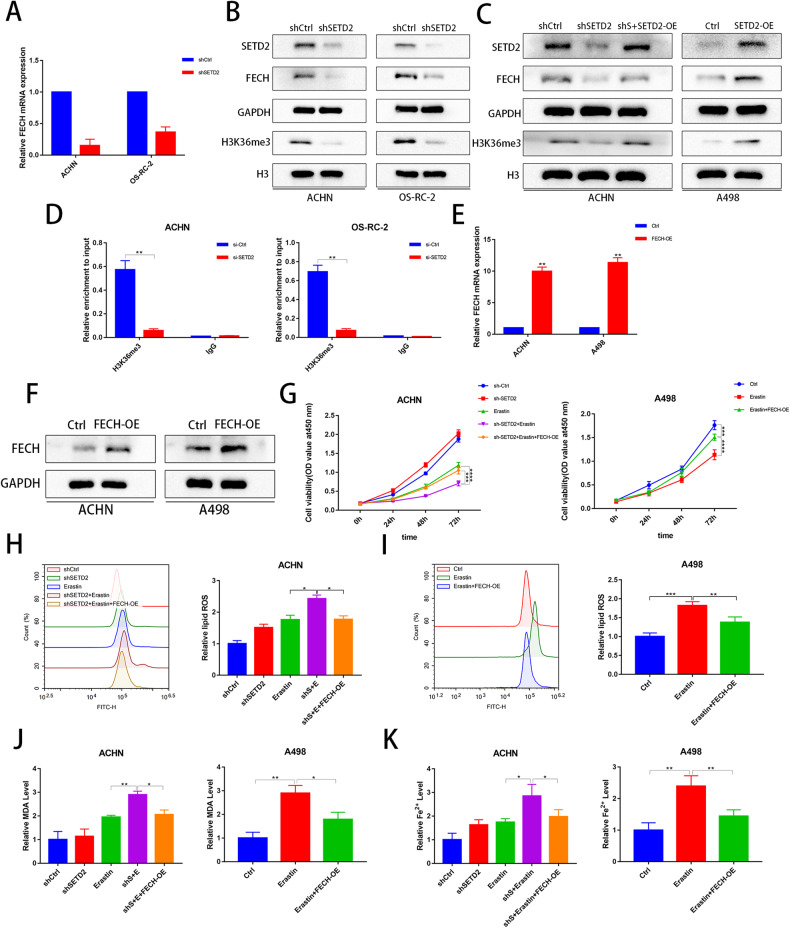
Knockdown of SETD2 increases erastin-induced ferroptosis sensitivity mainly through the H3K36me3/FECH pathway in ccRCC. **A**, **B** qRT-PCR and western blot to analyze the change of FECH and H3K36me3 caused by shSETD2. **C** Western blot to analyze the change of FECH caused by SETD2 overexpression. **D** ChIP-PCR analysis of the binding of H3K36me3 to the promoter of FECH in ACHN and OS-RC-2 cell lines. **E**, **F** qRT‒PCR and western blot examined the efficiency of FECH overexpression transfected with plasmids. **G** CCK-8 assays were used to analyse the effect in the shCtrl, shSETD2, erastin, shS+E as well as shS+E + FECH-OE groups on cell viability in ACHN and the effect in the Ctrl, erastin as well as erastin+FECH-OE groups on cell viability in A498. **H**, **I** Flow cytometry was used to detect lipid peroxidation in the shCtrl, shSETD2, erastin, shS+E as well as shS+E + FECH-OE groups in ACHN and in the Ctrl, erastin as well as erastin+FECH-OE groups in A498. **J**, **K** Levels of MDA and Fe^2+^ were analyzed in the shCtrl, shSETD2, erastin, shS+E as well as shS+E + FECH-OE groups in ACHN and in the Ctrl, erastin as well as erastin+FECH-OE groups in A498. *P < 0.05, **P < 0.01, ***P < 0.001.

As a trimethylase of H3K36, SETD2-mediated H3K36me3 promotes transcriptional activation of many genes. We analyzed H3K36me3 ChIP-seq and RNA-seq of SETD2 by taking the intersection of the gene set from an oncology-related article [34], which is listed **in** Supplementary Table S2. Interestingly, we identified two key genes for heme synthesis and catabolism, namely, FECH and HMOX1, which may be potentially associated with ferroptosis. The mRNA levels FECH were significantly decreased with shSETD2 (Fig. 4A), but the mRNA levels of HMOX1 were not affected by shSETD2 (Supplementary Fig. S4A). These findings suggested that SETD2, but not HMOX1, regulates FECH through H3K36me3 in ccRCC. Western blot analysis demonstrated that shSETD2 reduced FECH expression in ACHN and OS-RC-2 cells (Fig. 4B), and FECH expression was restored after overexpressing SETD2 in ACHN and A498 cells (Fig. 4C). Next, analysis of the ChIP-seq database (ENCODE) showed that H3K36me3 was enriched at the FECH promoter region in a variety of tumor cell lines (Supplementary Fig. S4B). To determine the ability of H3K36me3 to bind to the FECH promoter region, chromatin immunoprecipitation (ChIP) assays were performed, which showed that shSETD2 reduced binding to the FECH promoter (Fig. 4D). Subsequently, to investigate whether SETD2 promotes erastin sensitivity through FECH, we constructed an FECH overexpression plasmid, and the overexpression efficiency was verified by qRT-PCR and western blot analyses (Fig. 4E, F). Overexpression of FECH partially restored cellular activity, while shSETD2 combined with erastin promoted ferroptosis (Fig. 4G). Similarly, the levels of ROS, MDA and Fe^2+^ were reversed by overexpression of FECH, while shSETD2 combined with erastin promoted ferroptosis (Fig. 4H–K). Taken together, these results indicated that shSETD2 increases the sensitivity of erastin to promote ferroptosis mainly through the H3K36me3/FECH pathway.

### Knockdown of SETD2 enhances erastin-induced ferroptosis in a ccRCC xenograft mouse model

**Fig. 5:**
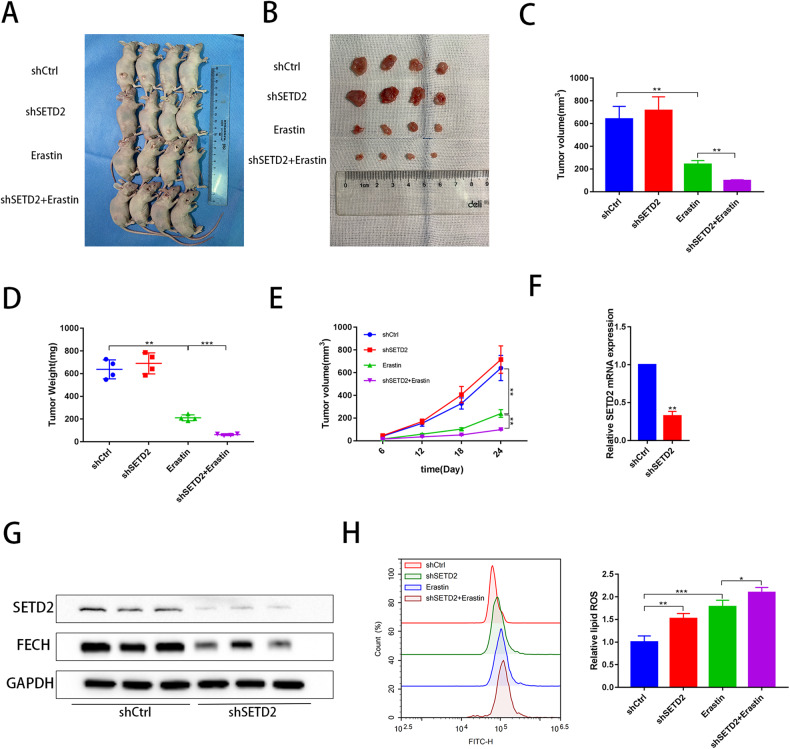
Knockdown of SETD2 enhances erastin-induced ferroptosis in vivo. **A**, **B** The subcutaneous tumors model and the separated tumors are displayed. **C**, **D** The volume and weight of tumor were measured in nude mice. **E** The tumor volume was measured at 6, 12, 18 and 24 days after tumors were formed. **F** The mRNA of FECH was measured by qRT‒PCR in the shCtrl and shSETD2 groups in vivo. **G** The protein expression levels of SETD2 and FECH were detected in the shCtrl and shSETD2 groups in vivo. **H** Flow cytometry was used to detect lipid peroxidation in the shCtrl, shSETD2, erastin, shS+E in vivo. Statistical analyses were compared using *t*-tset. *P < 0.05, **P < 0.01, ***P < 0.001.

The effect of SETD2 on erastin in ccRCC development was further demonstrated in vivo based on a xenograft mouse model. ACHN cells transfected with shCtrl or shSETD2 were subcutaneously injected into the flanks of nude mice (Fig. 5A, B). When the tumors were visible, some of the nude mice were treated with erastin. The tumors were measured once every three days. Mice were then sacrificed, and the tumor weight and volume were measured. Compared to the erastin group, the volume and weight of tumors were significantly reduced in the shSETD2 and erastin groups (Fig. 5C–E). Notably, the regulation of FECH expression by SETD2 was confirmed in vivo. Consistent with the effect in vitro, qRT‒PCR and Western blot analyses confirmed that the expression of FECH was significantly decreased by shSETD2 (Fig. 5F, G). shSETD2 also increased erastin-induced ROS levels in vivo (Fig. 5H). Therefore, these in vivo experiments demonstrated that shSETD2 combined with erastin significantly inhibits tumors.

### Knockdown of FECH increases lipid ROS and Fe^2+^ accumulation

**Fig. 6:**
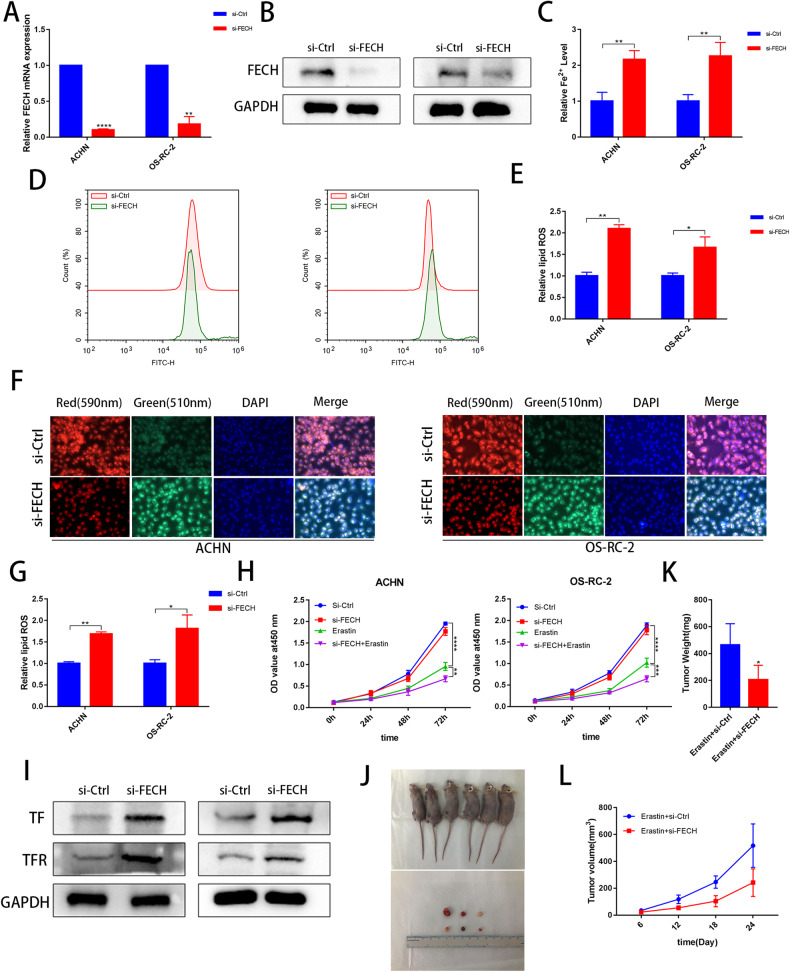
Knockdown of FECH increases lipid ROS and Fe^2+^ accumulation. **A**, **B** qRT‒PCR and western blot examined the efficiency of FECH knockdown transfected with the si-FECH. **C** Levels of Fe^2+^ were analyzed in the si-Ctrl and si-FECH groups in ACHN and OS-RC-2 cell lines. **D**–**G** Immunofluorescence (the green to red ratio represents lipid peroxidation levels) and flow cytometry was used to detect lipid peroxidation in the si-Ctrl and si-FECH groups in ACHN and OS-RC-2 cell lines. **H** CCK-8 assays were used to analyse the effect in the si-Ctrl and si-FECH groups on cell viability in ACHN and OS-RC-2 cell lines. **I** Western blot to analyze the protein change of TF and TFR caused by si-FECH. **J** The subcutaneous tumors in the 6 nude mice and the separated tumors are displayed. **K**, **L** The tumor volume was measured at 6, 12, 18 and 24 days after tumors were formed. *P < 0.05, **P < 0.01, ***P < 0.001.

FECH synthesizes protoporphyrin IX and Fe^2+^ into heme, and it is the key enzyme for heme synthesis. Therefore, we investigated whether si-FECH promotes the accumulation of protoporphyrin IX and Fe^2+^. We verified the effectiveness of the FECH siRNA knockdown strategy by qRT-PCR and WB analyses (Fig. 6A, B). As expected, si-FECH increased lipid ROS levels and Fe^2+^ accumulation (Fig. 6C–G). Similar to SETD2 knockdown, FECH knockdown did not have a significant effect on cell activity, however, siFECH can increase the sensitivity of erastin to tumor cells (Fig. 6H). Subsequently, we performed WB analysis to verify the increased TF and TFR protein expression after si-FECH transfection (Fig. 6I). In addition, we constructed a subcutaneous tumor-forming nude mouse using the OS-RC-2 cell line. We found that erastin combined with si-FECH significantly reduced the size of tumors compared to erastin treatment alone in vivo (Fig. 6J). The weight and volume of tumor in the combined si-FECH and erastin treatment group were also smaller than that in the erastin alone group (Fig. 6K, L). Thus, these findings indicated that si-FECH increases ROS levels and Fe^2+^ accumulation.

### SETD2 is correlated with FECH in ccRCC tissue samples

**Fig. 7:**
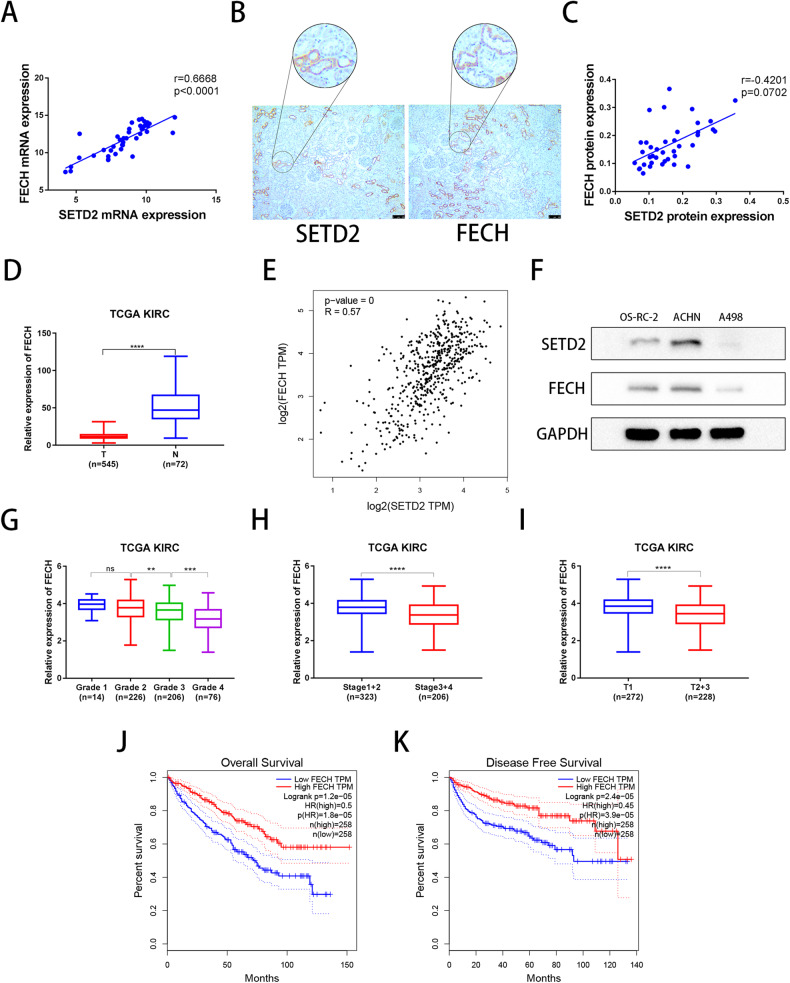
SETD2 is correlated with FECH in ccRCC tissues. **A** qRT‒PCR was used to analyse correlation of SETD2 and FECH mRNA expression in 40 pairs of ccRCC samples. **B**, **C** IHC was used to analyse correlation of SETD2 and FECH protein expression in 40 pairs of ccRCC samples. **D** The mRNA expression of FECH in normal tissues and ccRCC tissues from TCGA database. **E** Correlation of SETD2 mRNA and FECH mRNA from TCGA database. **F** Western blot verification of SETD2 and FECH protein levels in OS-RC-2, ACHN and A498 cell lines. **G**–**I** Correlation analysis of FECH mRNA expression and histological grade, clinical TNM stage, as well as depth of invasion (T stage). **J**, **K** Overall survival (OS) Kaplan–Meier curve and disease free survival DFS Kaplan–Meier curve for FECH based on TCGA. *P < 0.05, **P < 0.01, ***P < 0.001.

In the present study, we examined the relationship between SETD2 and FECH expression in tissues from ccRCC patients. qRT-PCR and IHC analyses demonstrated that the mRNA and protein levels of SETD2 and FECH were positively correlated (Fig. 7A–C), which was consistent with TCGA database (Figs. 1A, 7D, E). We then verified the expression levels of SETD2 and FECH in several ccRCC cell lines, and there was a positive correlation between SETD2 and FECH expression (Fig. 7F). FECH expression was also correlated with many features of tumor progression, including histological grade, clinical TNM stage, and invasion depth (T stage), which implied that, similar to SETD2 (Fig. 1B–D), low expression of FECH was associated with high histological grade, clinical TNM stage, and T stage (Fig. 7G–I). Further, survival analysis indicated that a low level of FECH expression was significantly correlated with shorter OS and DFS in ccRCC patients (Fig. 7J, K), which corresponded to the survival analysis of SETD2 (1I, 1J).

## Discussion

Due to the nonobvious symptoms of early-stage renal cancer, most patients are diagnosed with advanced renal cancer when surgical removal of the cancer is less curable because metastasis may have already occurred [35]. Because kidney cancer is not sensitive to radiotherapy, patients with advanced kidney cancer are usually treated with targeted drugs, but the results are not particularly satisfactory [5, 36]. Therefore, it is urgent to identify a new effective treatment for renal cancer. Histone modifications are critical in tumor initiation and development [37]. As an H3K36 methyltransferase, SETD2 has been reported to function as a tumor suppressor gene in many tumors. However, the exact role of SETD2 expression in ccRCC progression remains poorly understood. In the present study, we showed that SETD2 plays a critical role in ccRCC progression and prognosis as well as impacts tumor cell sensitivity to erastin. By analyzing data from TCGA database and our ccRCC cohorts, we found that SETD2 is expressed at low levels in ccRCC tumors and that SETD2 expression levels are significantly correlated with OS and PFS in ccRCC patients. Moreover, low expression of SETD2 is associated with a high clinical T stage. In addition, we demonstrated that knockdown of SETD2 expression increases erastin sensitivity, thereby inhibiting ccRCC cell growth in vitro and in vivo. It should be added that we chose subcutaneous tumor rather than orthotopic xenografts in terms of animal models, which has some limitations. Finally, we found that SETD2 regulates the expression of FECH at the transcriptional level mainly by mediating H3K36me3, thus affecting ferroptosis. However, overexpressing FECH with SETD2 knockdown only partially reversed ferric ion and ROS levels. These results suggested that SETD2 may affect ferric ion and ROS levels through other pathways, indicating that further studies are warranted.

Interestingly, si-SETD2 alone did not affect tumor cell activity, but it increased ROS and Fe^2+^ levels (2C-2F). These results suggested that in order to be in redox homeostasis, tumor cells compensate by producing some antioxidants and a series of antioxidant enzymes to maintain cellular homeostasis. In support of this, SETD2 knockdown alone compensatorily increased the GPX4 antioxidant enzyme (3 G), thereby explaining the sensitizing effect caused by the disruption of compensatory redox homeostasis after the addition of erastin.

FECH is most commonly associated with a disease called erythropoietic protoporphyria, which has not been thoroughly studied in tumors, especially kidney cancer [38]. FECH is a key enzyme for heme synthesis, and it is mainly found in mitochondria; the primary role of FECH is to add iron to protoporphyrin IX [17]. Therefore, when HMOX1, a key enzyme in heme catabolism, increases, it breaks down to produce large amounts of iron ions and protoporphyrin IX, thus inducing ferroptosis [39, 40]. Conversely, we hypothesized that when FECH is reduced, it also increases iron ion levels and the accumulation of protoporphyrin IX, thereby promoting ferroptosis. It has been previously reported that downregulation of FECH promotes the accumulation of ferric ions and ROS, thereby affecting the redistribution of transferrin [17–21]. It has also been recently reported that inhibition of FECH directly promotes ferroptosis in tumor cells [22]. We also verified that si-FECH increased ROS levels, Fe^2+^ levels, and transferrin expression, which may have been due to increasing the accumulation of protoporphyrin IX and thus the intracellular redox imbalance. However, the mechanism by which FECH affects ROS needs to be further investigated.

Ferroptosis is a form of death that is distinct from apoptosis and necrosis, and it may be influenced by lipid metabolism and iron metabolism [23, 24]. In recent years, studies have found that tumors and some inflammatory diseases may be associated with ferroptosis. There is increasing evidence that ferroptosis can be used in the treatment of tumors, especially those that have metastasized but are resistant to conventional therapies [41, 42]. In addition, it has been reported that ferroptosis occurs in ccRCC, which affects tumor survival and drug resistance [43, 44]. In the present study, we found that the epigenetic molecule, SETD2, increases the sensitivity of the ferroptosis inducer, erastin, further promoting ferroptosis in tumor cells. In previous studies, SETD2 has been considered a tumor suppressor, and it has a high deletion mutation rate in renal cancer. We identified links between ferroptosis and the epigenetic molecule, SETD2, which is involved in H3K36me3-mediated transcriptional regulation of FECH. We used sequencing data from other articles to screen for targets that SETD2 may regulate and affect ferroptosis through H3K36me3. We also verified that SETD2 affects the expression of FECH at the transcriptional level. We subsequently found that H3K36me3 binds to the FECH promoter region. Finally, we further determined the correlation between SETD2 and FECH at the mRNA and protein levels using clinical samples. However, the present findings did not rule out the existence of a parallel SETD2-FECH pathway affecting ferroptosis, indicating that further studies are needed.

In conclusion, we confirmed that SETD2-H3K36me3-FECH significantly potentiates the cytotoxic effect of erastin, thus providing new targets for ferroptosis intervention in ccRCC. The present study also provided mechanistic insight into the SETD2-dependent epigenetic regulation of ccRCC tumor suppression, indicating that SETD2 may serve as a therapeutic target for ccRCC treatment.

## Conclusion

**Fig. 8:**
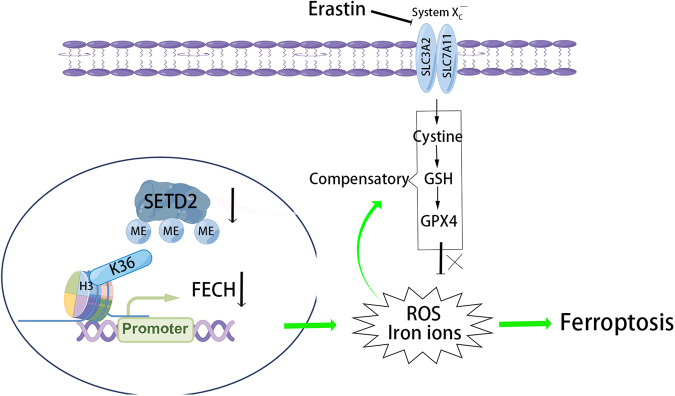
Schematic diagrams of mechanism diagram. Knockdown of the epigenetic molecule SETD2 inhibited the expression of FECH, a key enzyme for heme synthesis, via histones H3K36me3. Reduced FECH expression levels increased ROS levels and iron ion accumulation, thereby compensating for the increased antioxidant system to maintain cellular redox balance. At this time, the ferroptosis-inducer erastin can disrupt the redox balance state and promote the occurrence of ferroptosis in tumor cells, which corresponds to an increase in erastin sensitivity.

In conclusion, our study showed that knockdown of SETD2 decreased FECH expression via H3K36me3, which resulted in iron ion accumulation and elevated ROS levels. In order to maintain redox homeostasis, the antioxidant system of tumor cells is compensatingly enhanced. At the same time, we used ferroptosis-inducers erastin to inhibit the antioxidant system thereby causing ferroptosis in tumor cells, which corresponds to an increased sensitivity of the erastin (Fig. 8).

## Supplementary information

Supplementary Tabla S1

Supplementary Tabla S2

Original Data File

## Data Availability

The original data have been provided.

## Materials availability

The original materials have been provided in the Supplementary documents.
