# Supplementary material for: Knockdown of SETD2 promotes erastin-induced ferroptosis in ccRCC
Source: Cell Death Dis. 2023 Aug 21;14(8):539. doi: 10.1038/s41419-023-06057-8 (PMC10442429; doi:10.1038/s41419-023-06057-8)
Supplement: Supplementary file 1 — Supplementary Tabla S1 [file 41419_2023_6057_MOESM1_ESM.docx]

1. The sequences of siRNAs for SETD2 sequences ：

si-SETD2: (sense: GAAACCGUCUCCAGUCUGUTT, antisense: ACAGACUGGAGACGGUUUCTT)

1. The sequences of siRNAs for FECH sequences :

si-FECH: (sense: CAAGGGUAAUAAACGUGUATT, antisense: UACACGUUUAUUACCCUUGGG)

1. The control viral vector siRNA sequences and shRNA sequences are as follows:

siRNA sequences：TTCTCCGAACGTGTCACGTAA

shRNA sequences：

Top strand: GATCCGTTCTCCGAACGTGTCACGTAATTCAAGAGATTACGTGACACGTTCGGAG AATTTTTTC

Bottom strand: AATTGAAAAAATTCTCCGAACGTGTCACGTAATCTCTTGAATTACGTGACACGTTC GGAGAACG

1. The target gene siRNA sequences and shRNA sequences.

siRNA1 sequences：AGTAGTGCTTCCCGTTATAAA

shRNA1 sequences：

Top strand: GATCCGAGTAGTGCTTCCCGTTATAAACTCGAGTTTATAACGGGAAGCACTACTTTTTTTG

Bottom strand: AATTCAAAAAAAGTAGTGCTTCCCGTTATAAACTCGAGTTTATAACGGGAAGCACTACTCG

1. The primer sequences of SETD2 (Forward: CTGCCTGAATGCAAAGTTGATAG, Reverse: TGCTCTCTTTGGGCTCTATTTC)
2. The primer sequences of FECH (Forward: primers ATGGCCTAGAAAGGGCTATTG Reverse: GCTTCCGTCCCACTTGATTA)
3. The primer sequences of HOMX1 (Forward: ATTTCAGAAGGGCCAGGTGA Reverse: GGAAGTAGACAGGGGCGAAGA)
4. **Supplementary Figure Legends**

**S2A:**

**
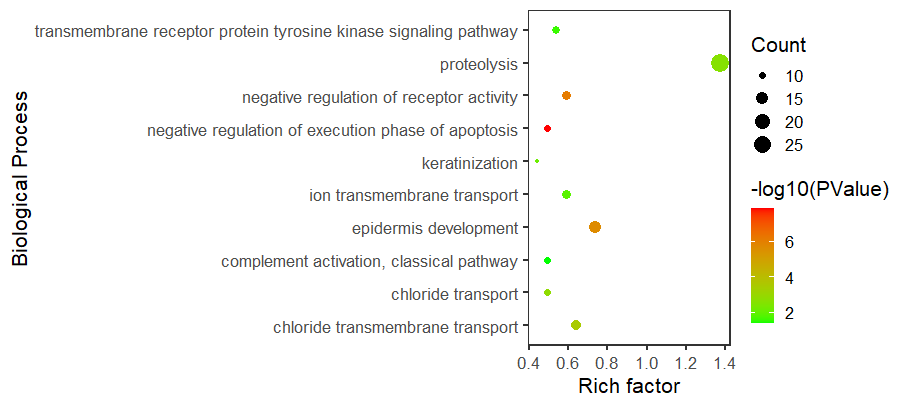
**

S2B


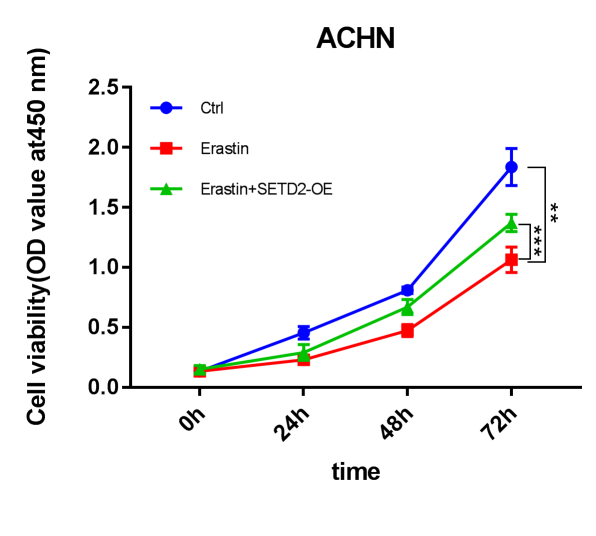


**Fig. 2: SETD2 knockdown promotes ROS levels, promotes Fe2+ accumulation, and increases erastin sensitivity**. (S2A) A GO analysis was performed by dividing SETD2 into two groups based on their median expression values. (S2B) CCK-8 assays were used to analyse the effect in the Ctrl, erastin as well as SETD2-OE groups on cell viability in ACHN

S4A:


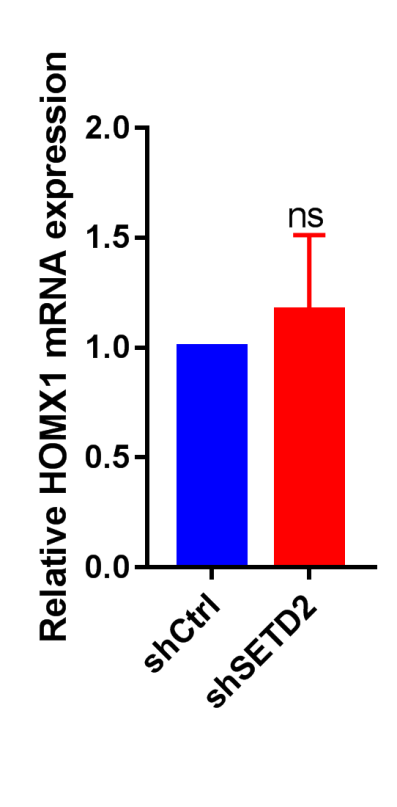


S4B:


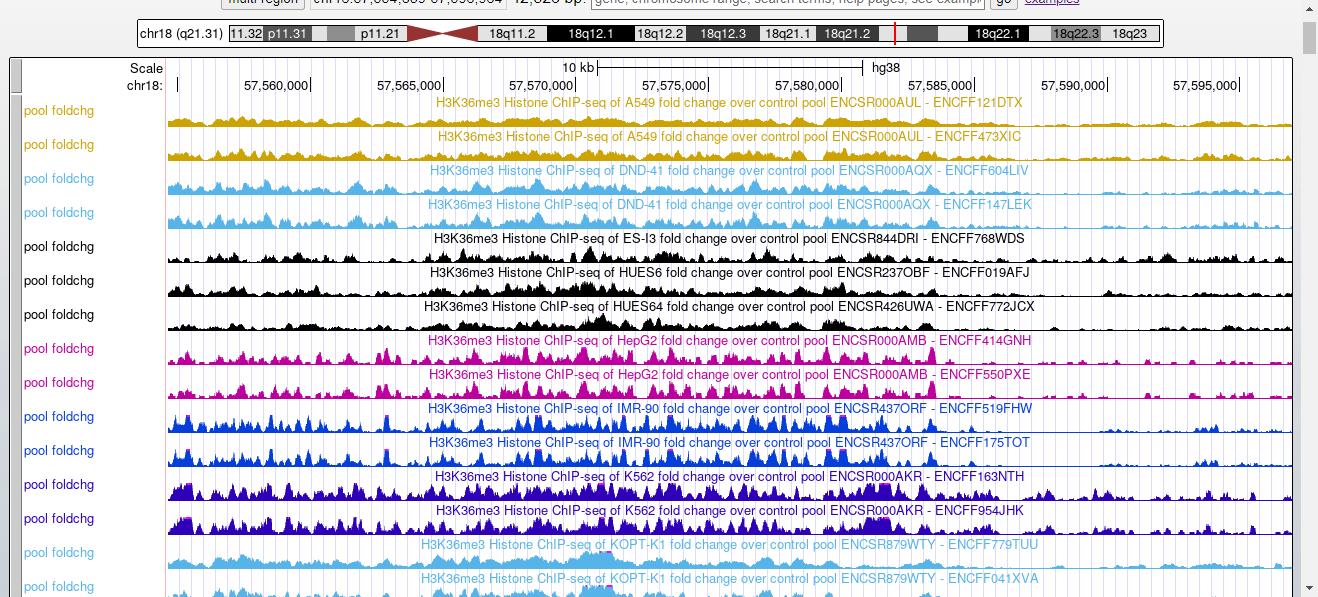


**Fig. 4 Knockdown of SETD2 increases erastin-induced ferroptosis sensitivity mainly through the H3K36me3/FECH pathway in ccRCC** (S4A) qRT-PCR to analyze the change of HOMX1 caused by shSETD2. (S4B) Prediction of possible interaction between H3K36me3 and FECH from ENCODE database
