# Supplementary material for: Knockdown of SETD2 promotes erastin-induced ferroptosis in ccRCC
Source: Cell Death Dis. 2023 Aug 21;14(8):539. doi: 10.1038/s41419-023-06057-8 (PMC10442429; doi:10.1038/s41419-023-06057-8)

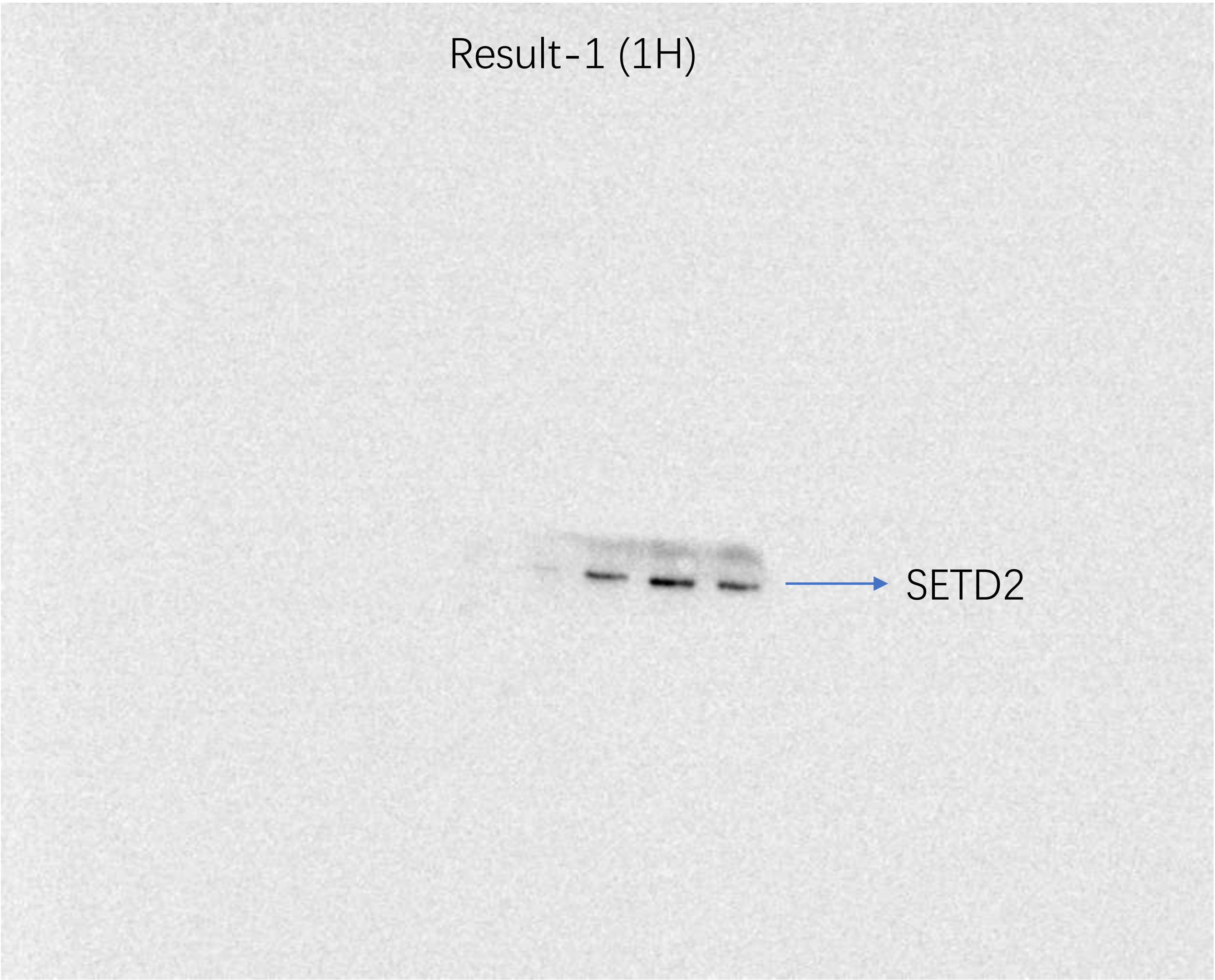
Result-1 (1H)
SETD2

Result-1 (1H)

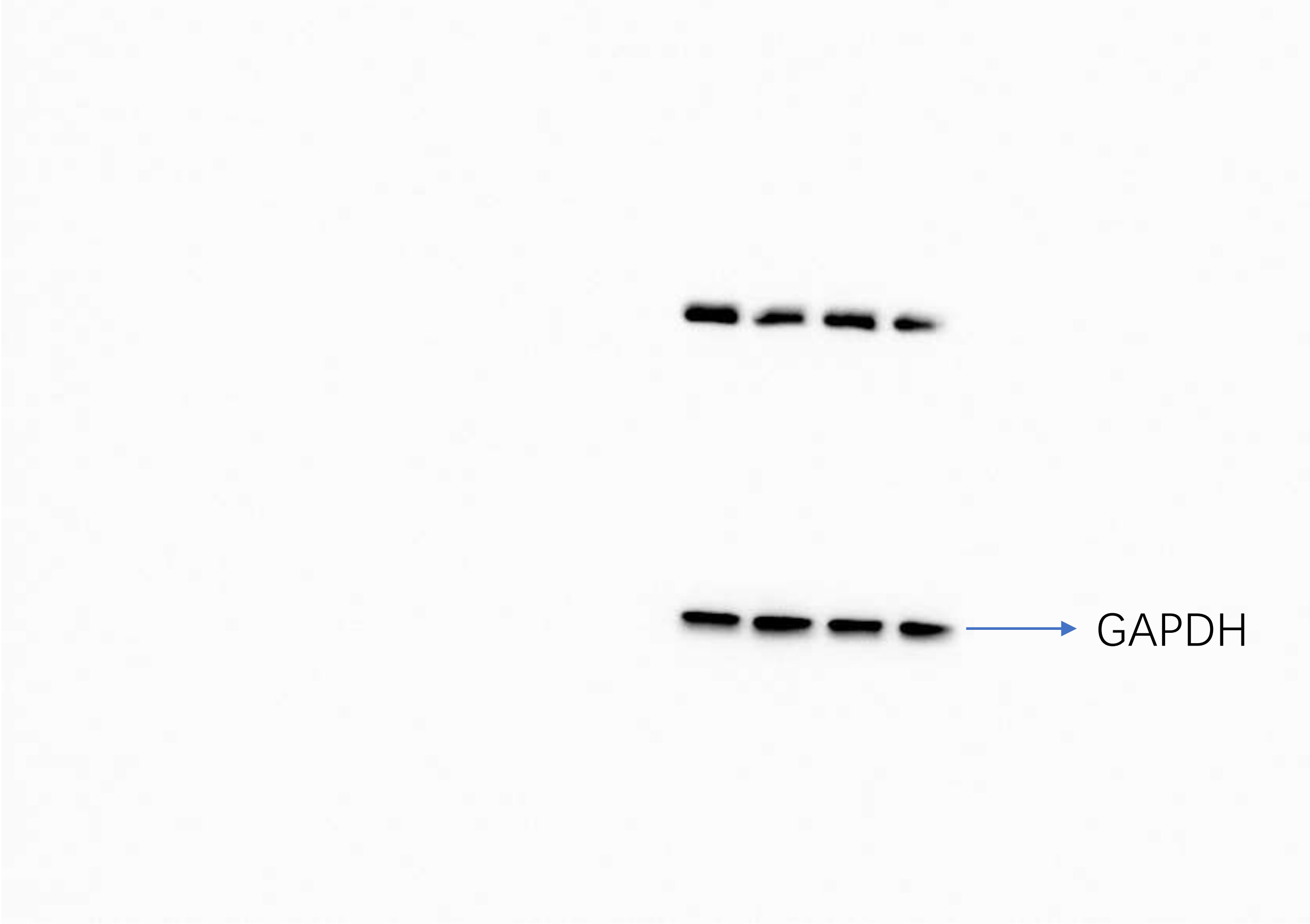

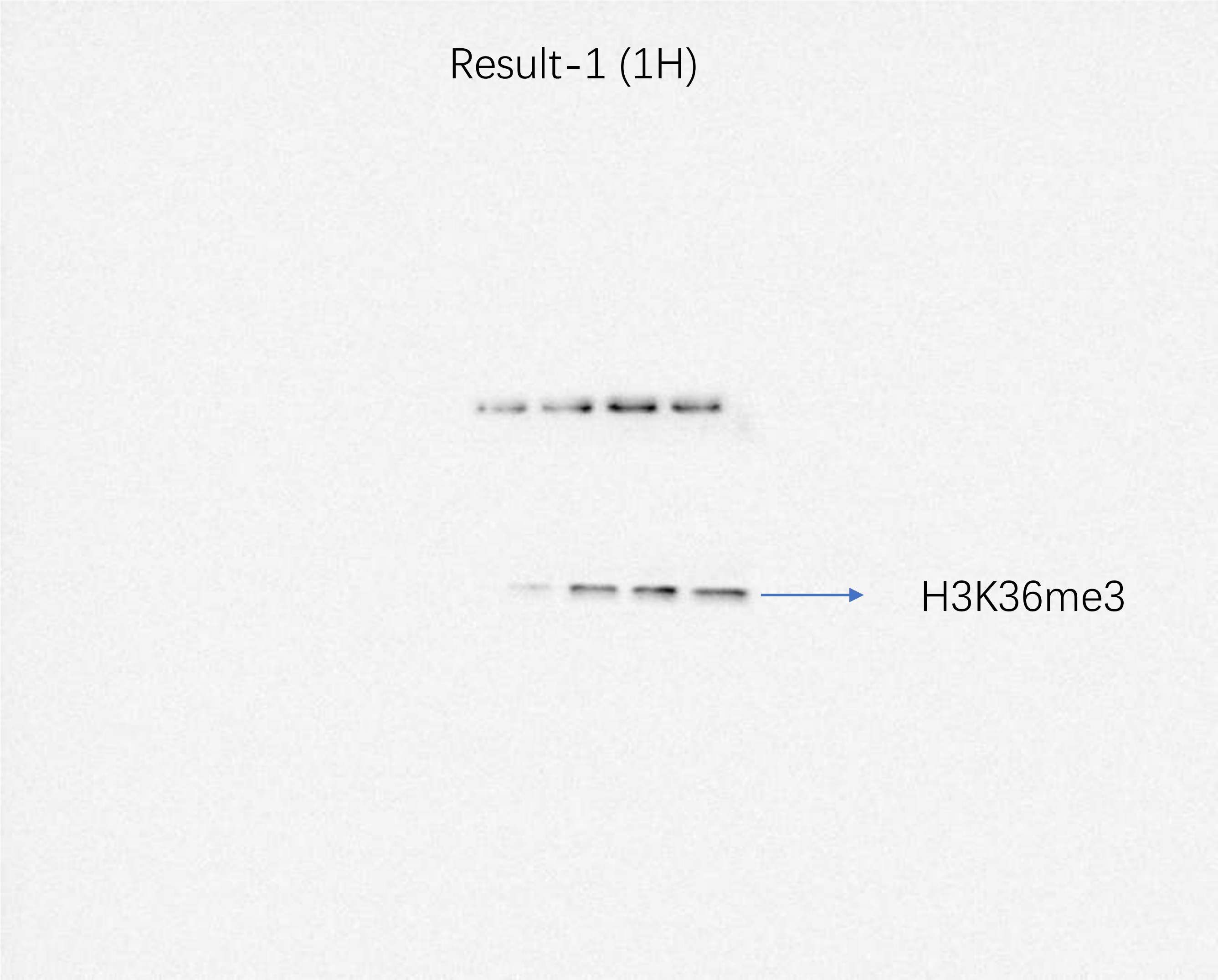
Result-1 (1H)
H3K36me3

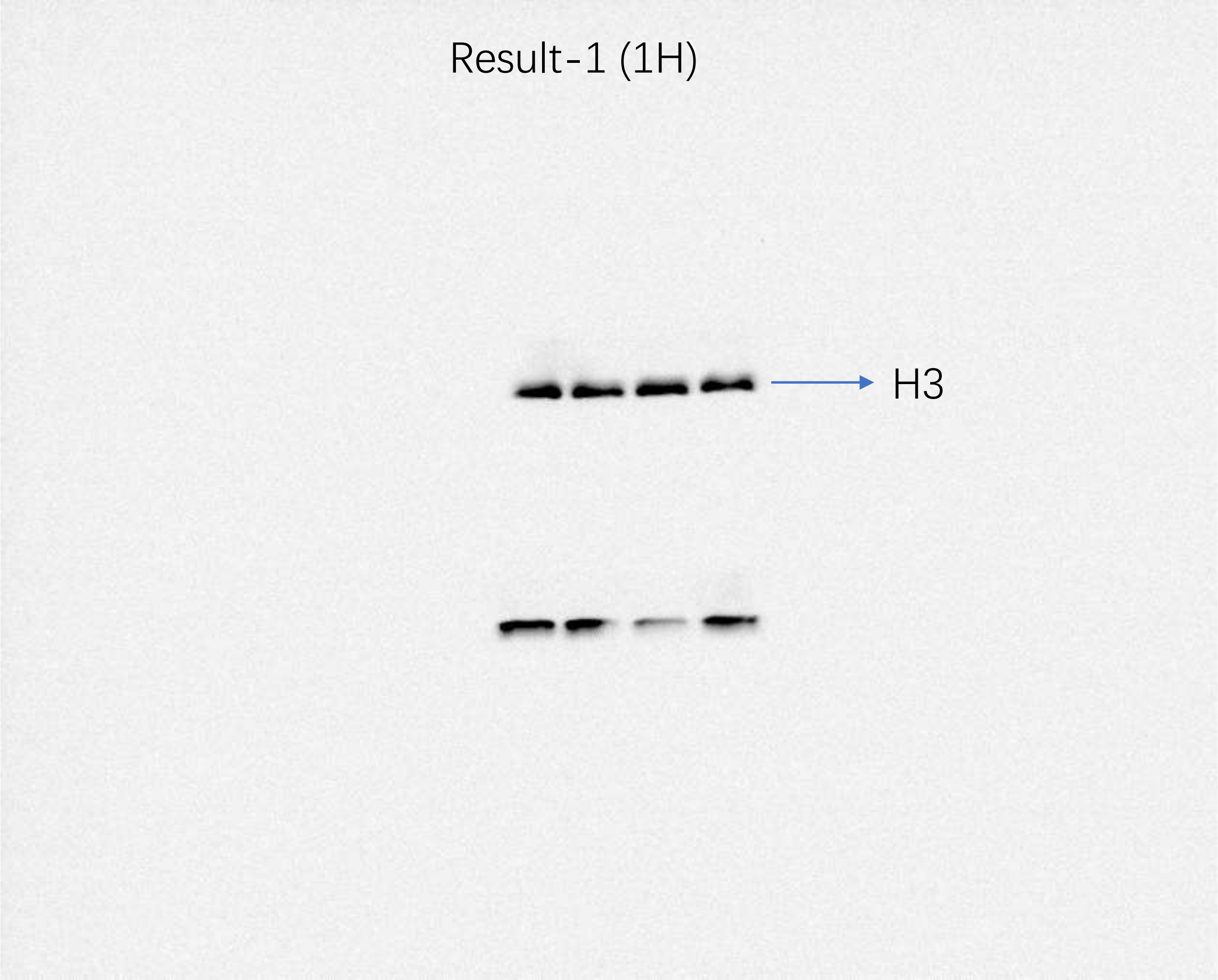
Result-1 (1H)
H3

Result-2 (2G)

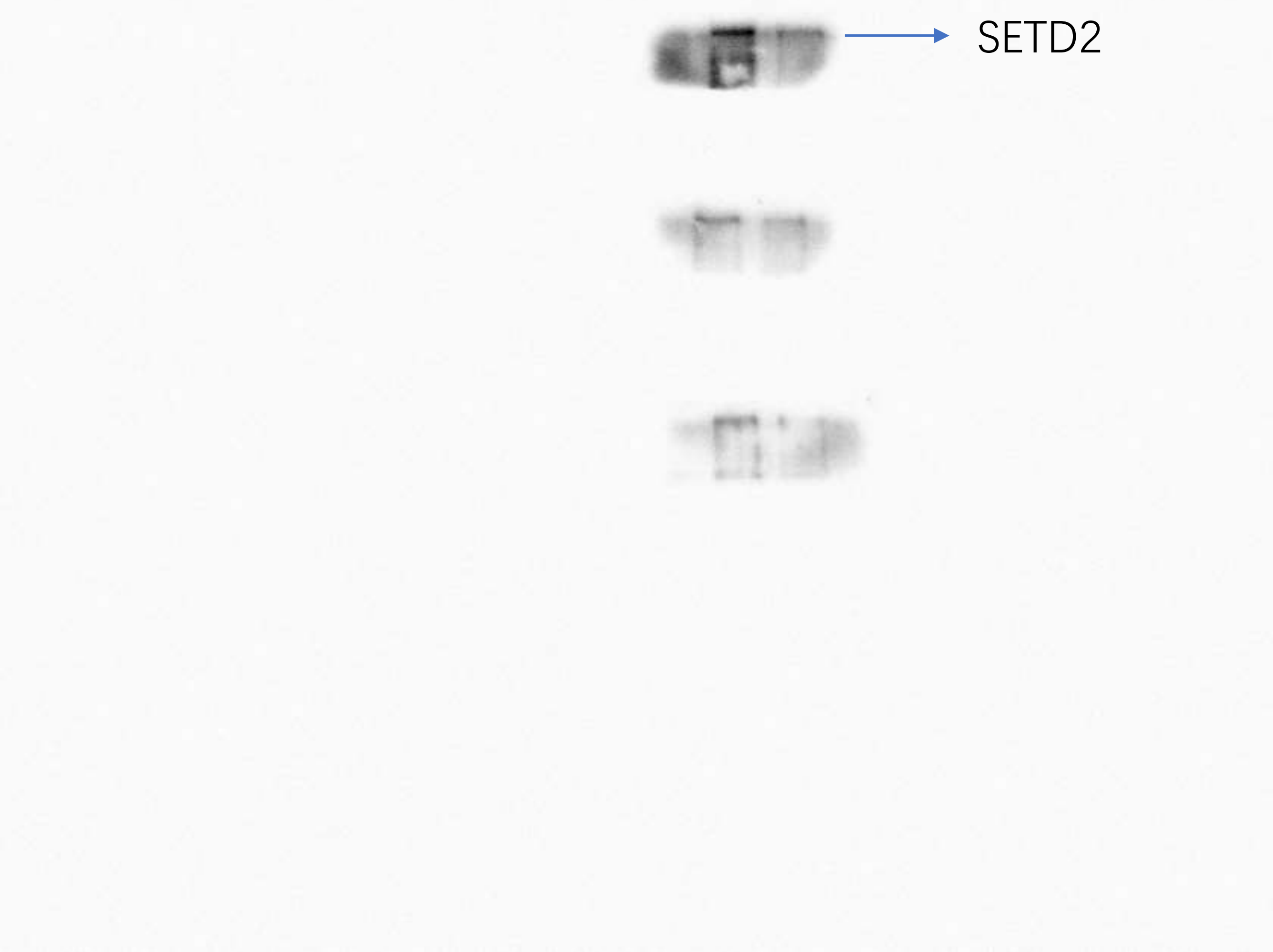

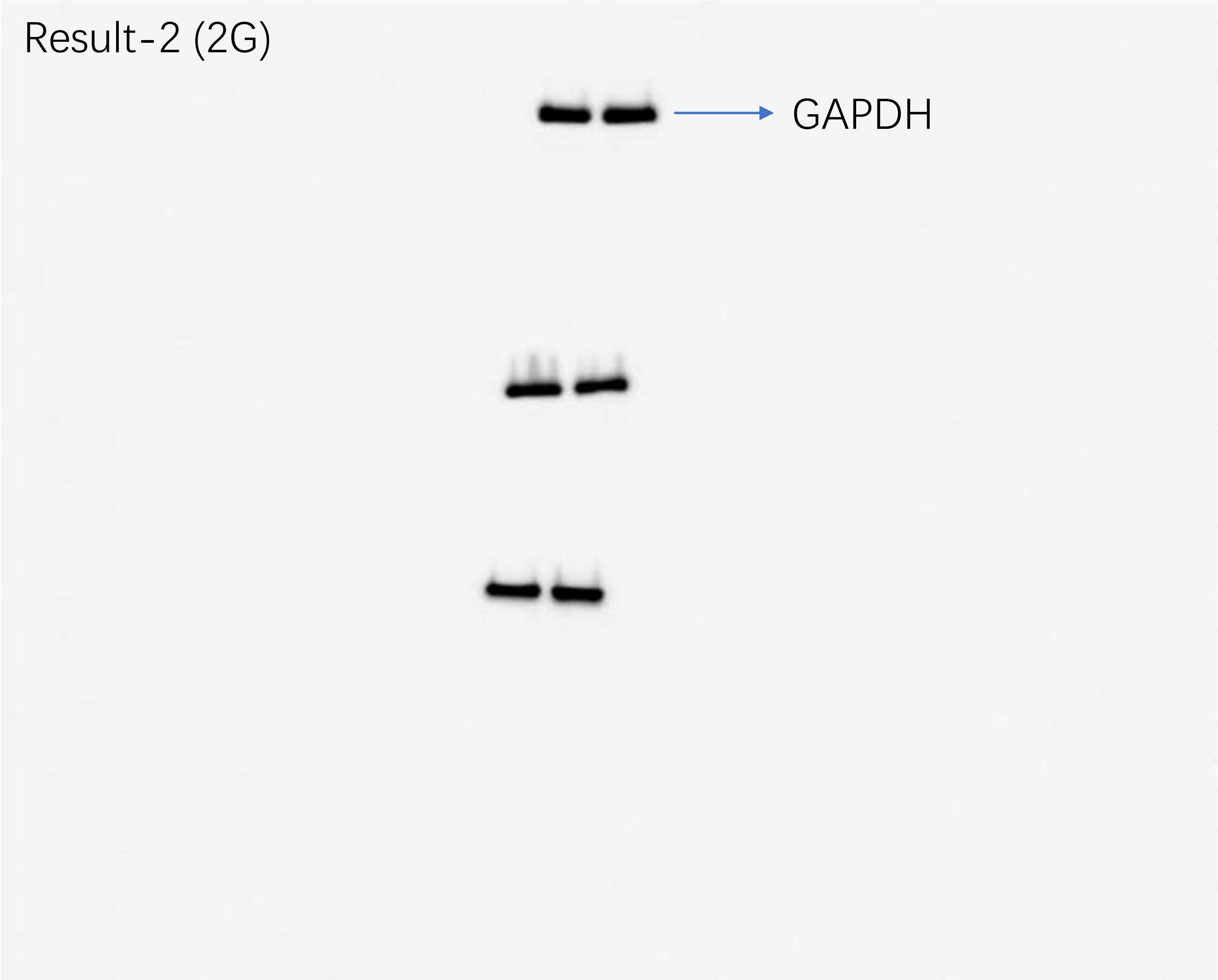
Result-2 (2G)
GAPDH

Result-2 (2G)

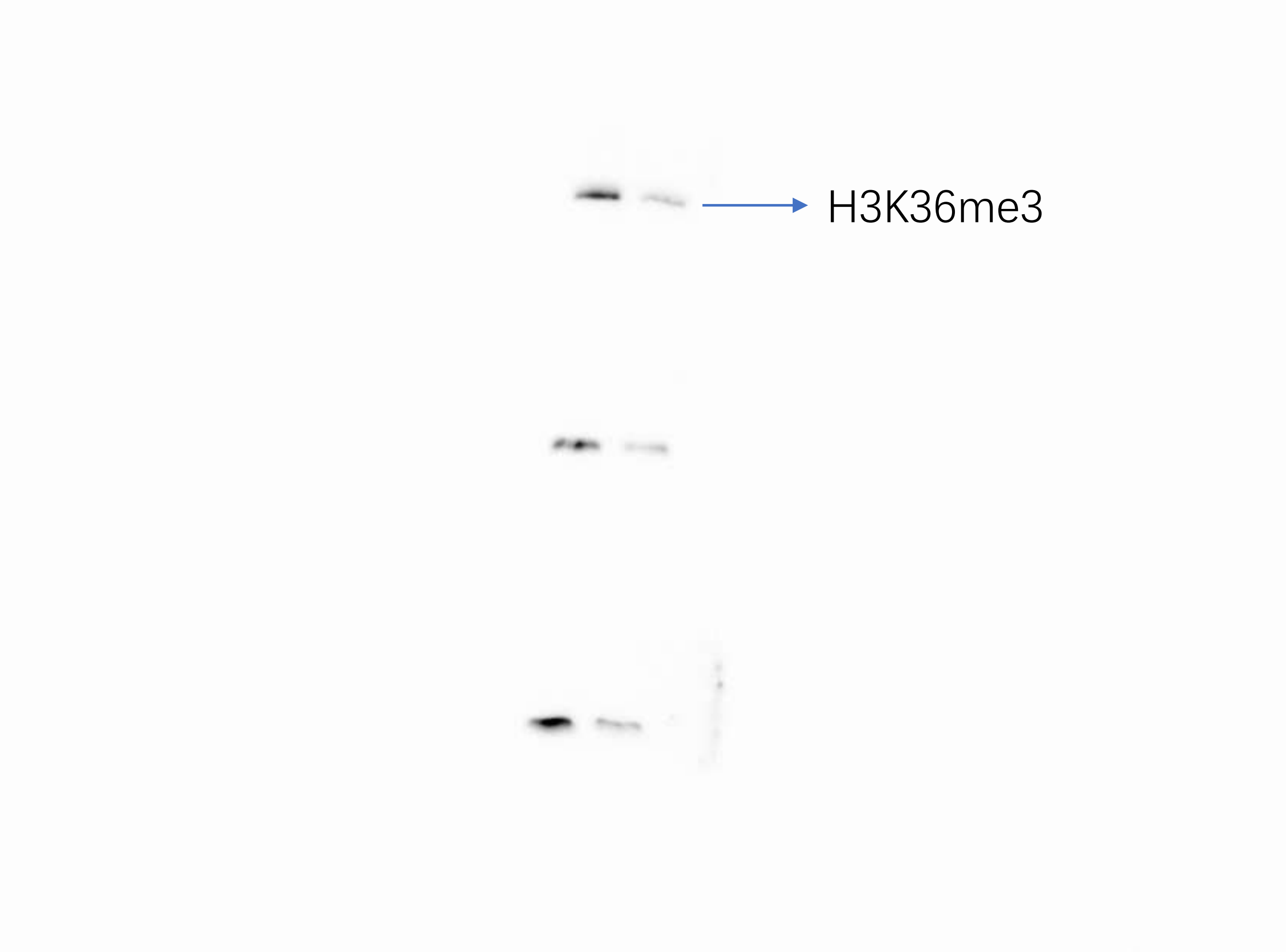

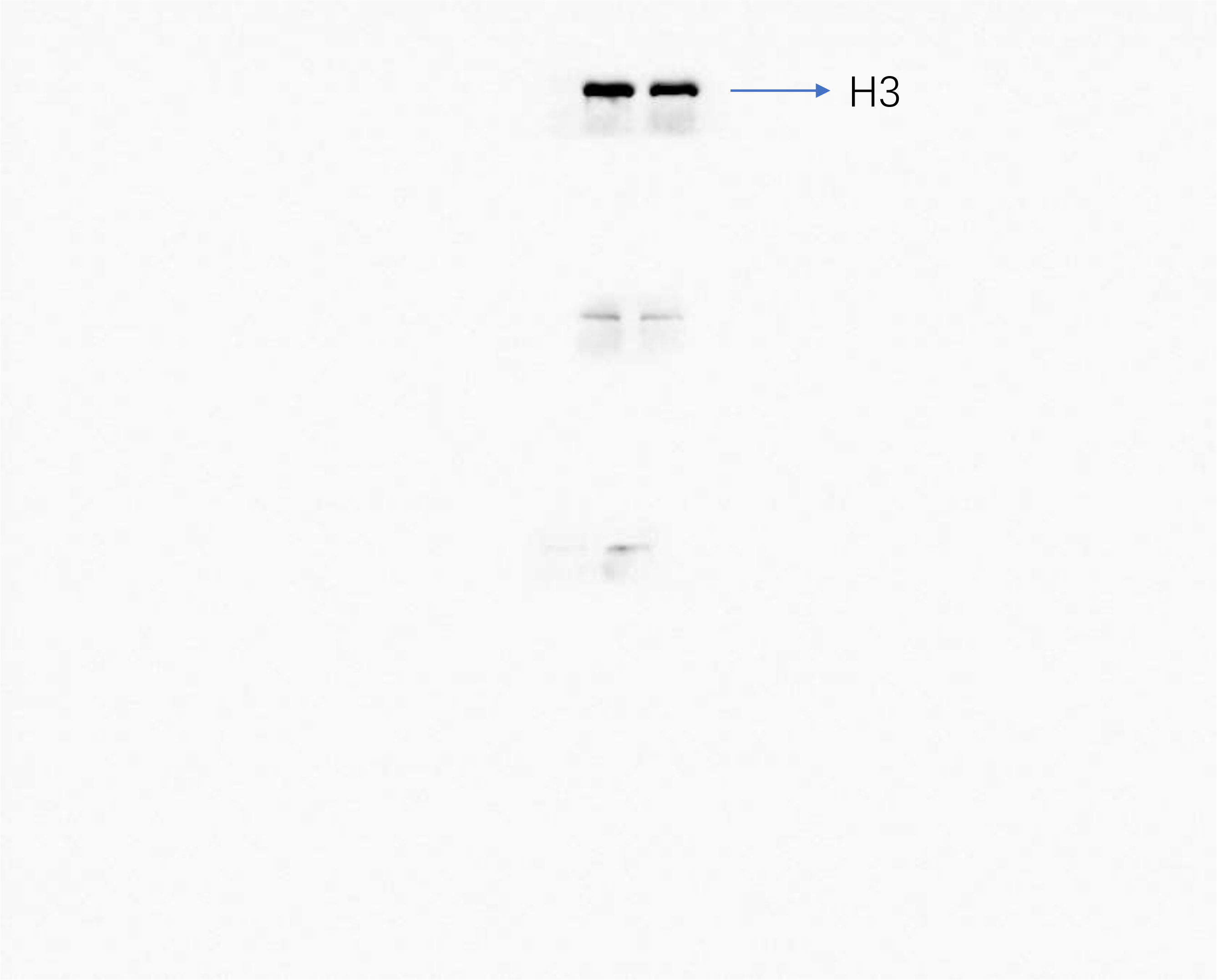
H3

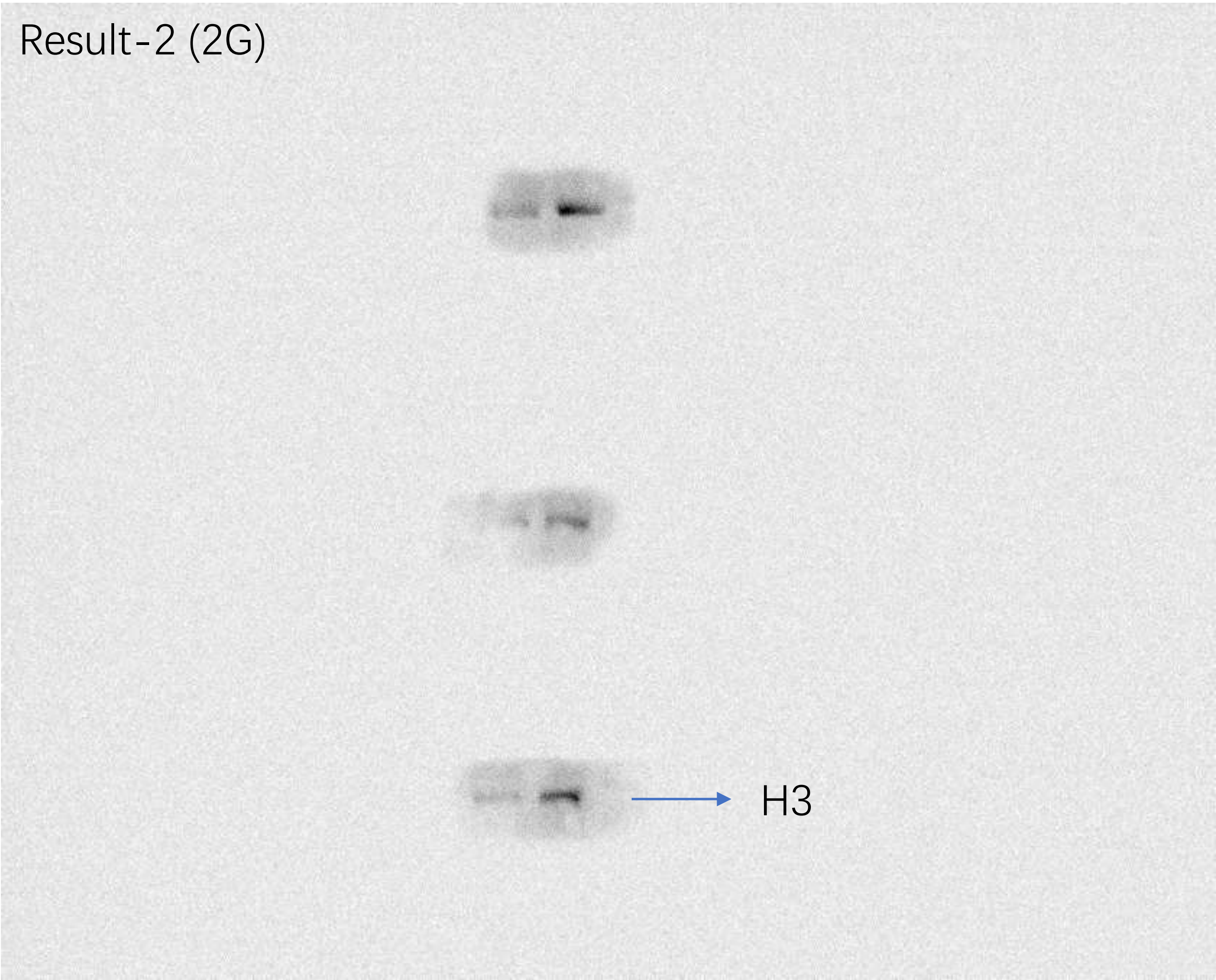
Result-2 (2G)
H3

Result-2 (2G)

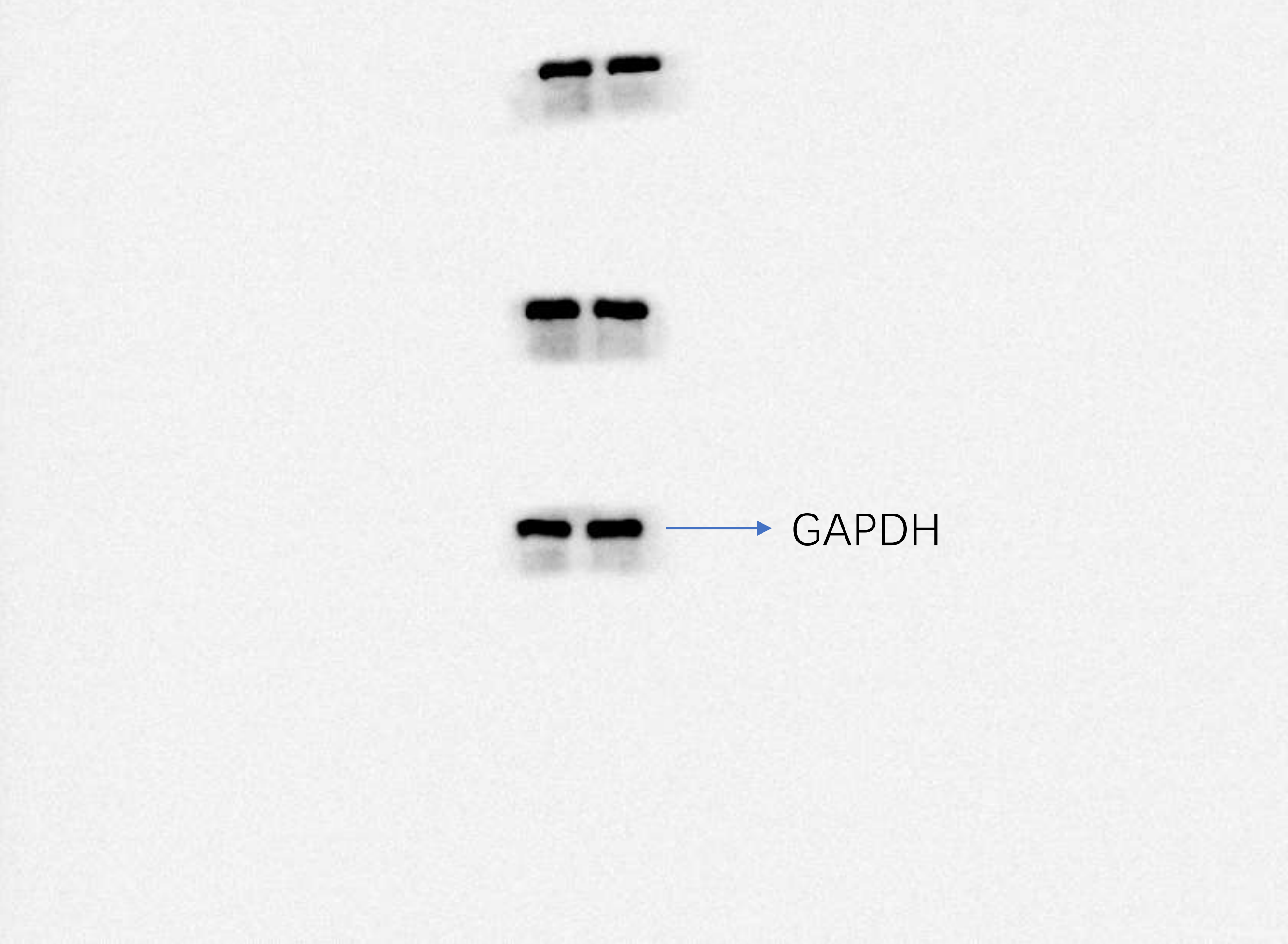

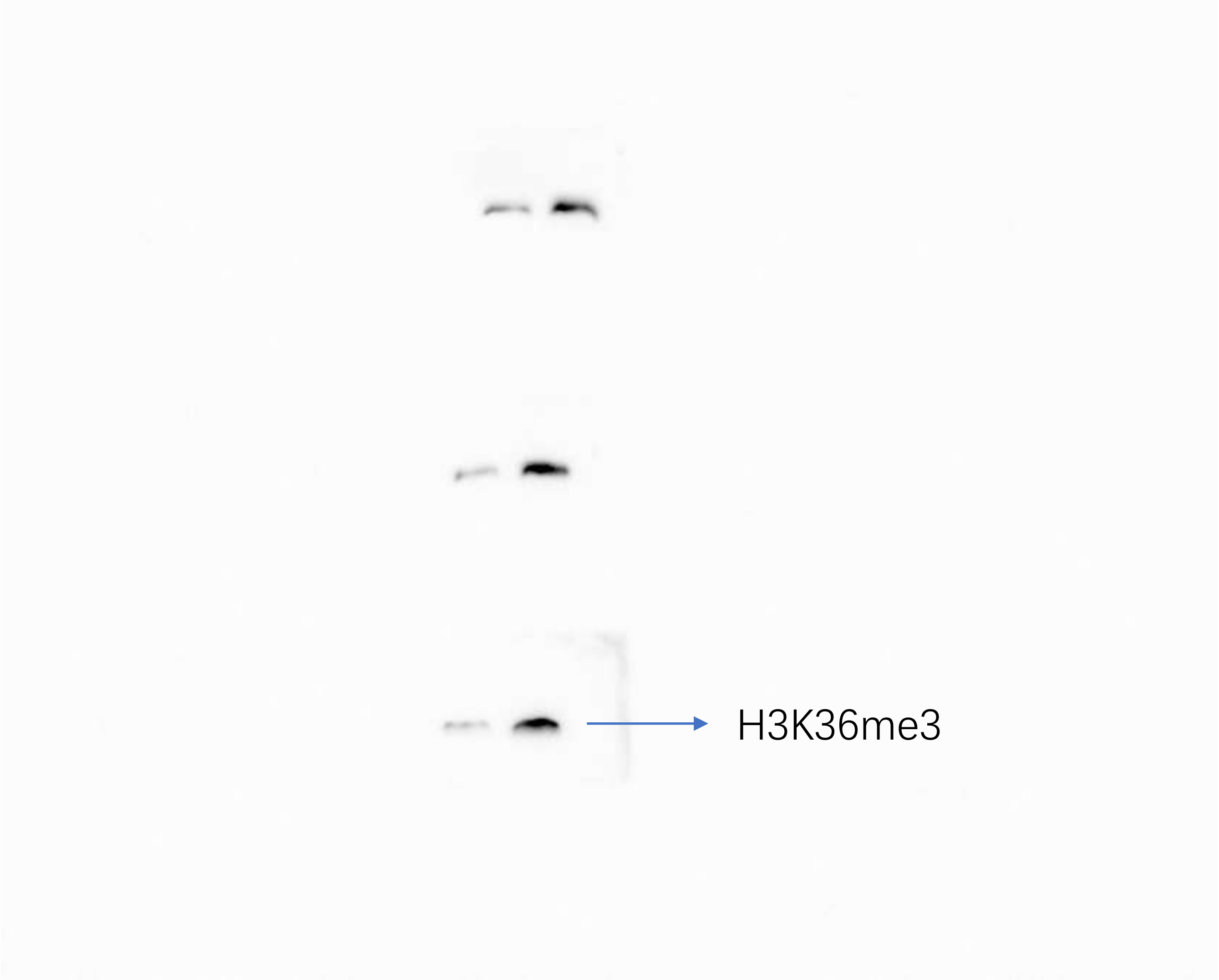
H3K36me3

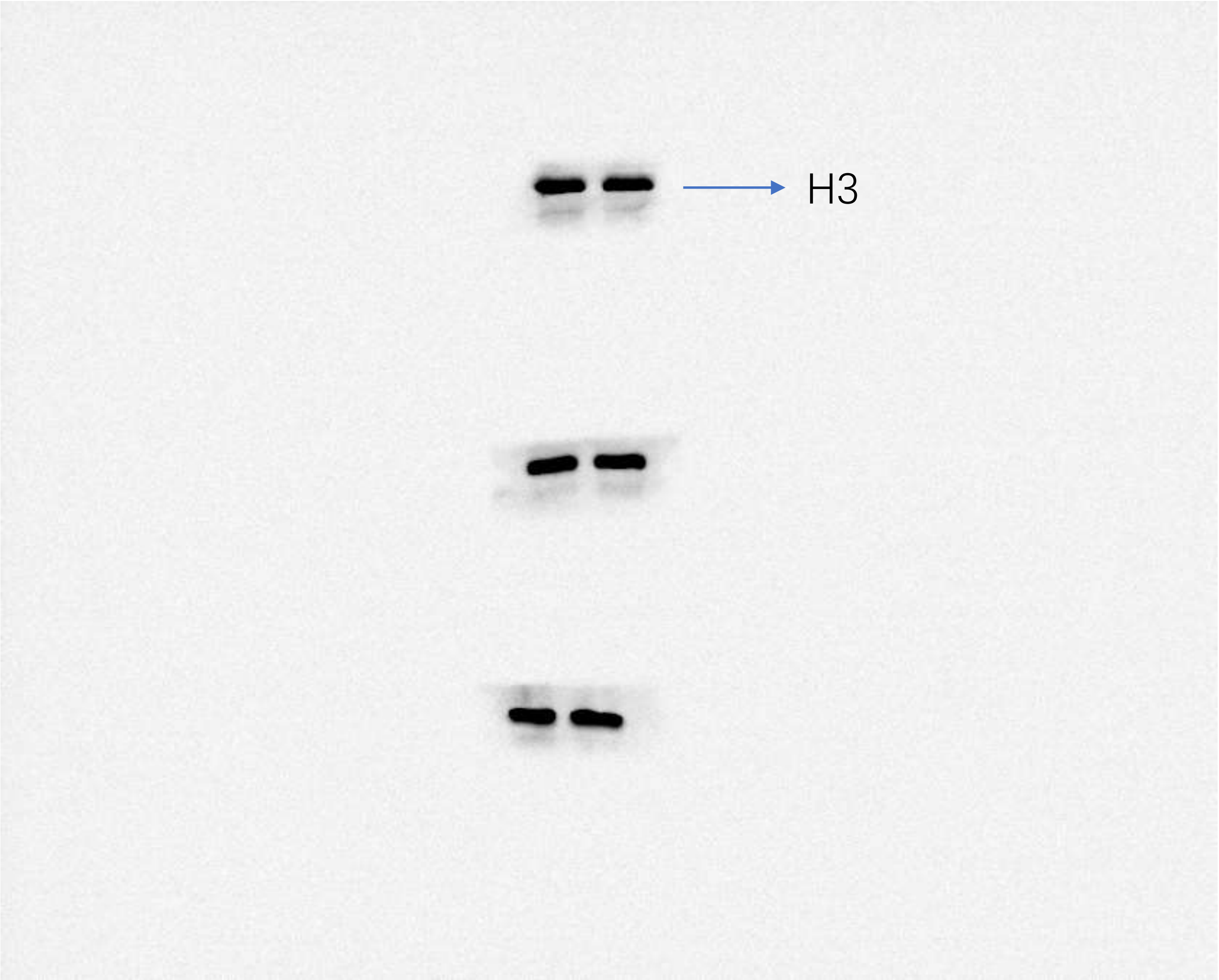
H3

Result-3 (3B)

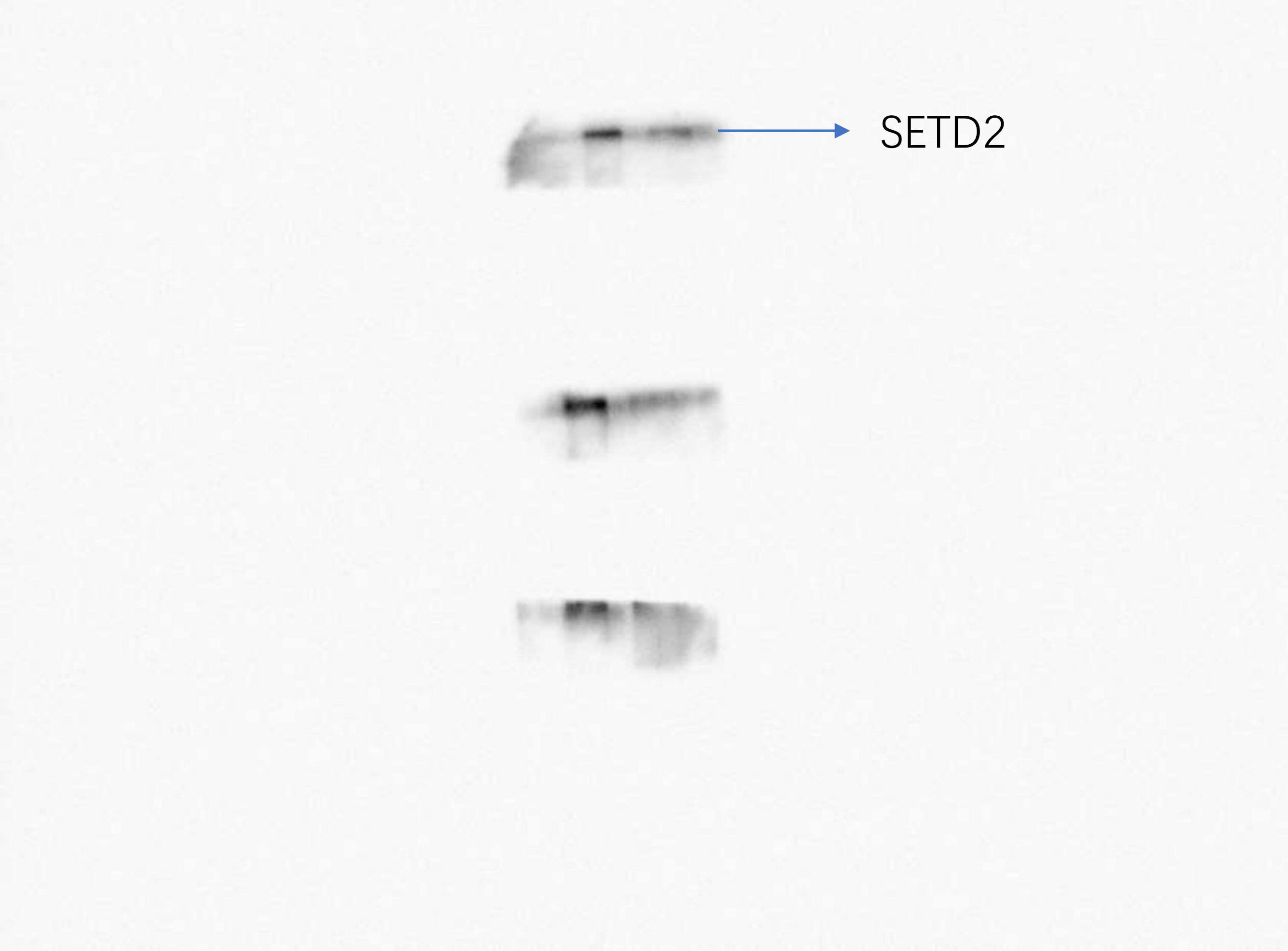

Result-3 (3B)

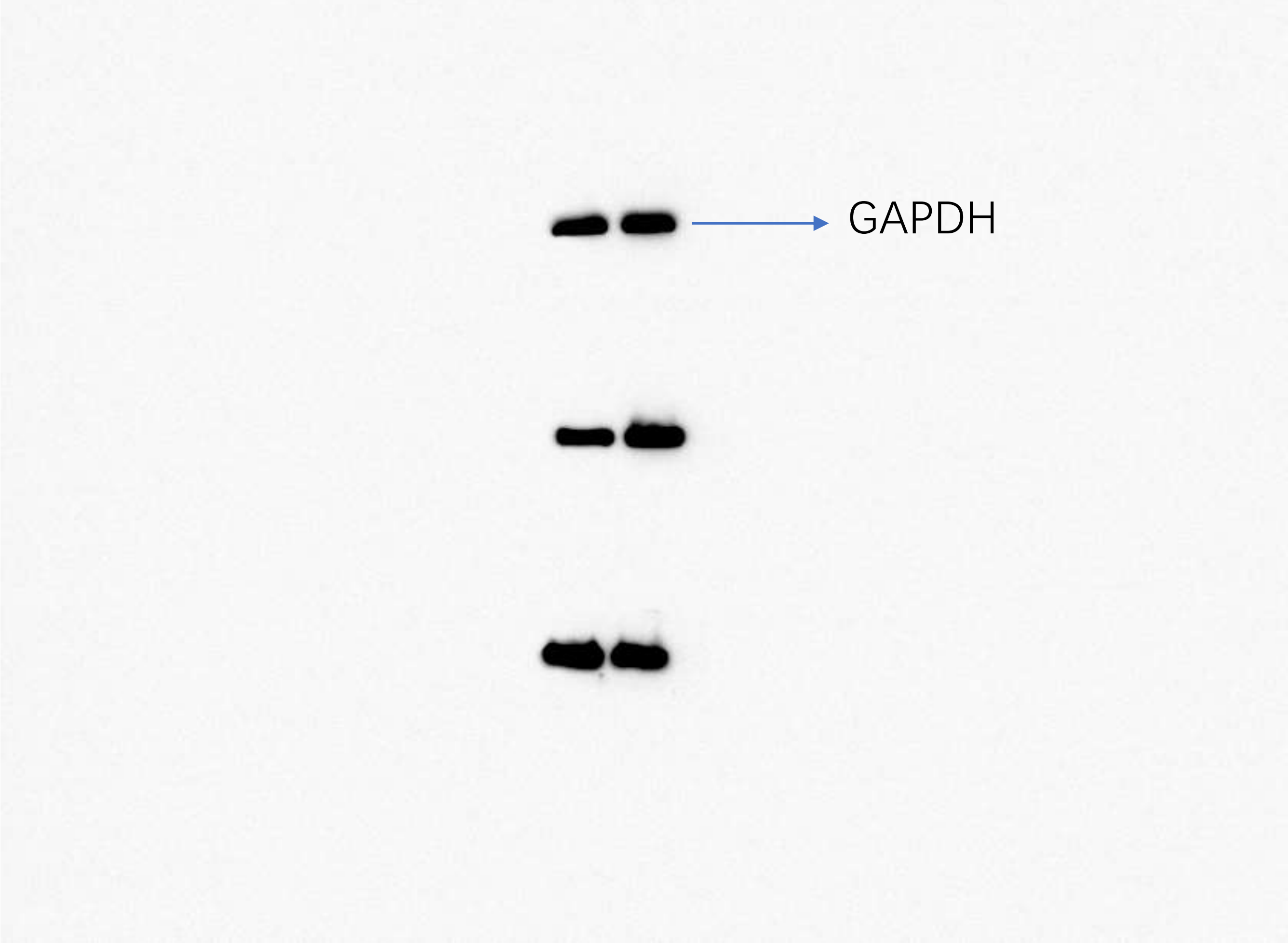

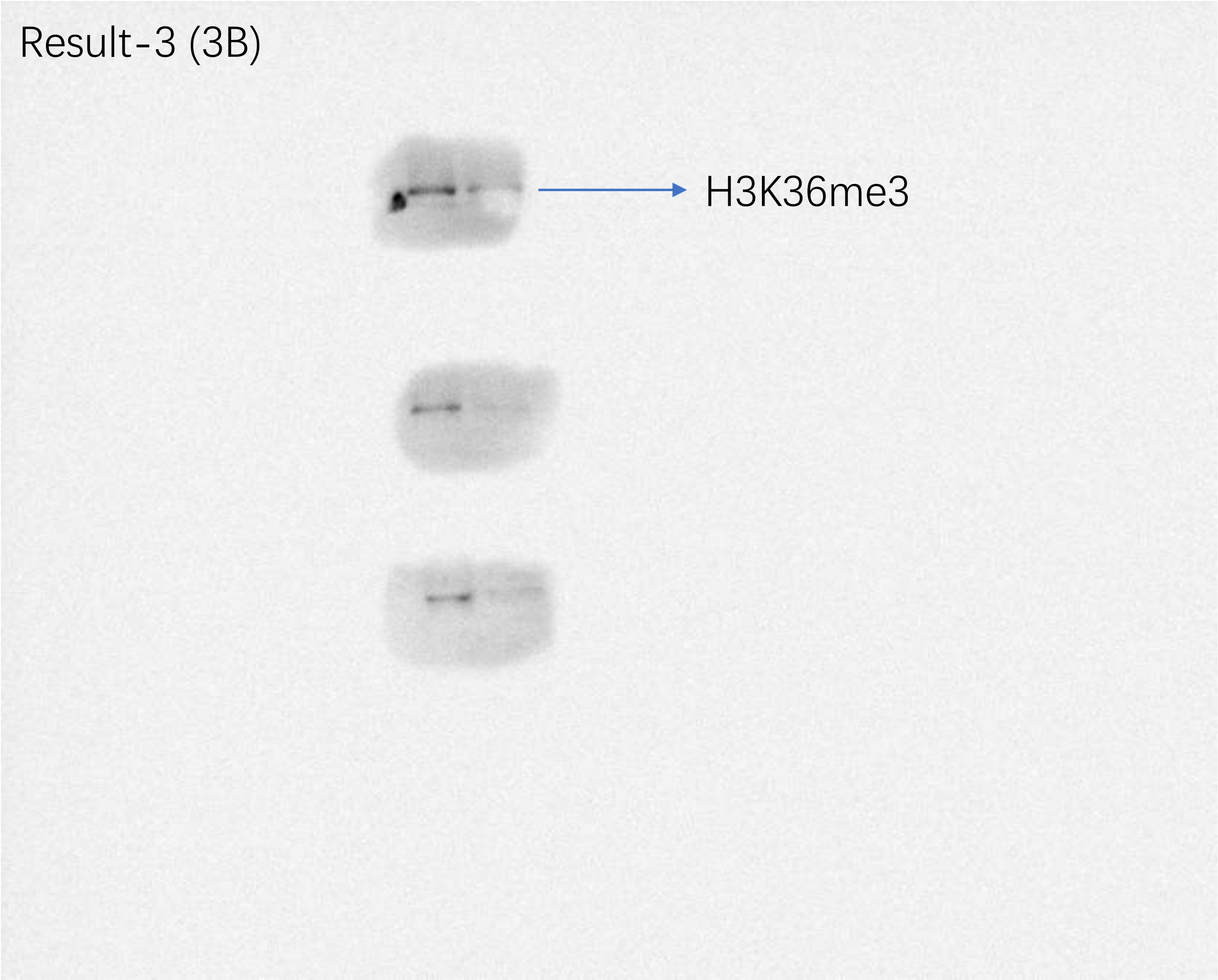
Result-3 (3B)
H3K36me3

Result-3 (3B)

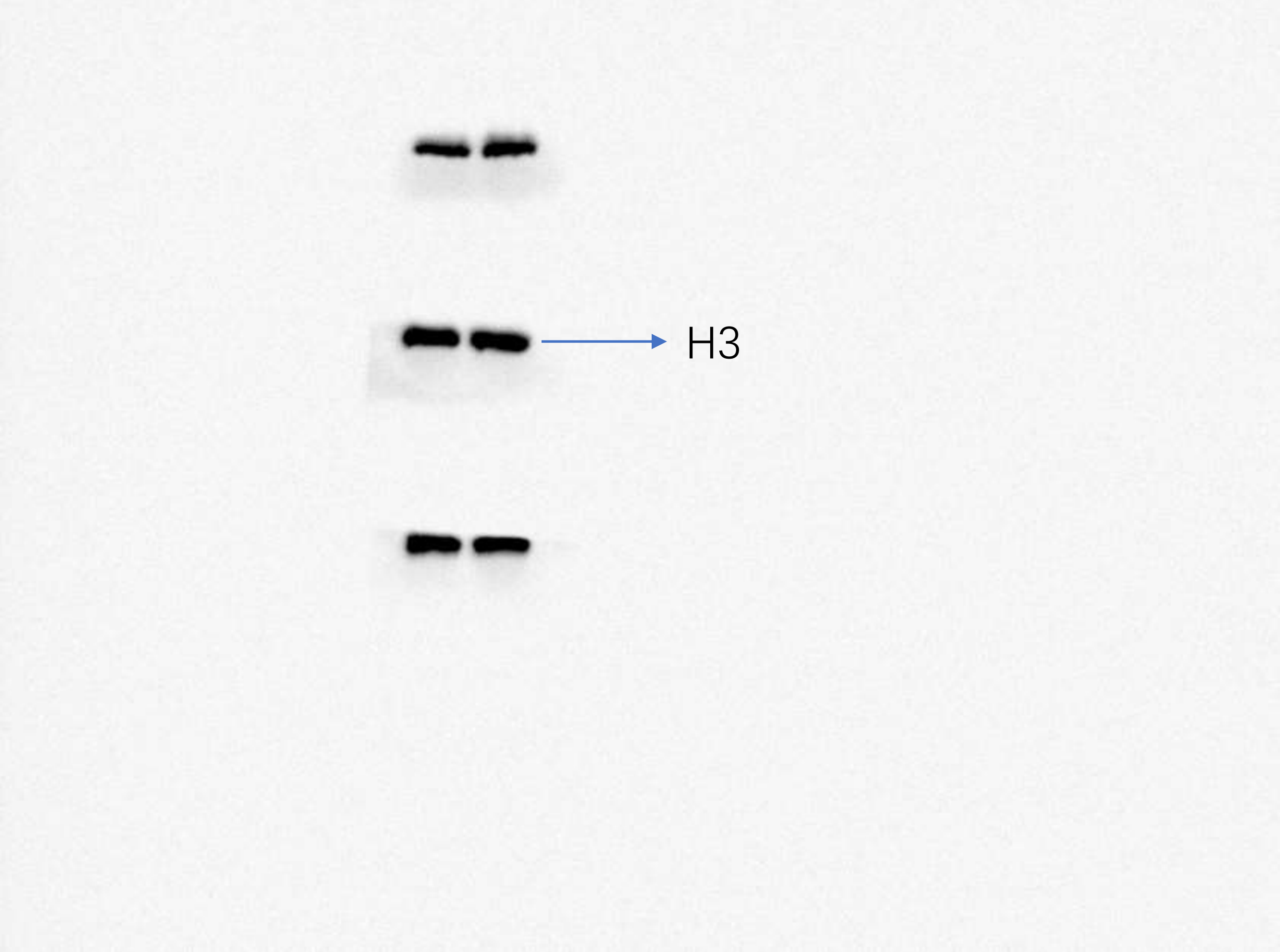

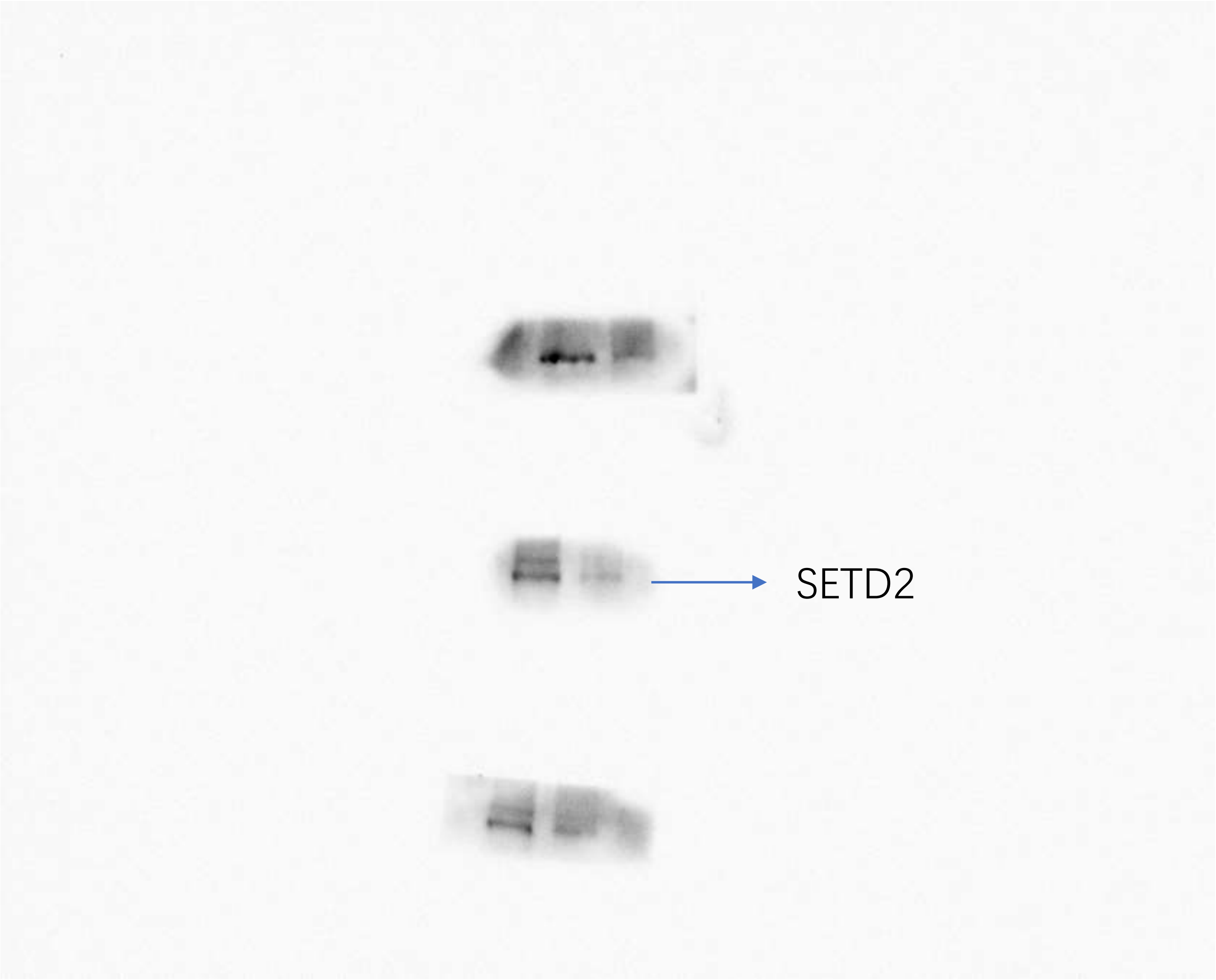
SETD2

Result-3 (3B)

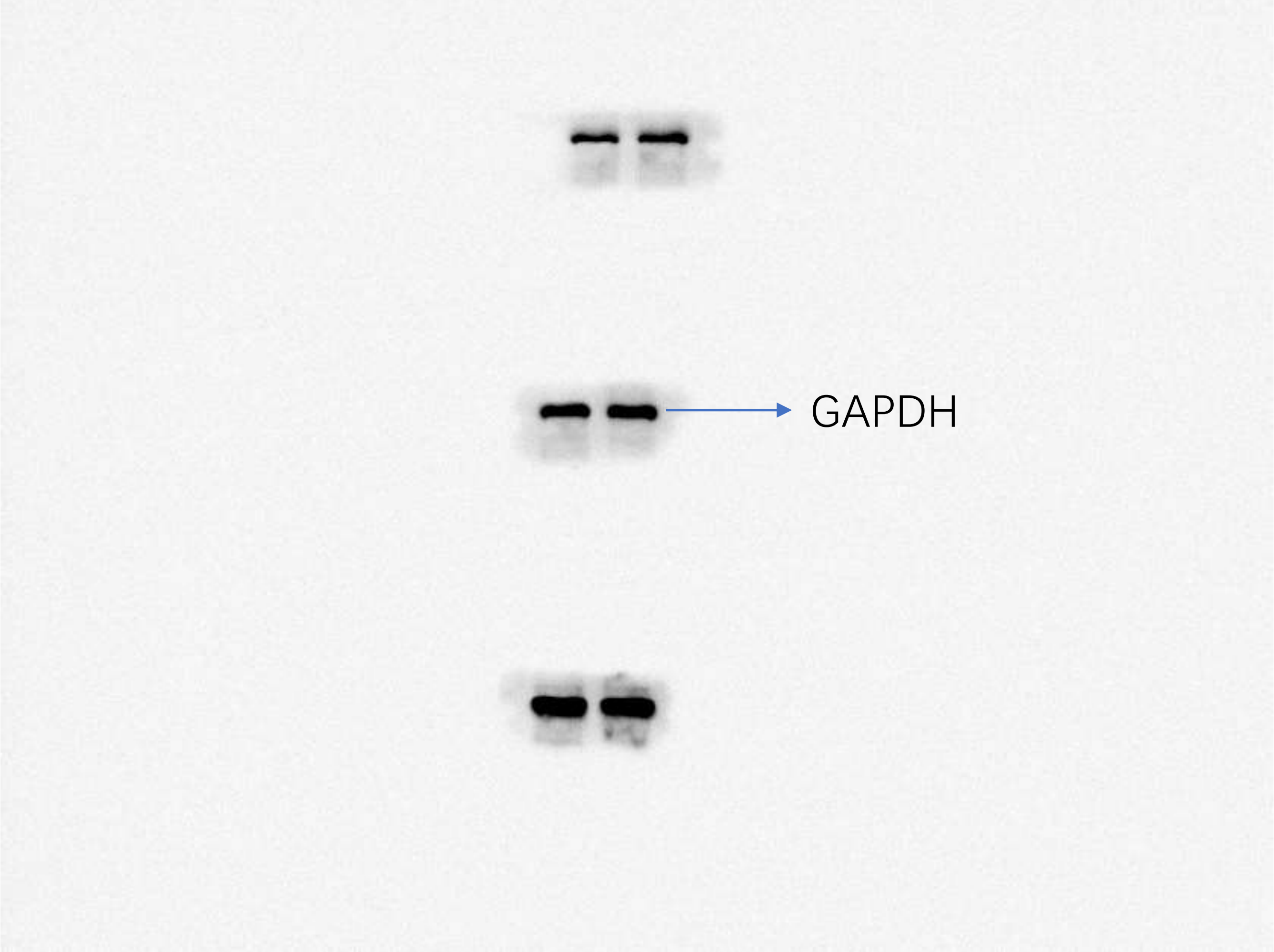

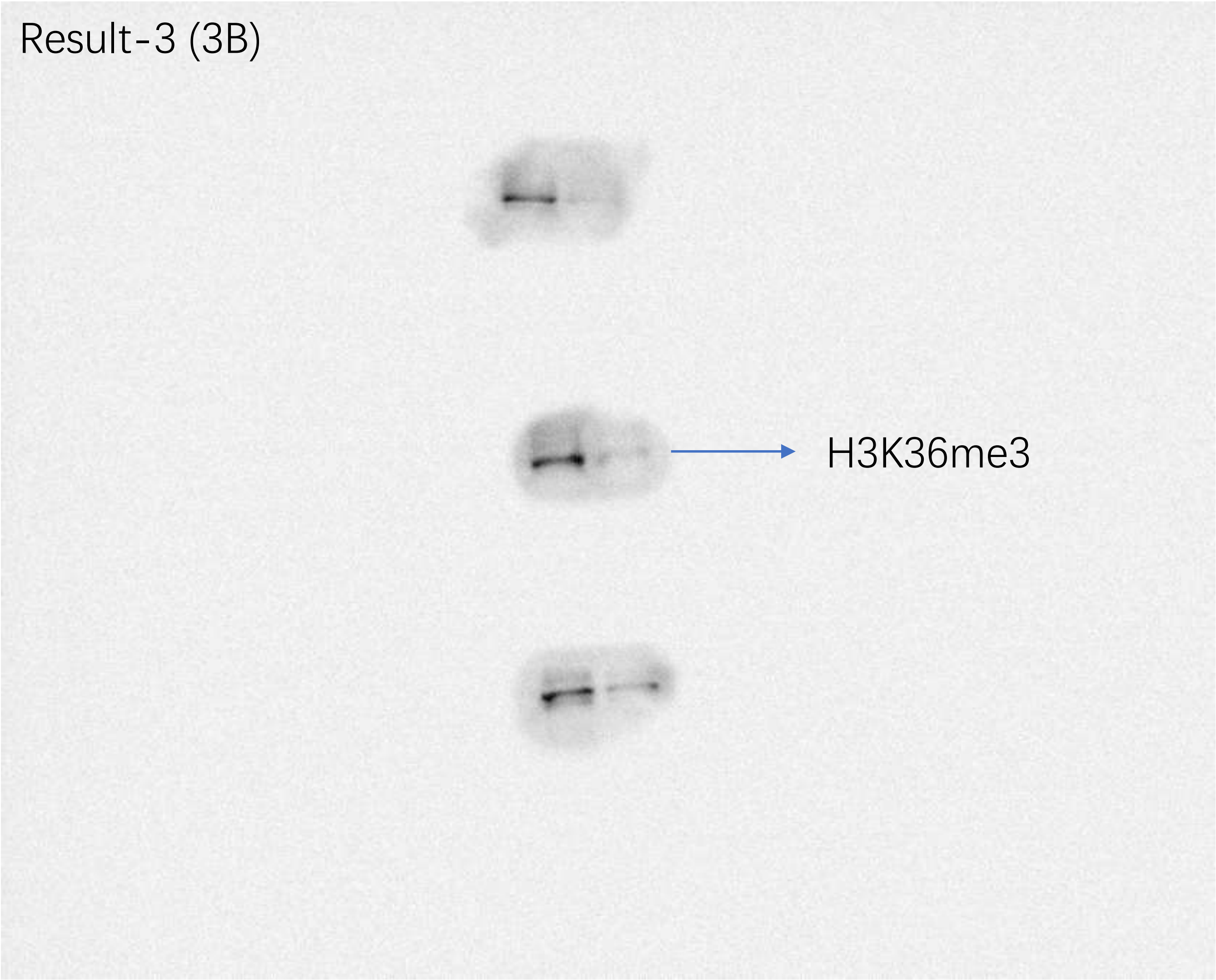
Result-3 (3B)
H3K36me3

Result-3 (3B)

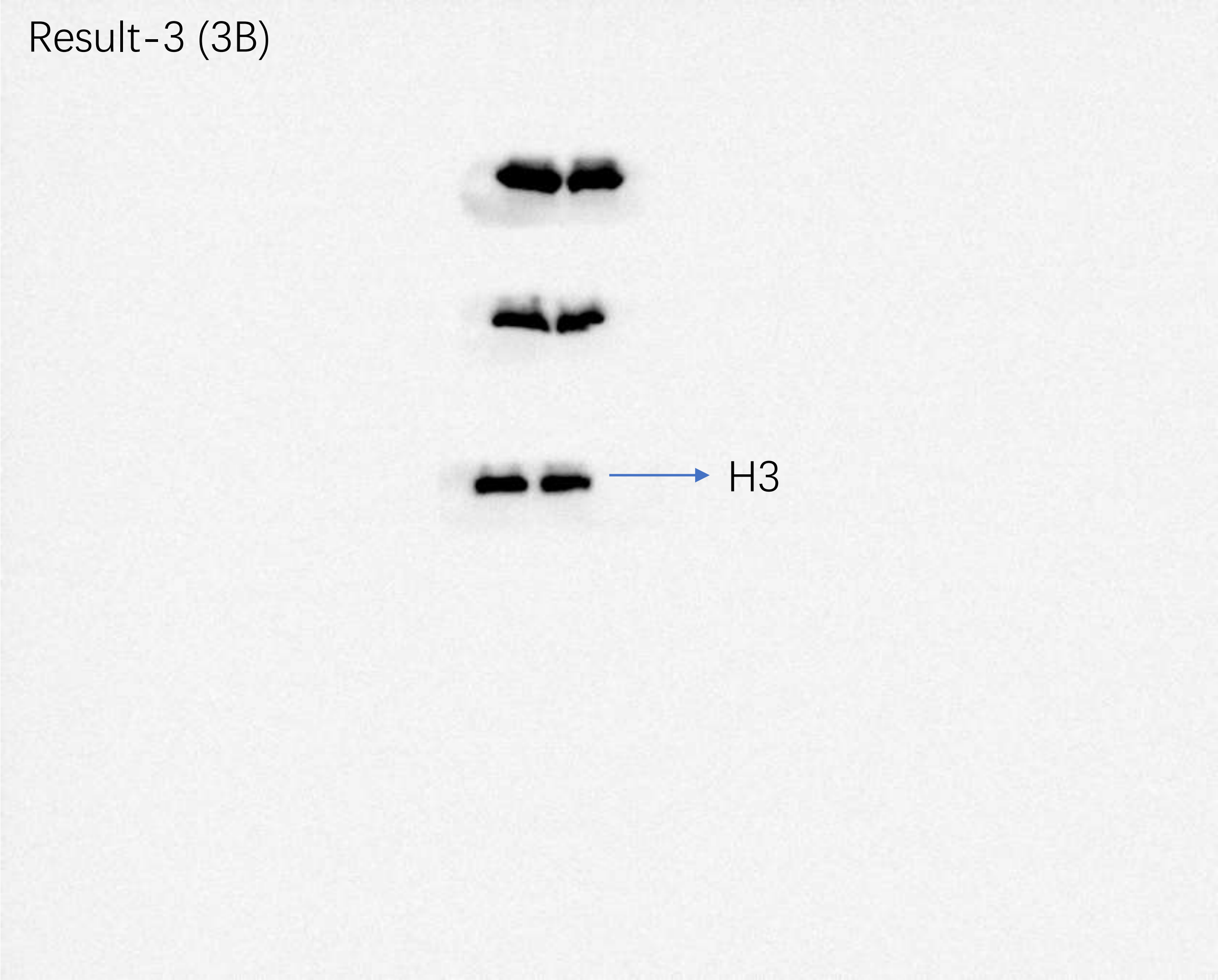

Result-3 (3H)

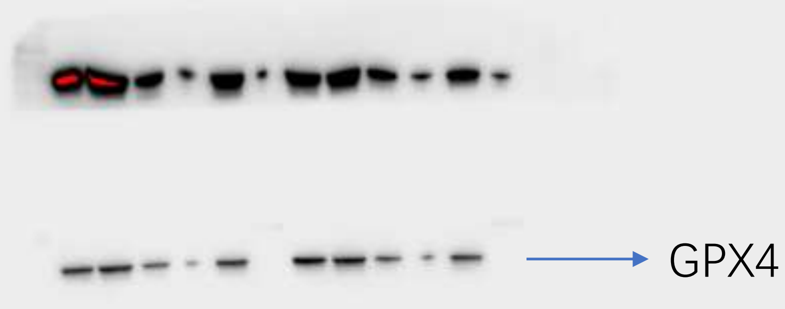

Result-3 (3H)

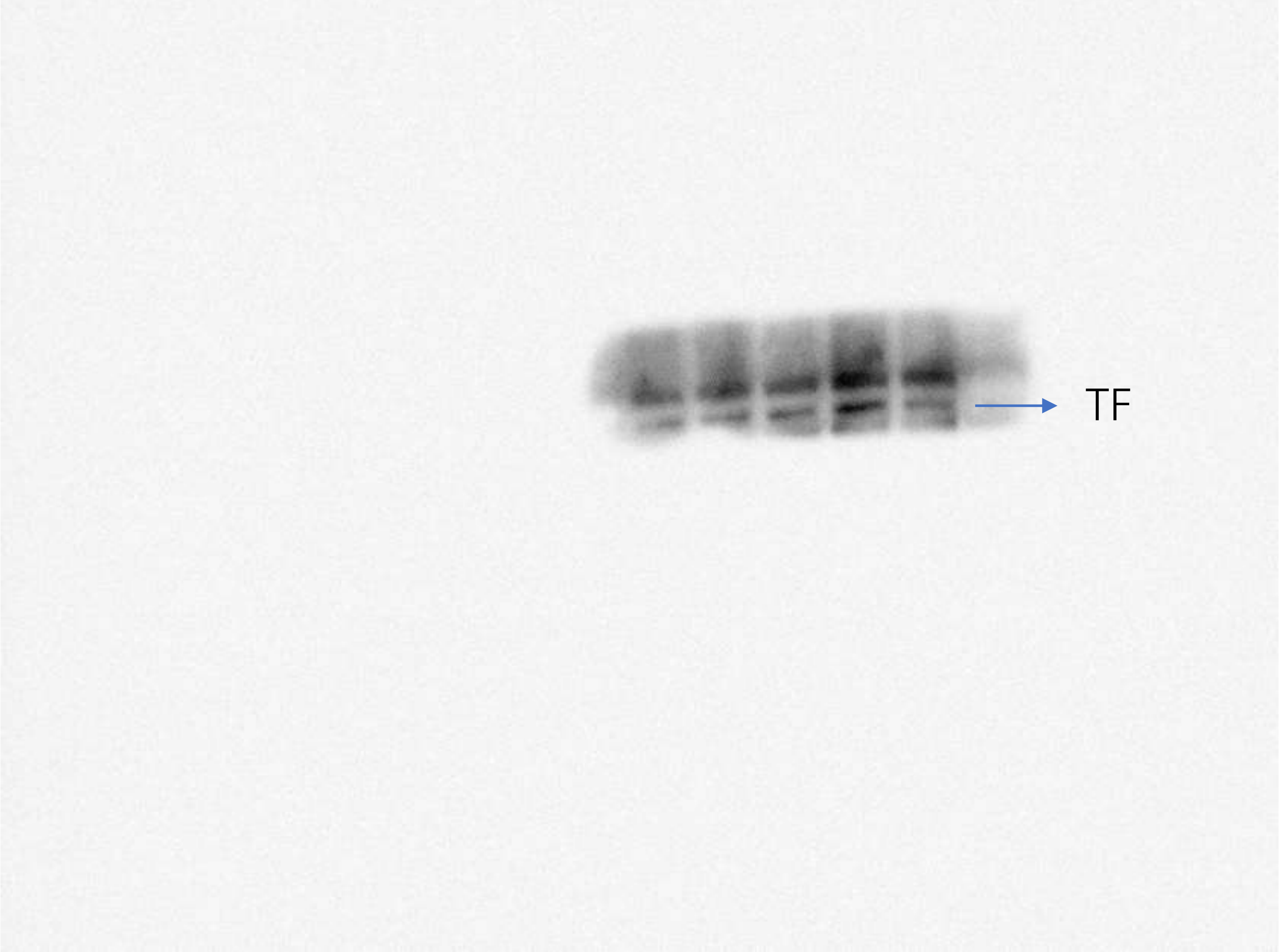

Result-3 (3H)

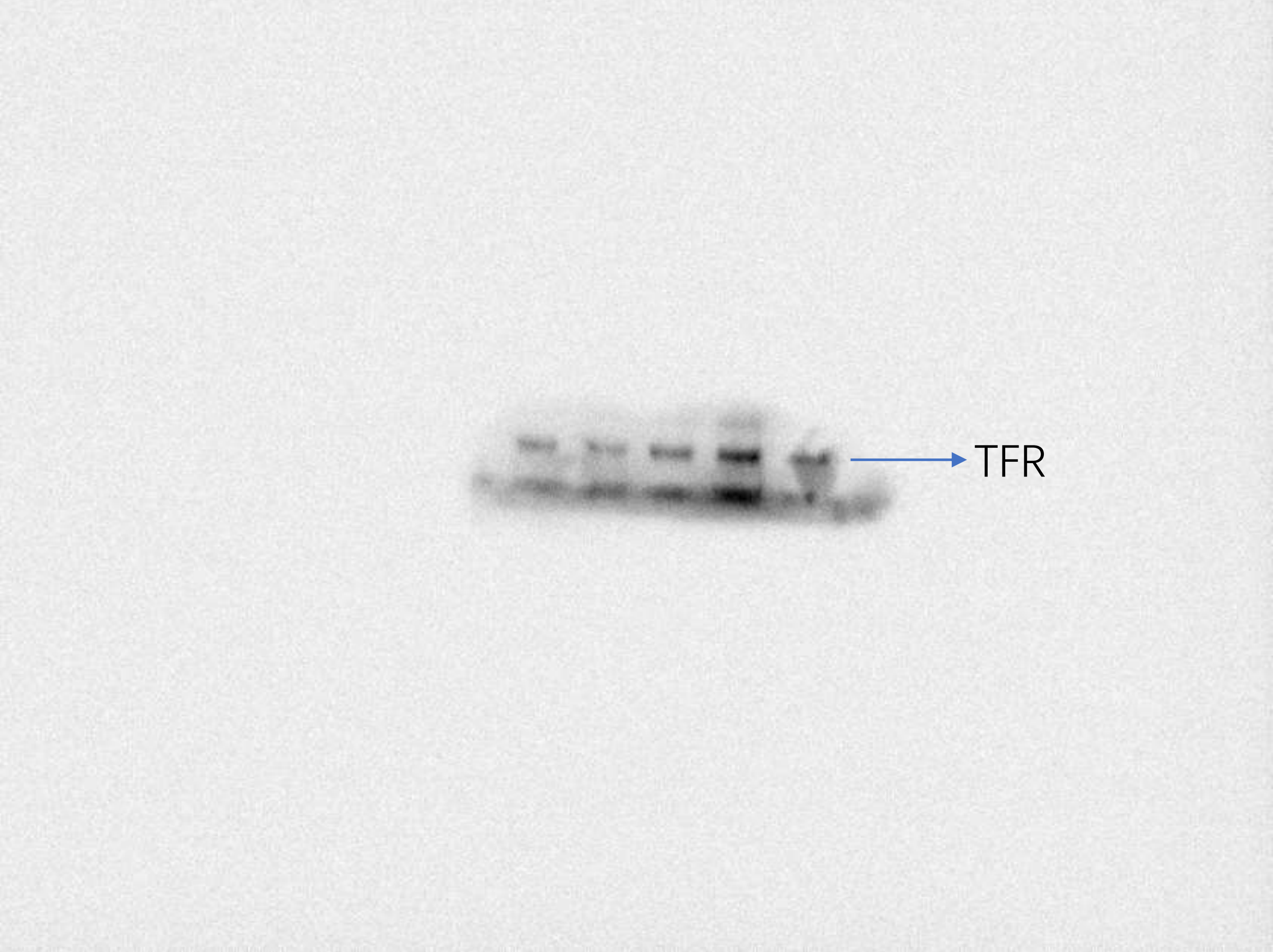

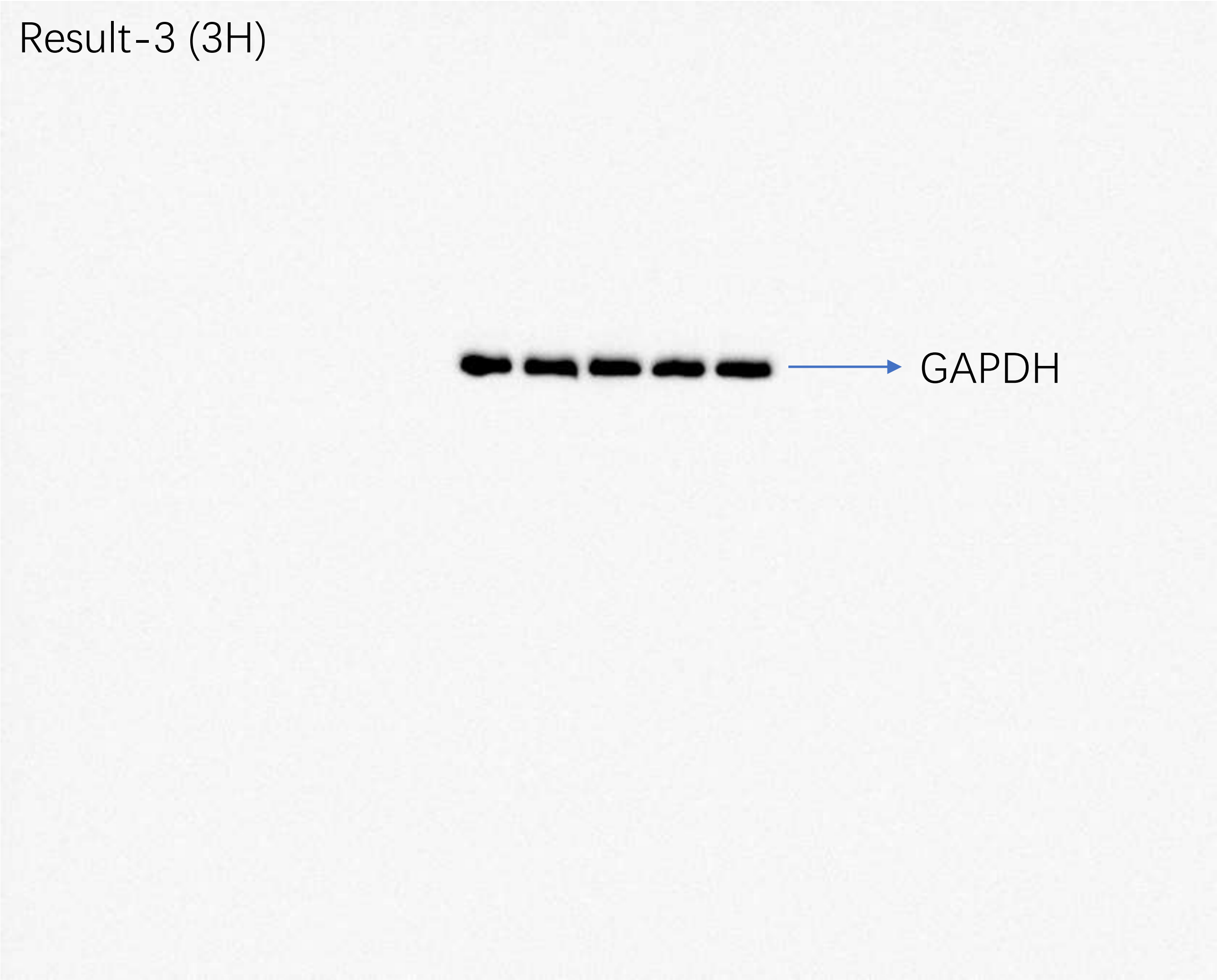
Result-3 (3H)
GAPDH

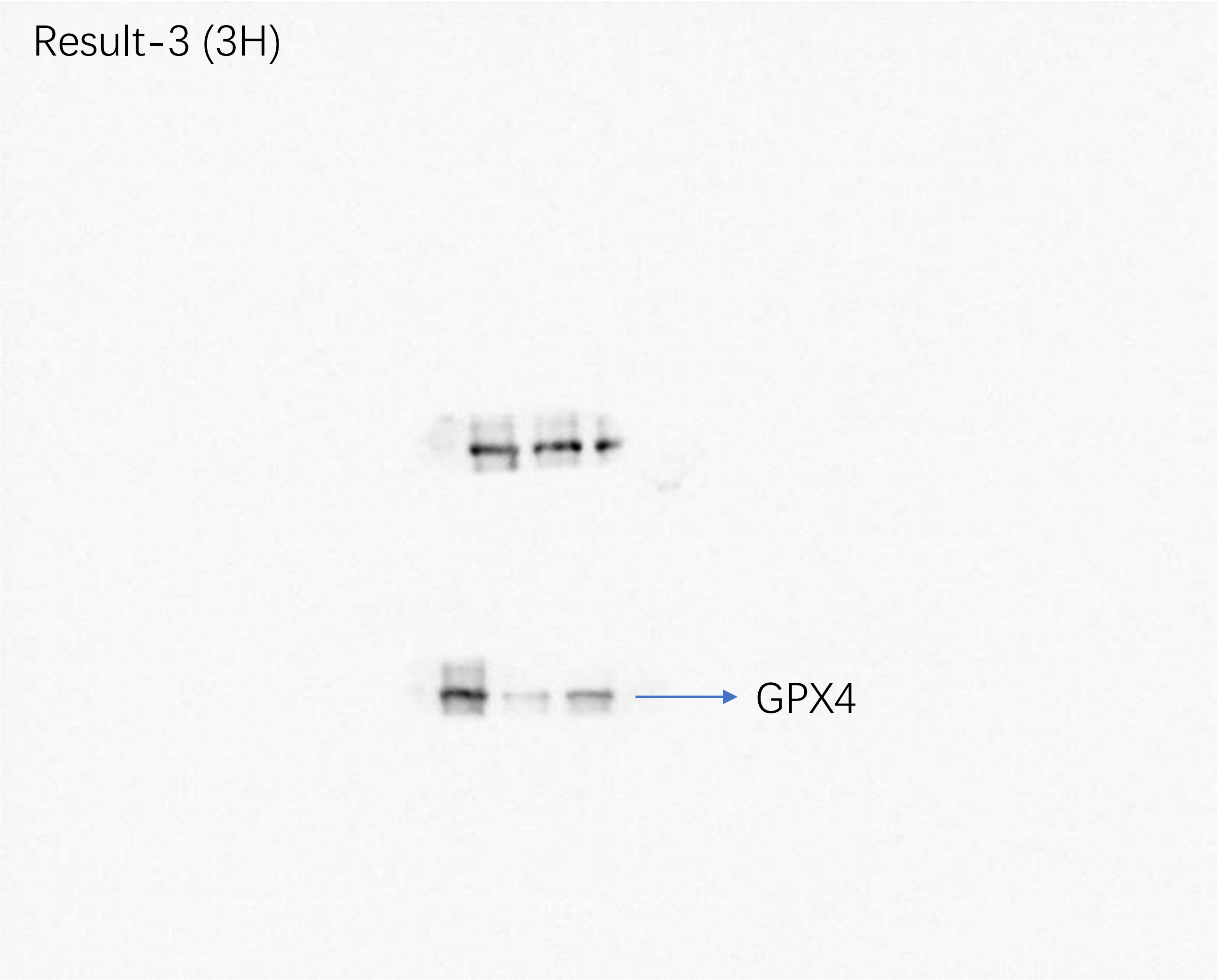
Result-3 (3H)
GPX4

Result-3 (3H)

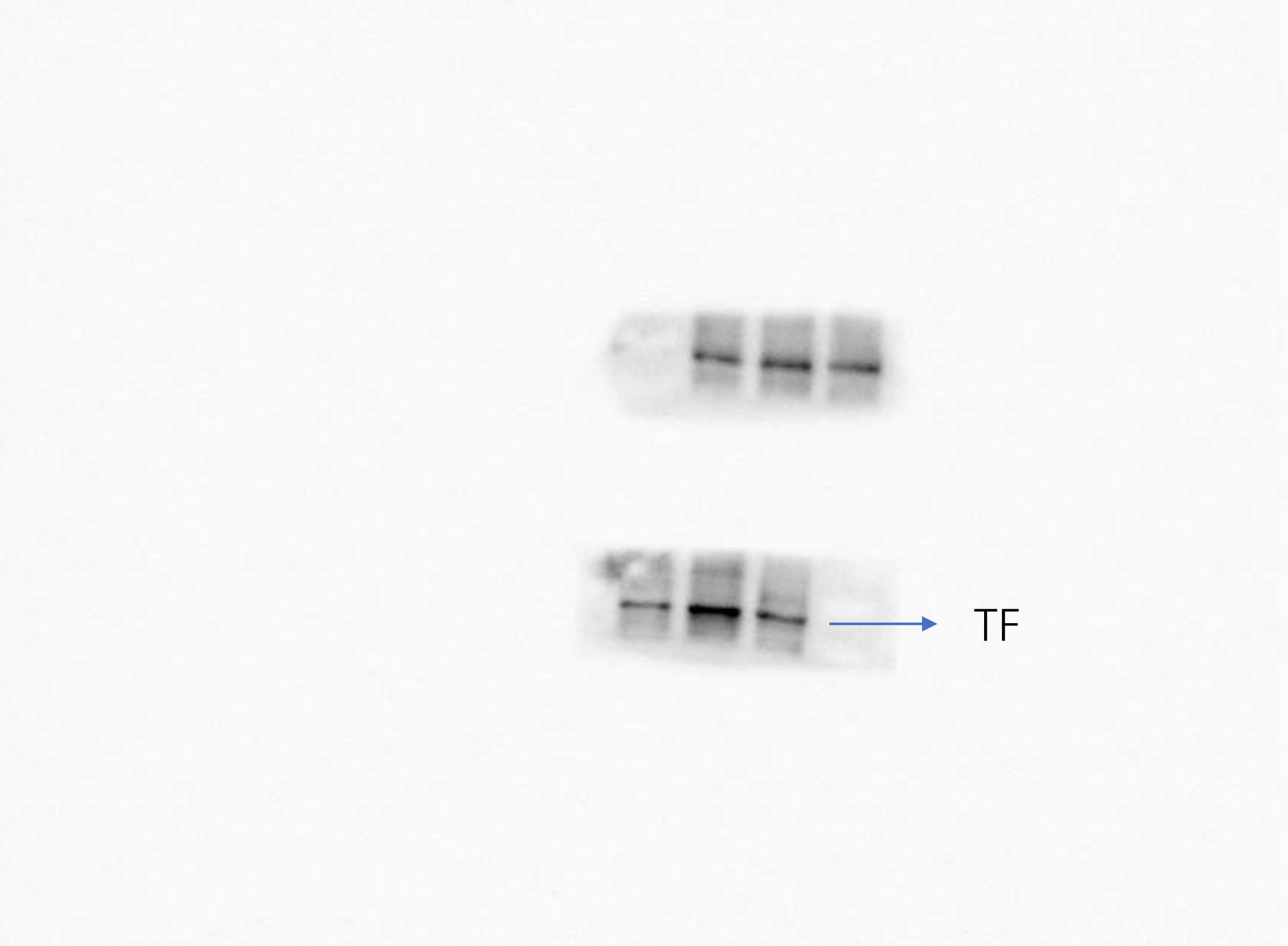

Result-3 (3H)

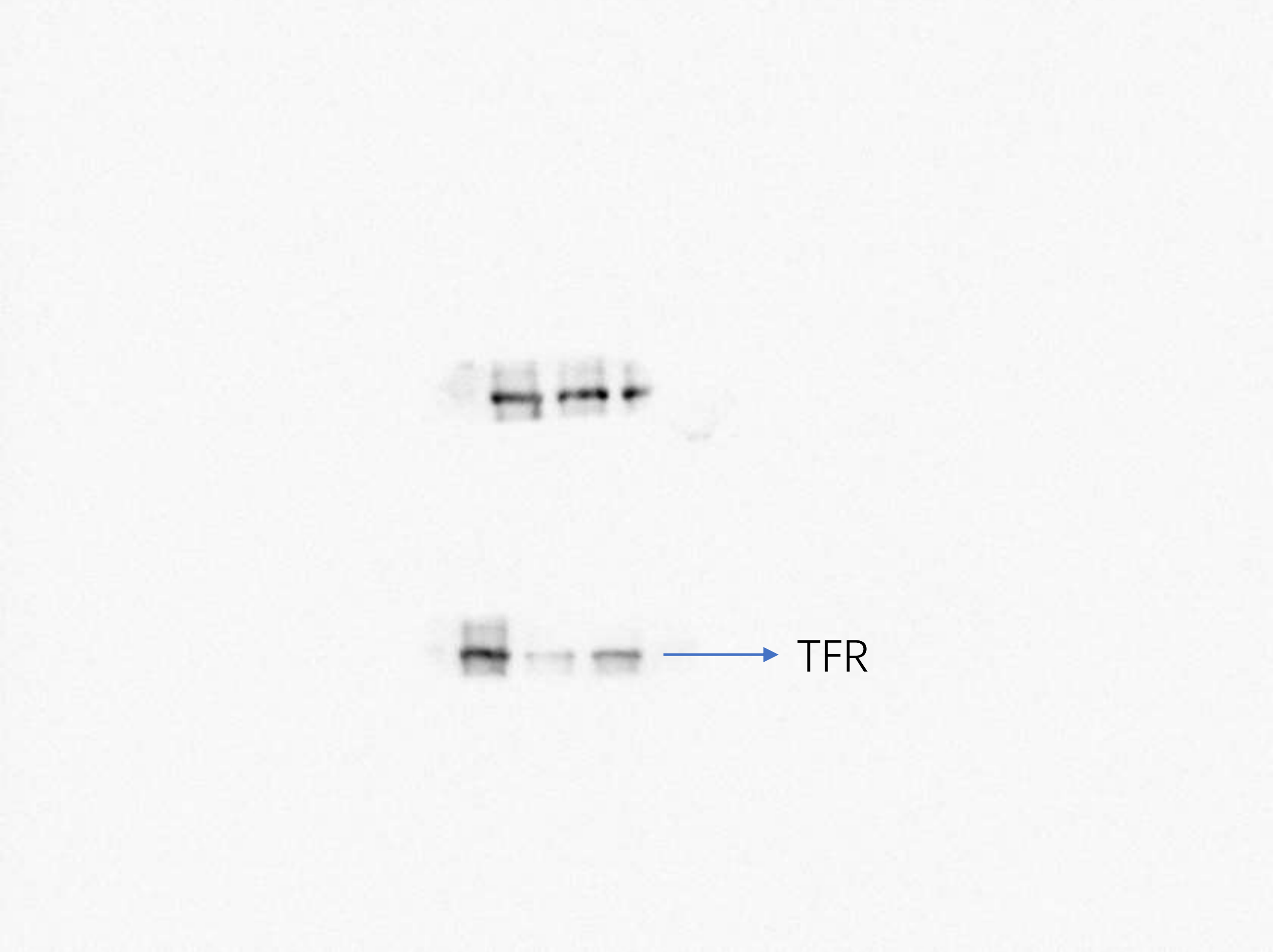

Result-3 (3H)

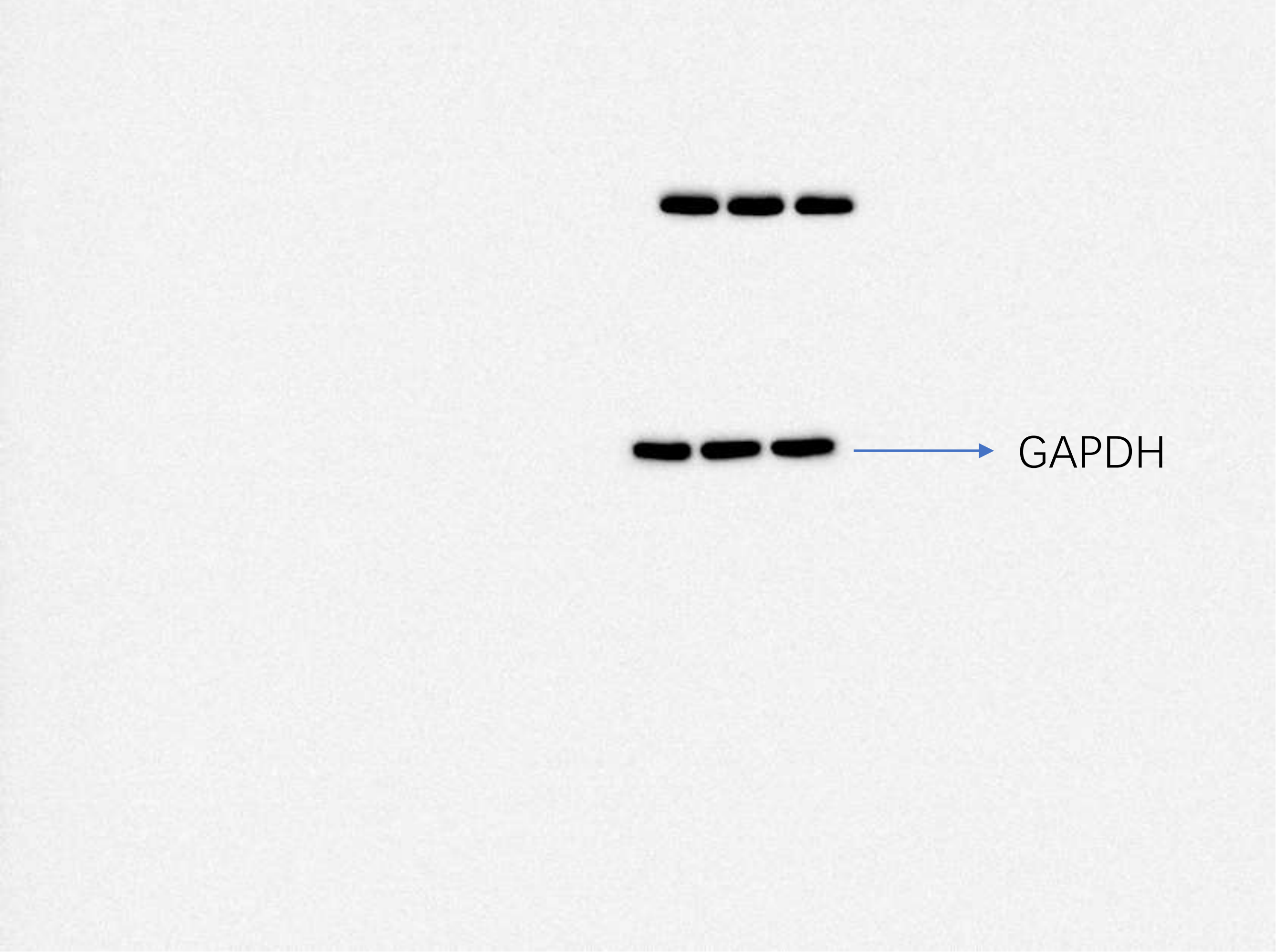

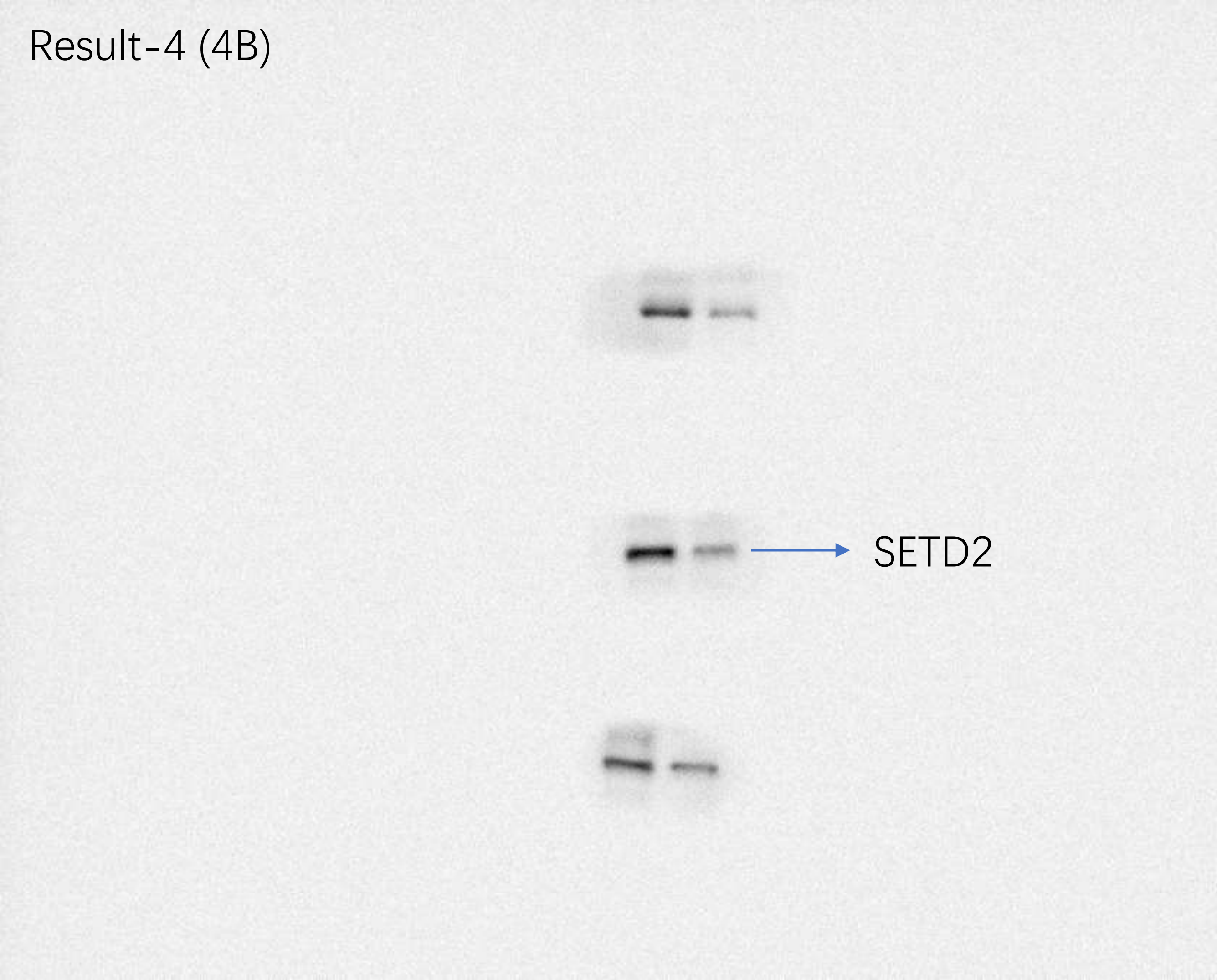
Result-4 (4B)
SETD2

Result-4 (4B)

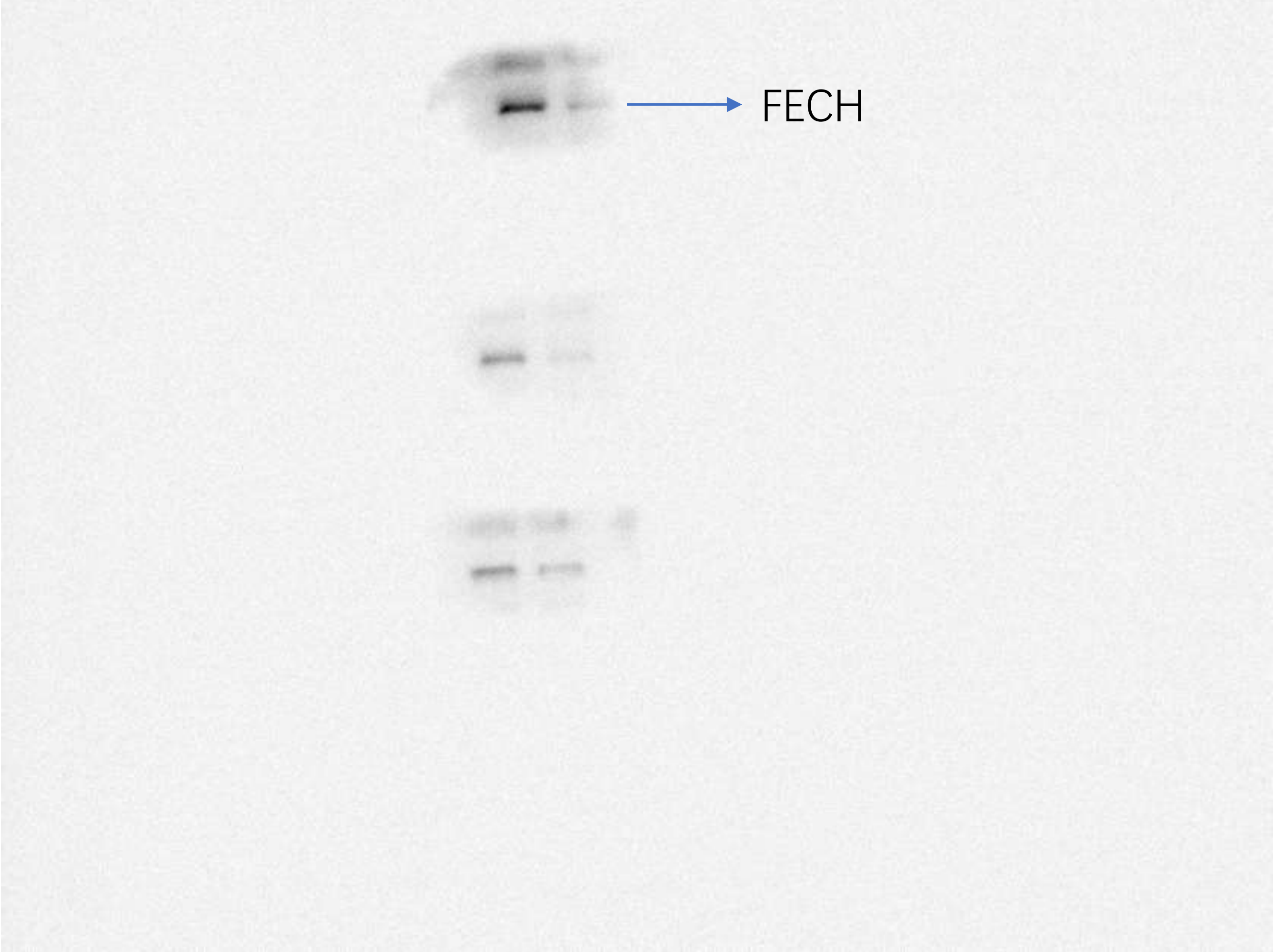

Result-4 (4B)

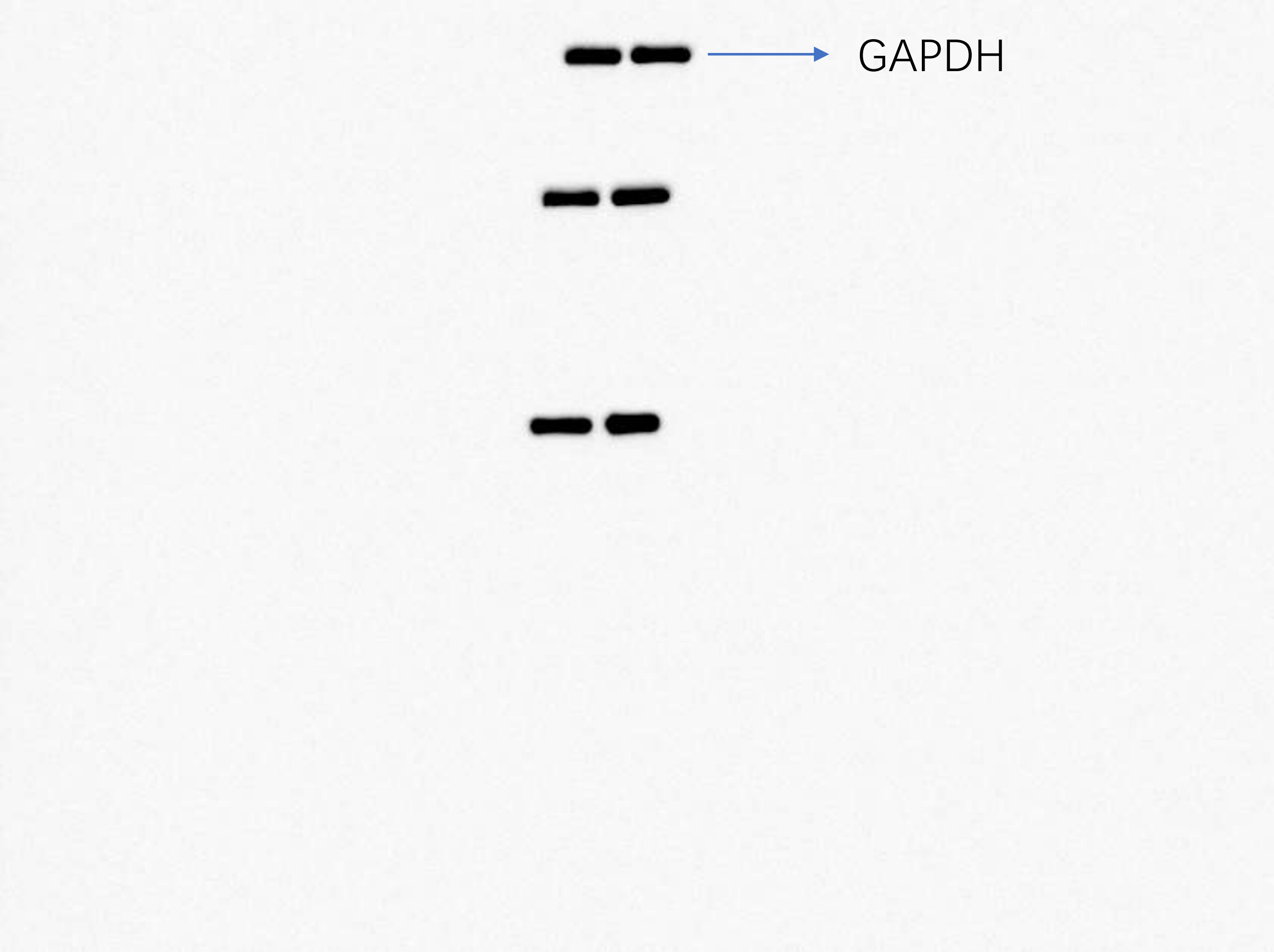

Result-4 (4B)

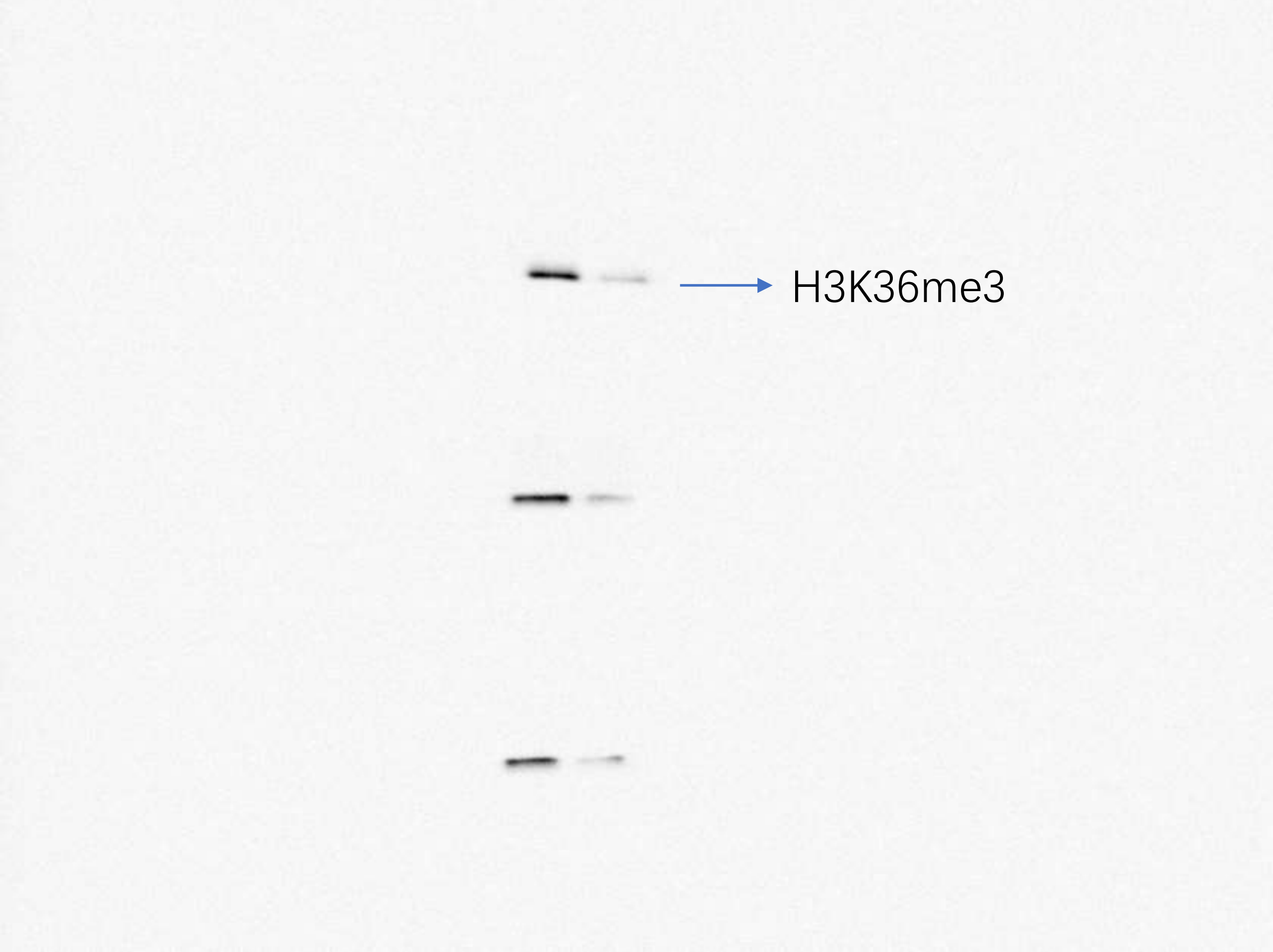

Result-4 (4B)

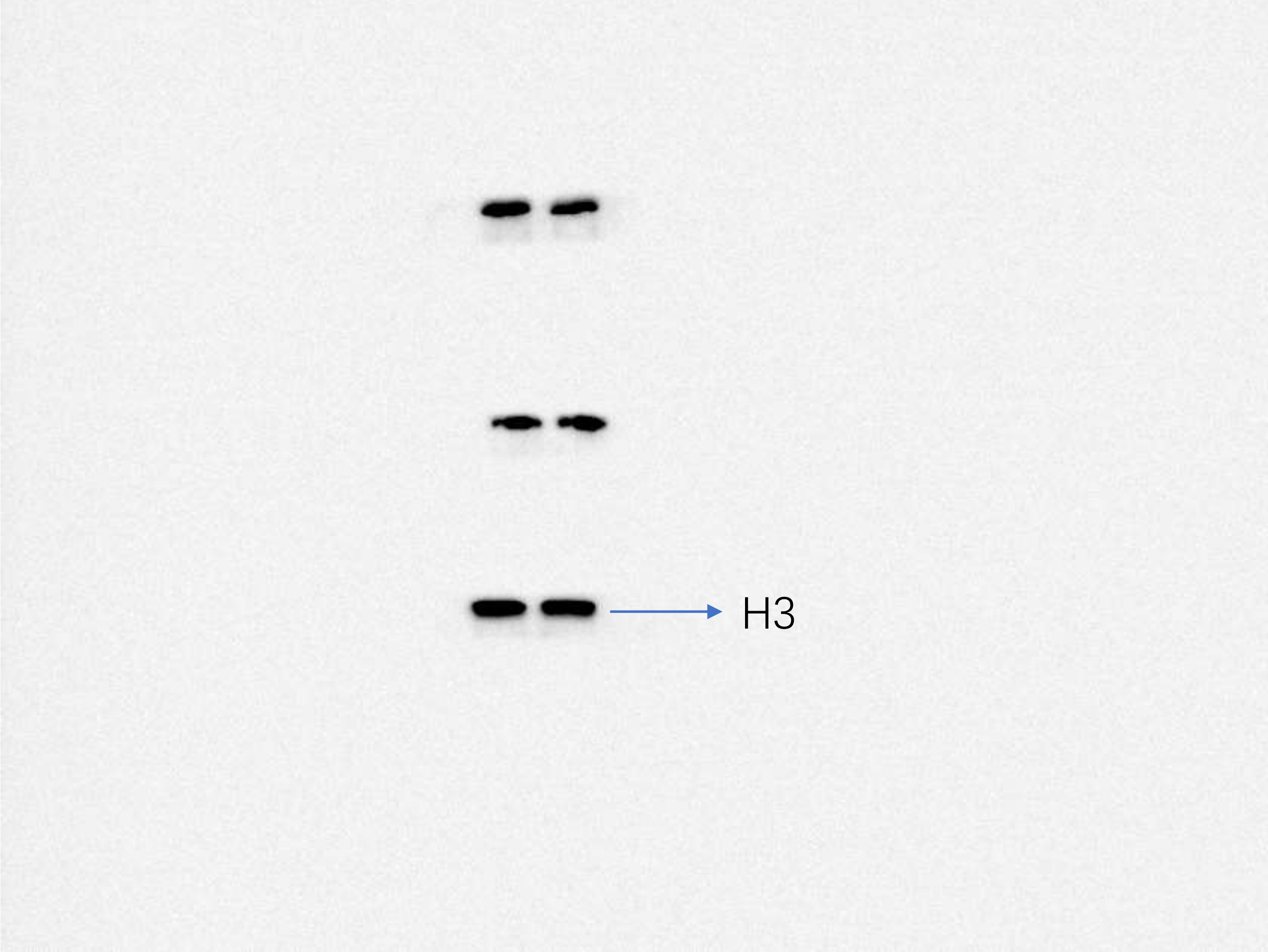

Result-4 (4B)

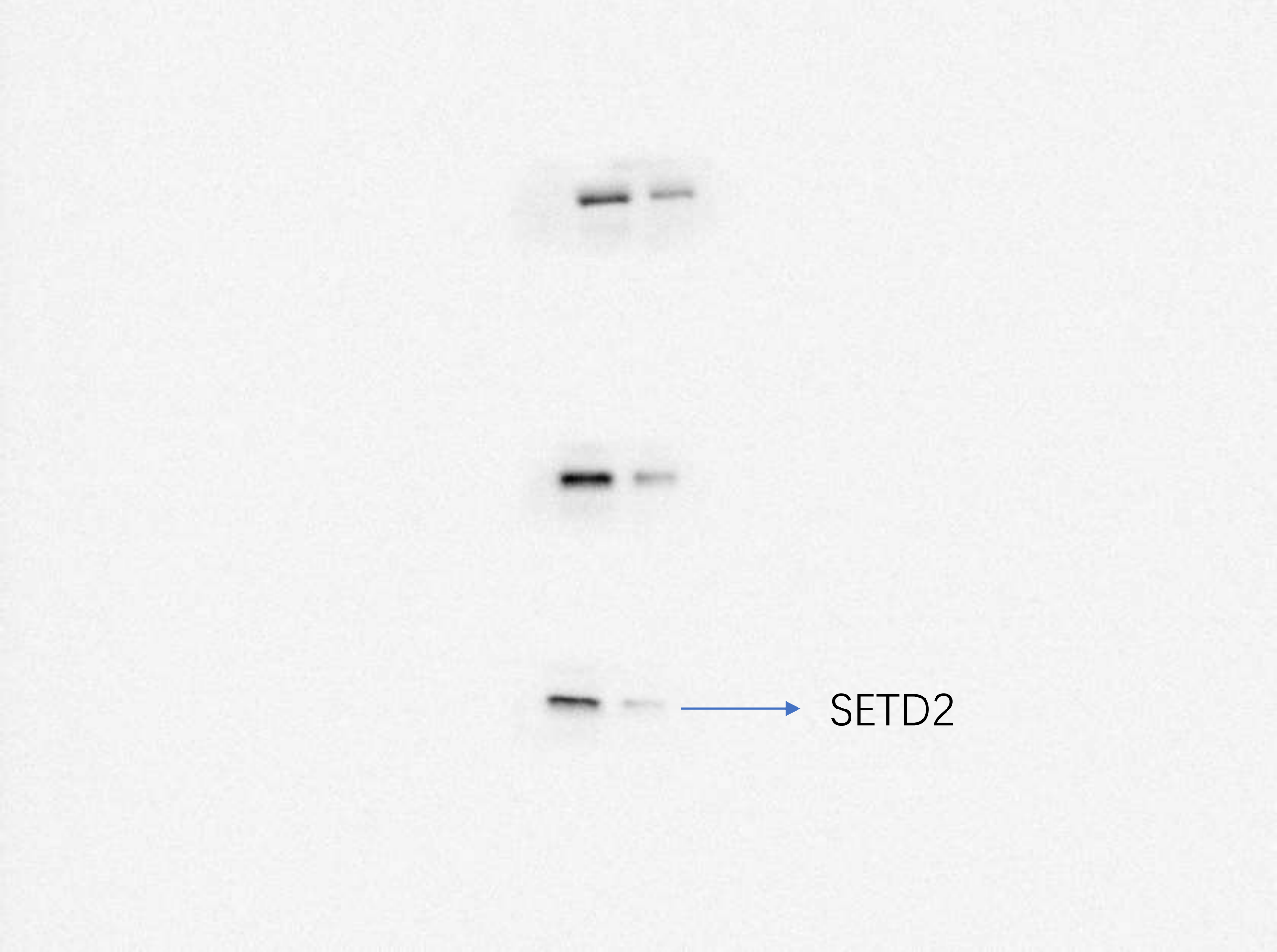

Result-4 (4B)

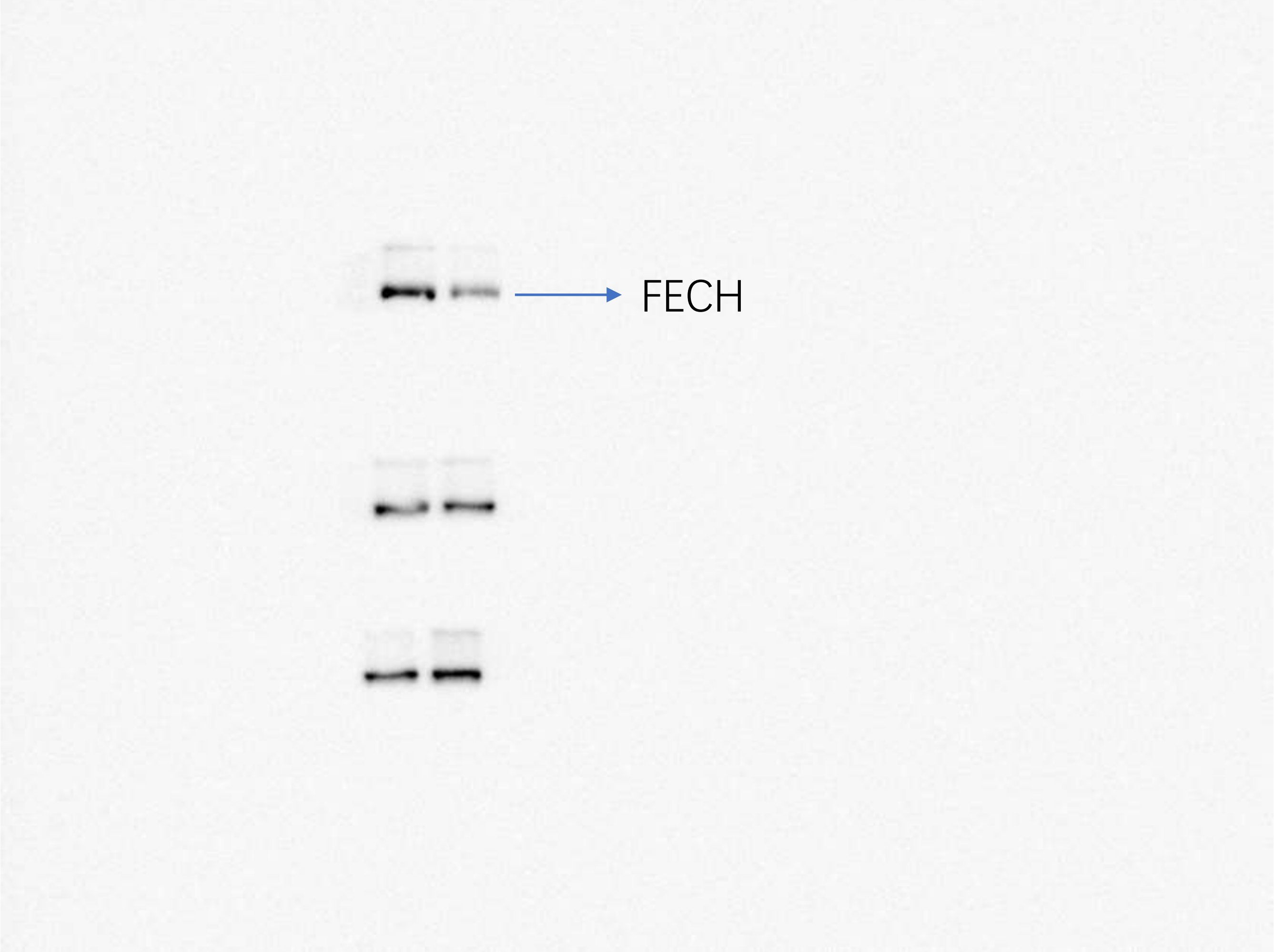

Result-4 (4B)

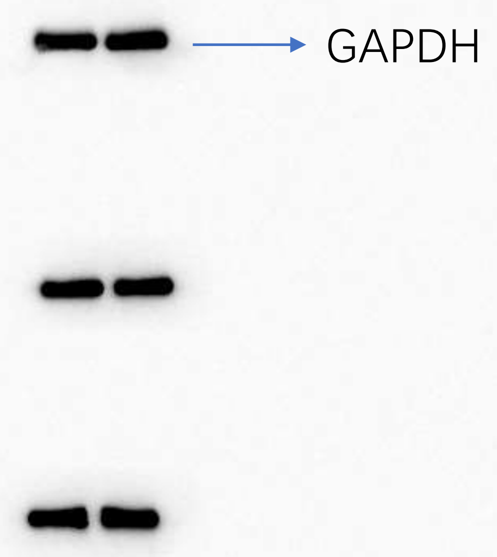

Result-4 (4B)

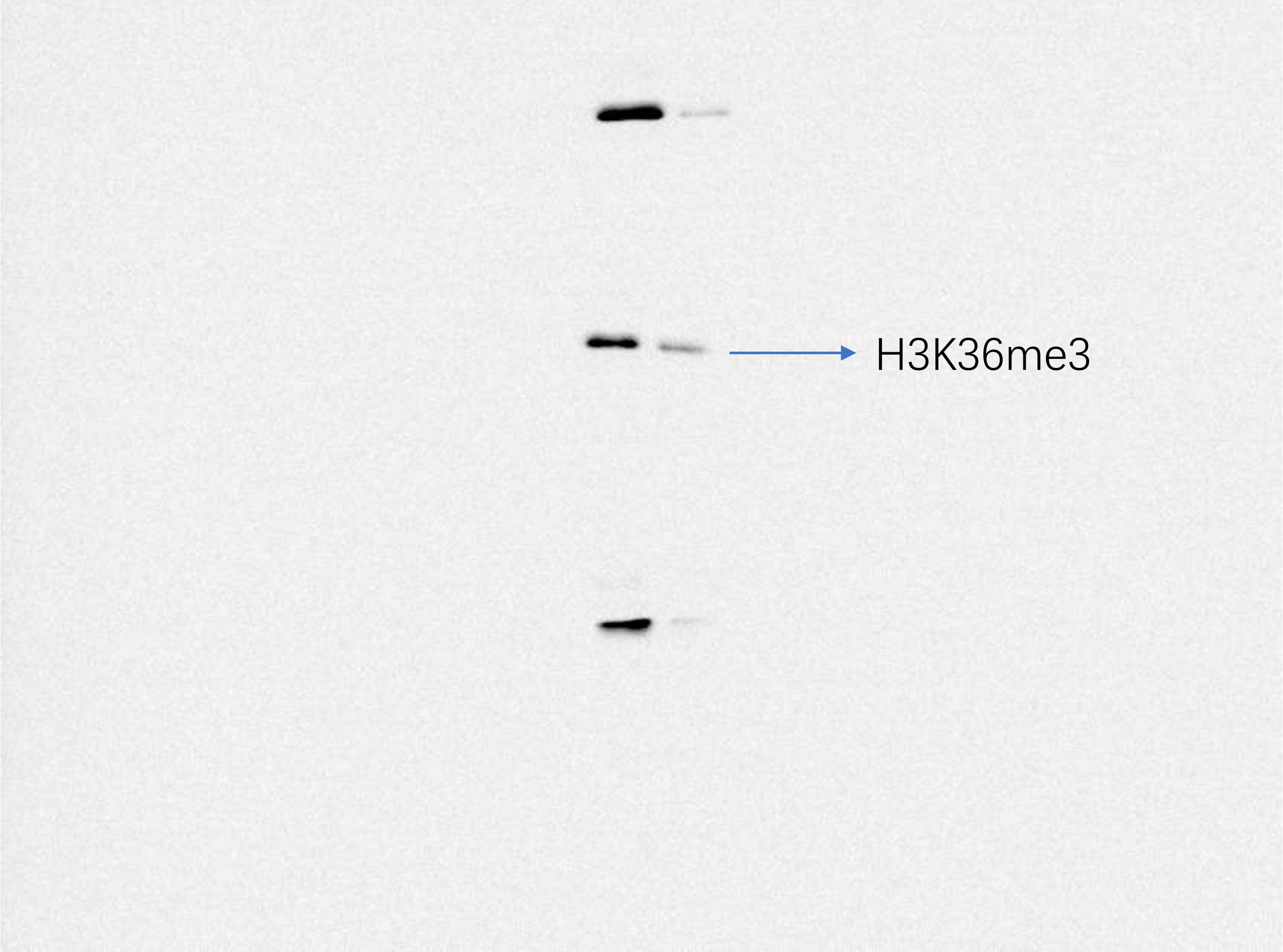

Result-4 (4B)

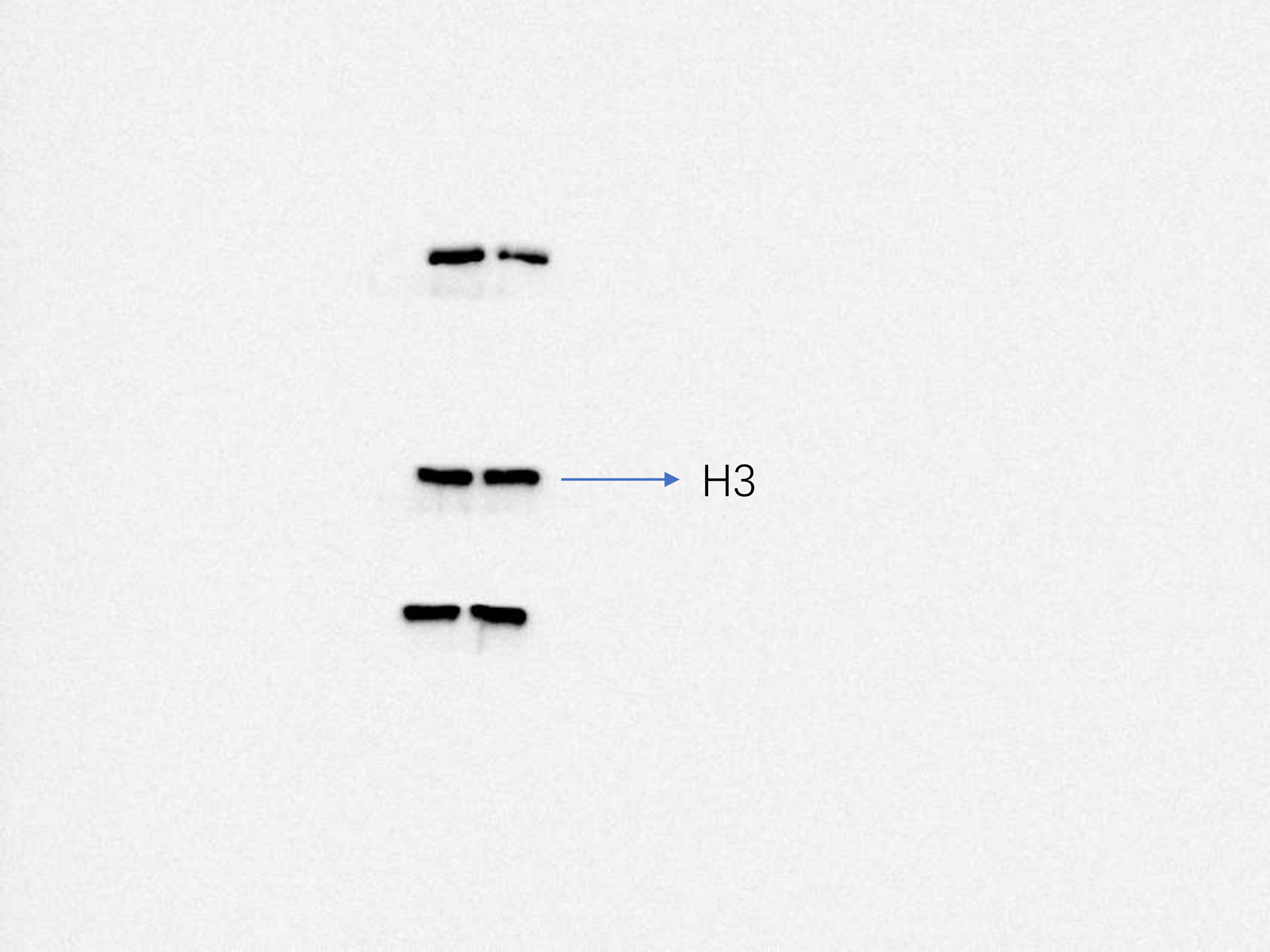

Result-4 (4C)

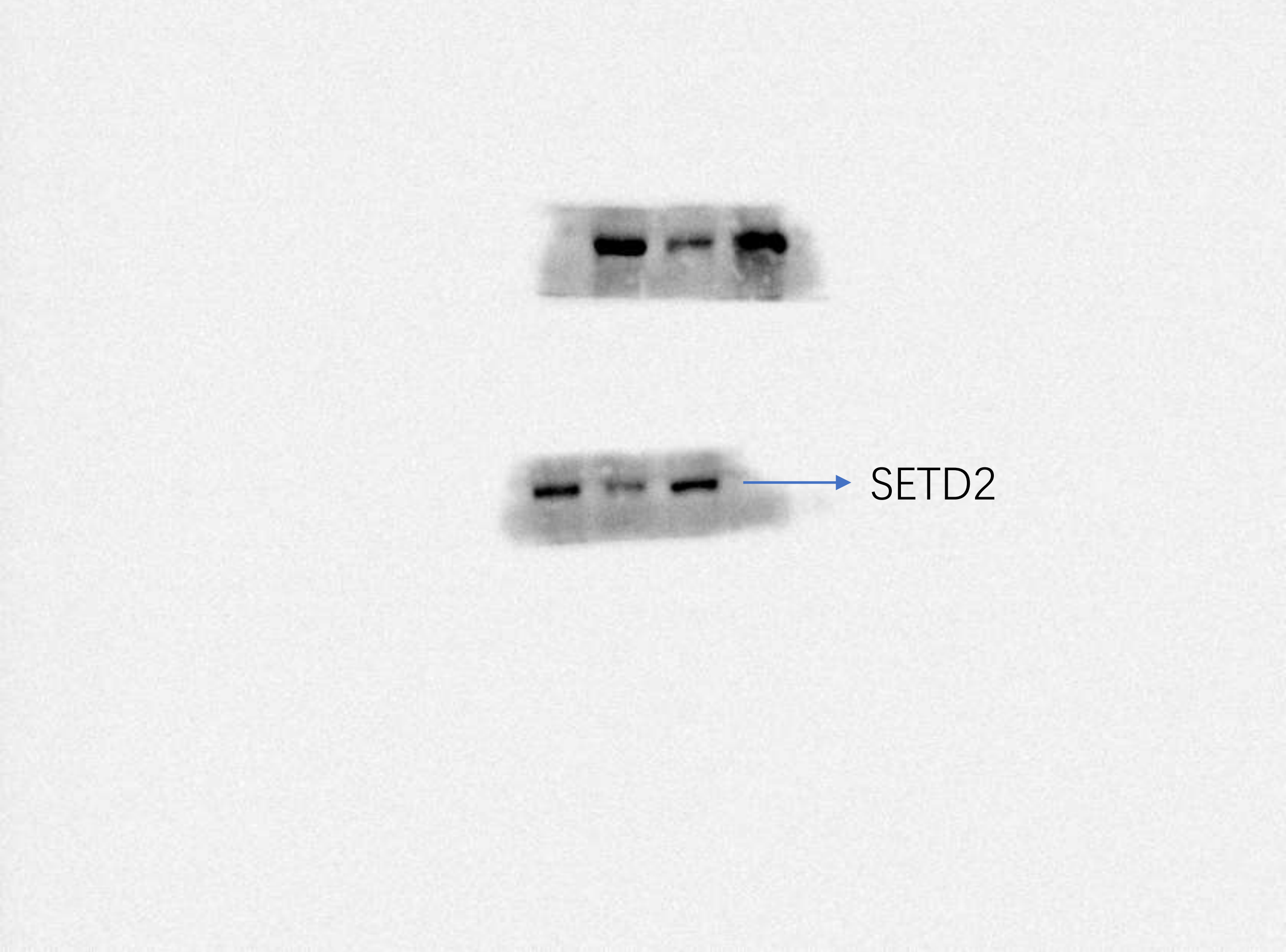

Result-4 (4C)

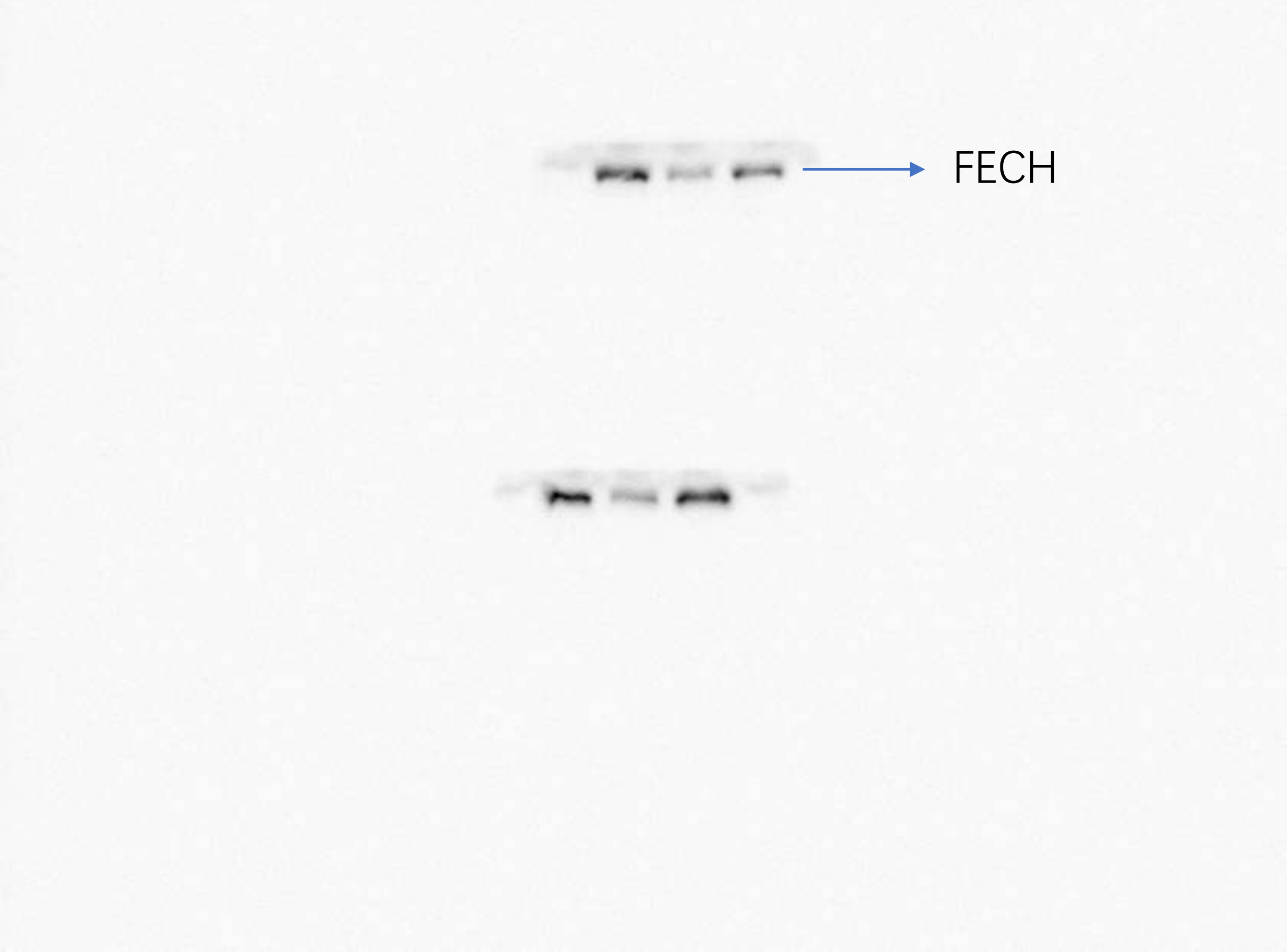

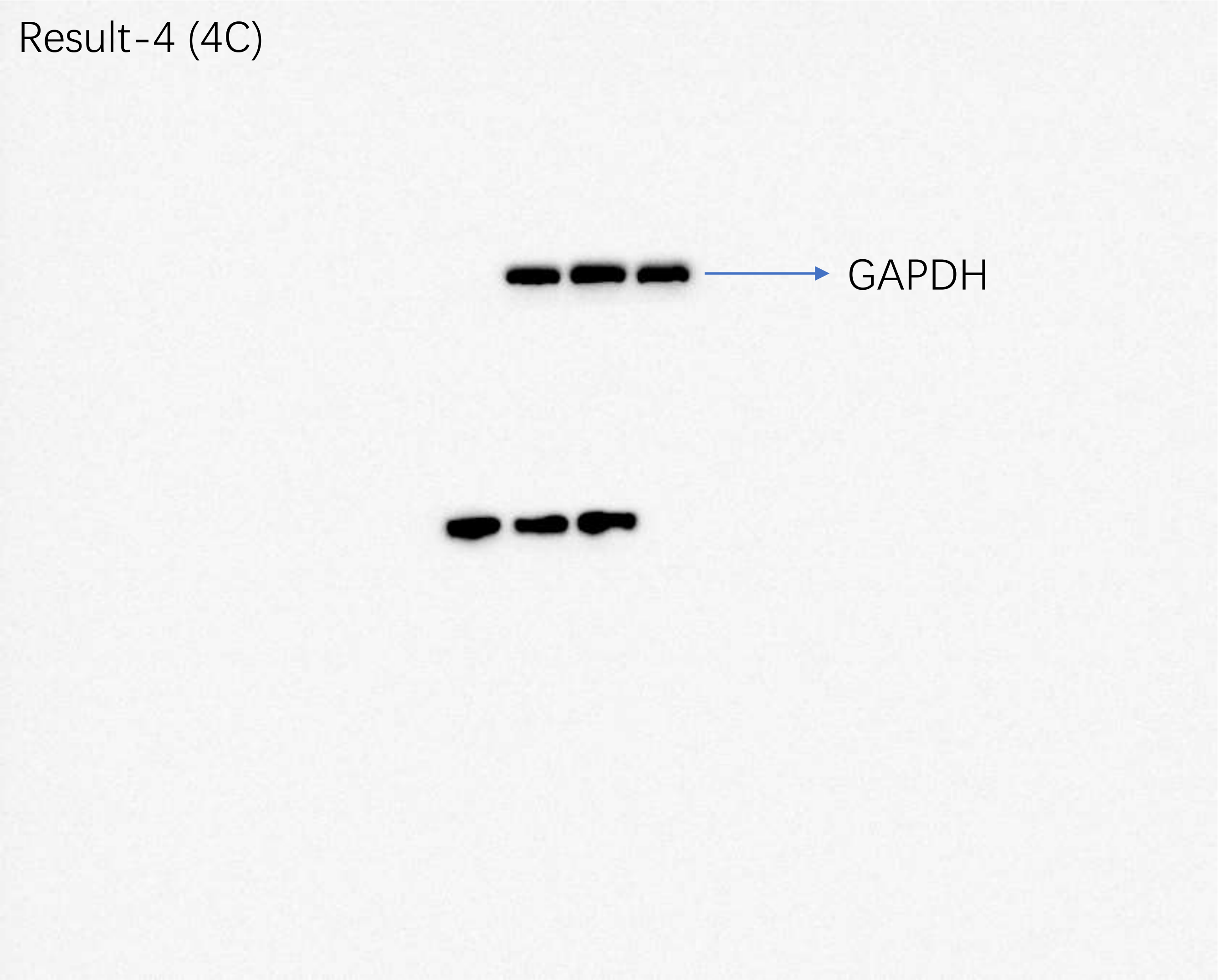
Result-4 (4C)
GAPDH

Result-4 (4C)

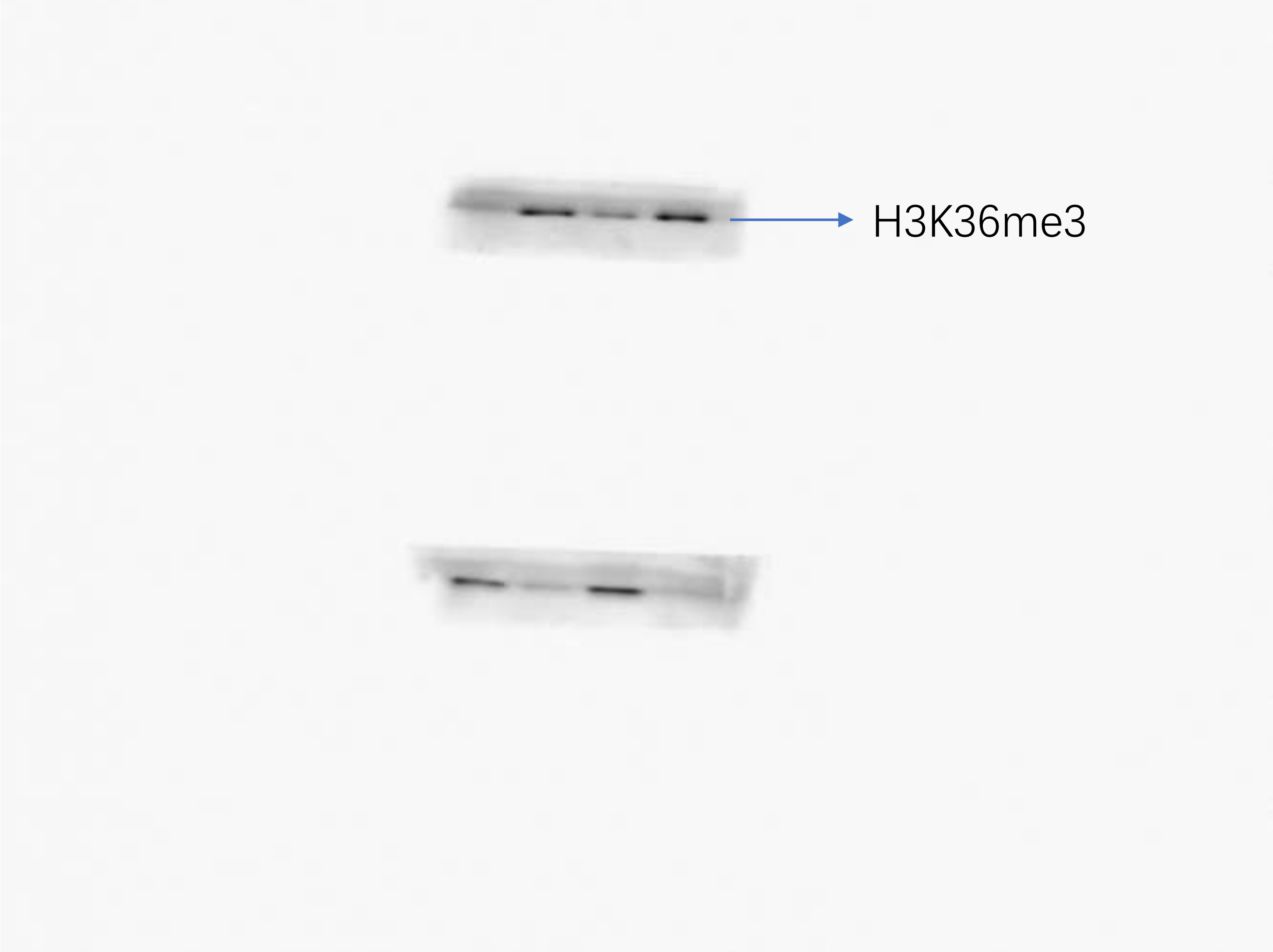

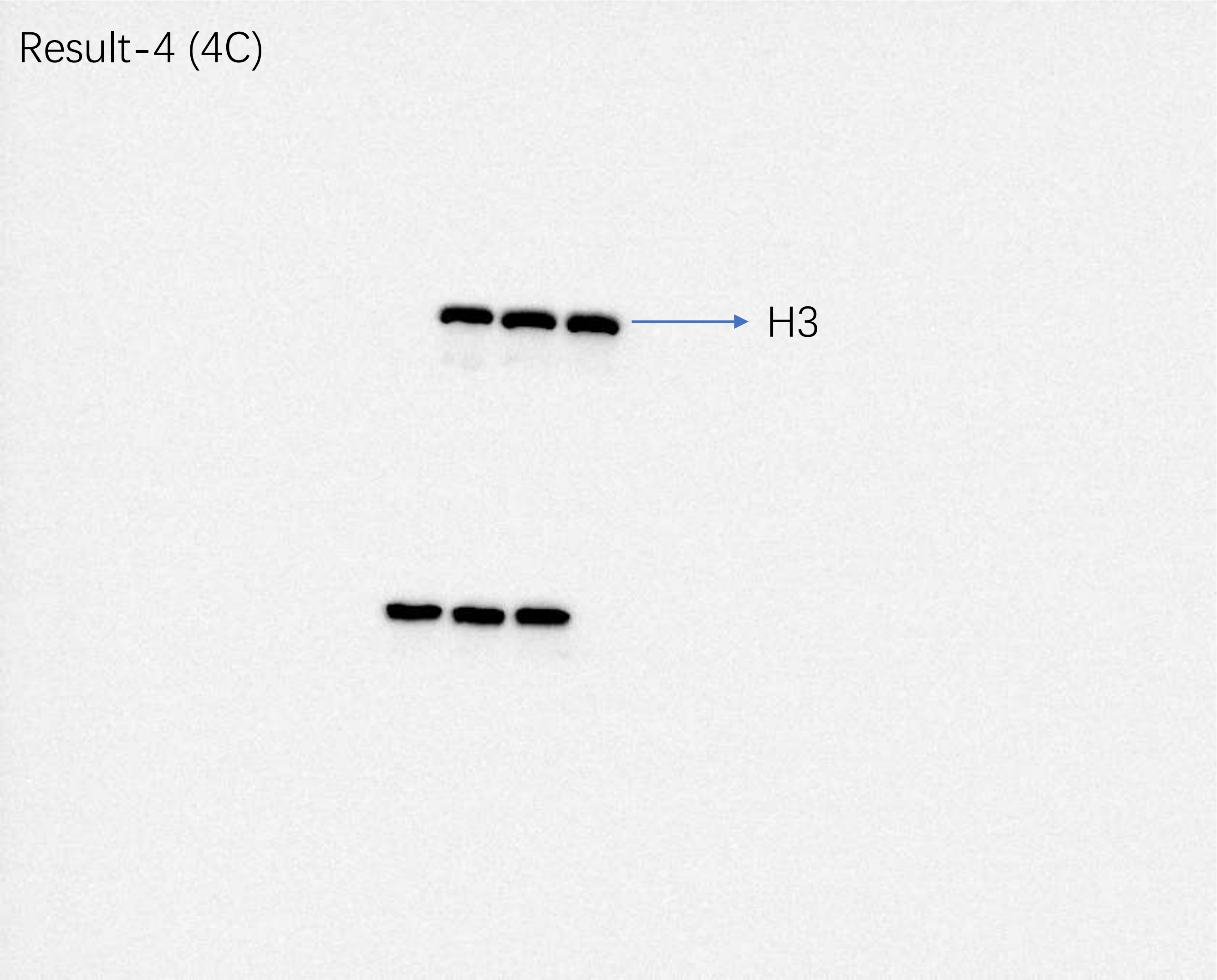
Result-4 (4C)
H3

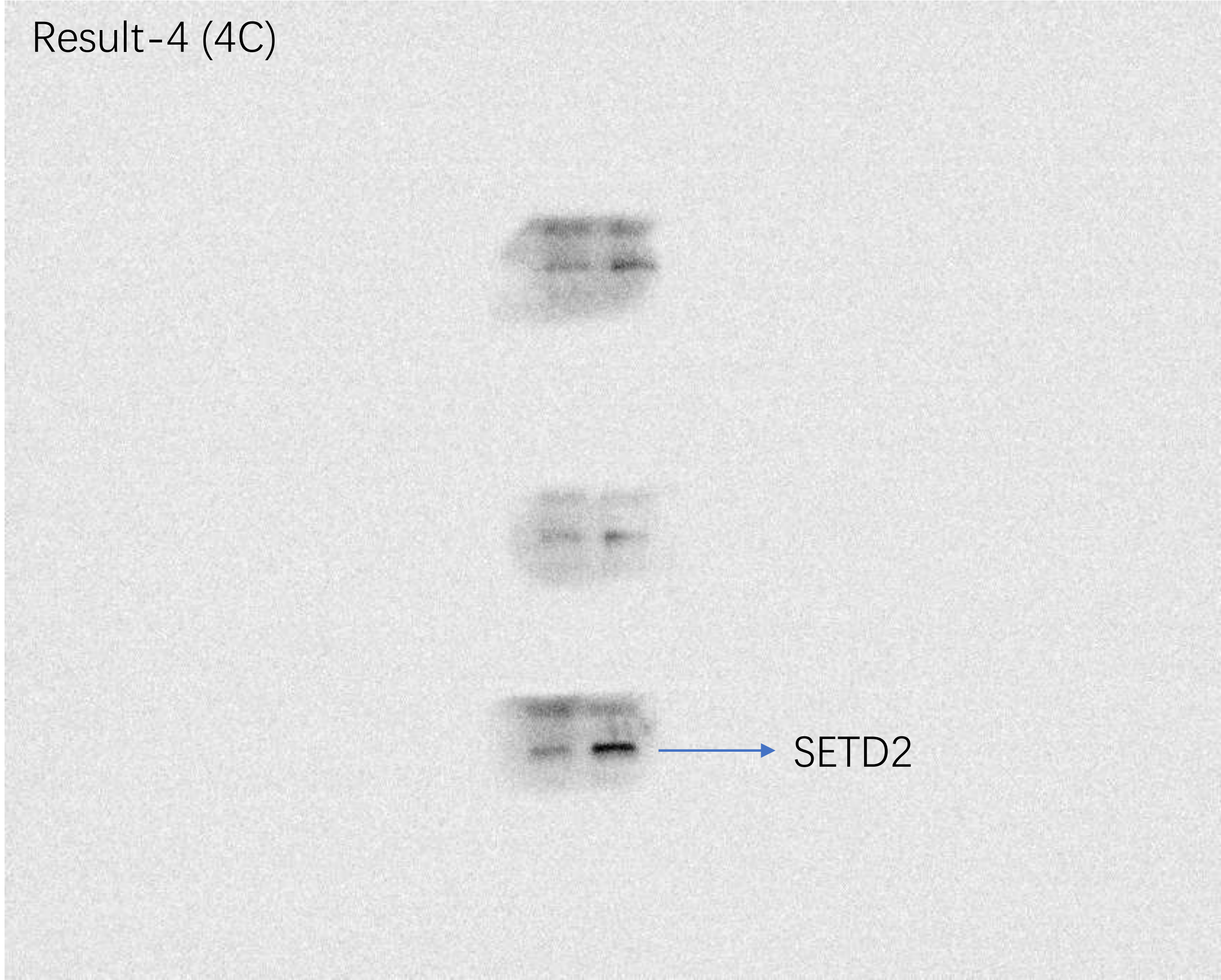
Result-4 (4C)
SETD2

Result-4 (4C)

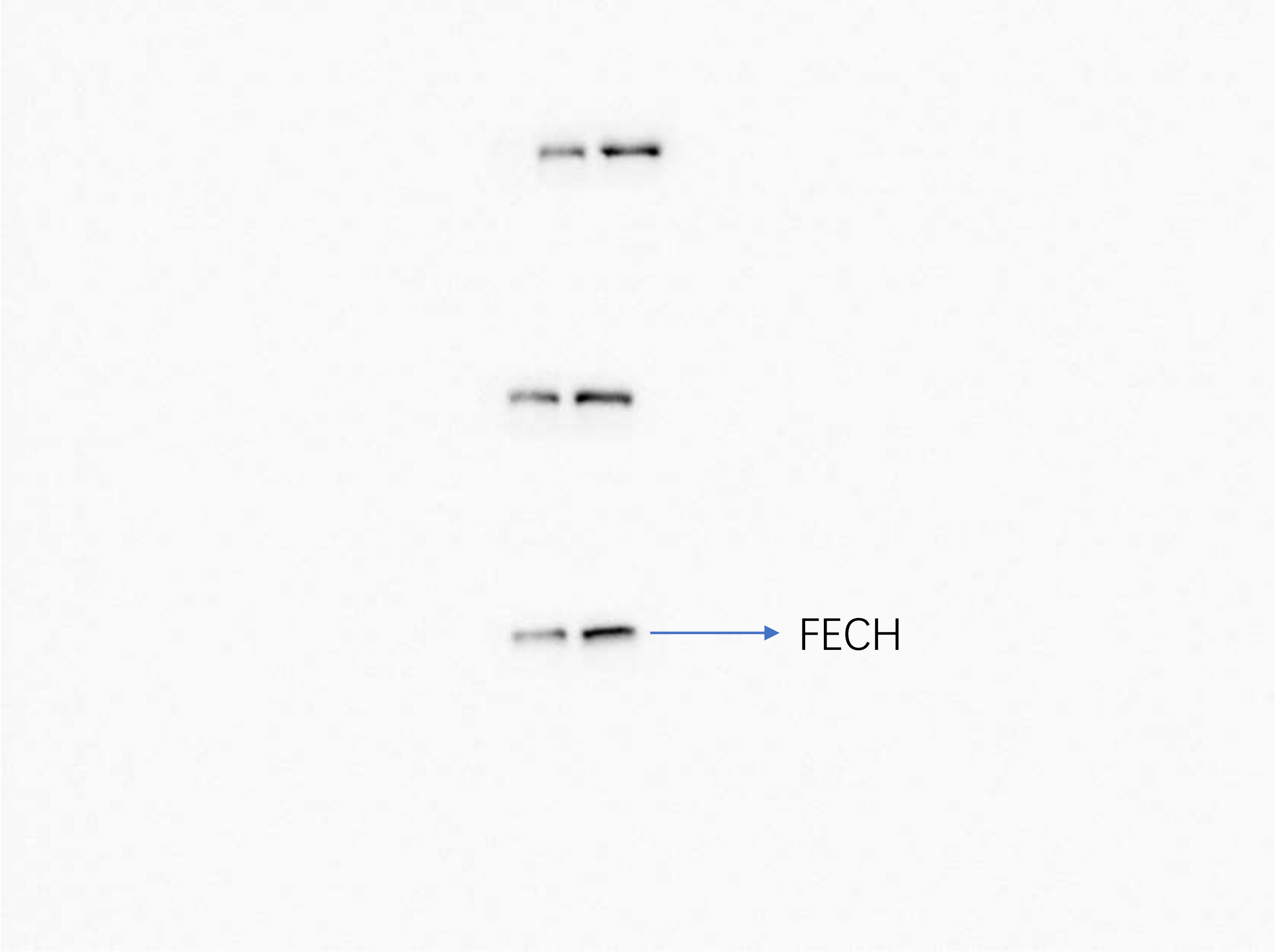

Result-4 (4C)

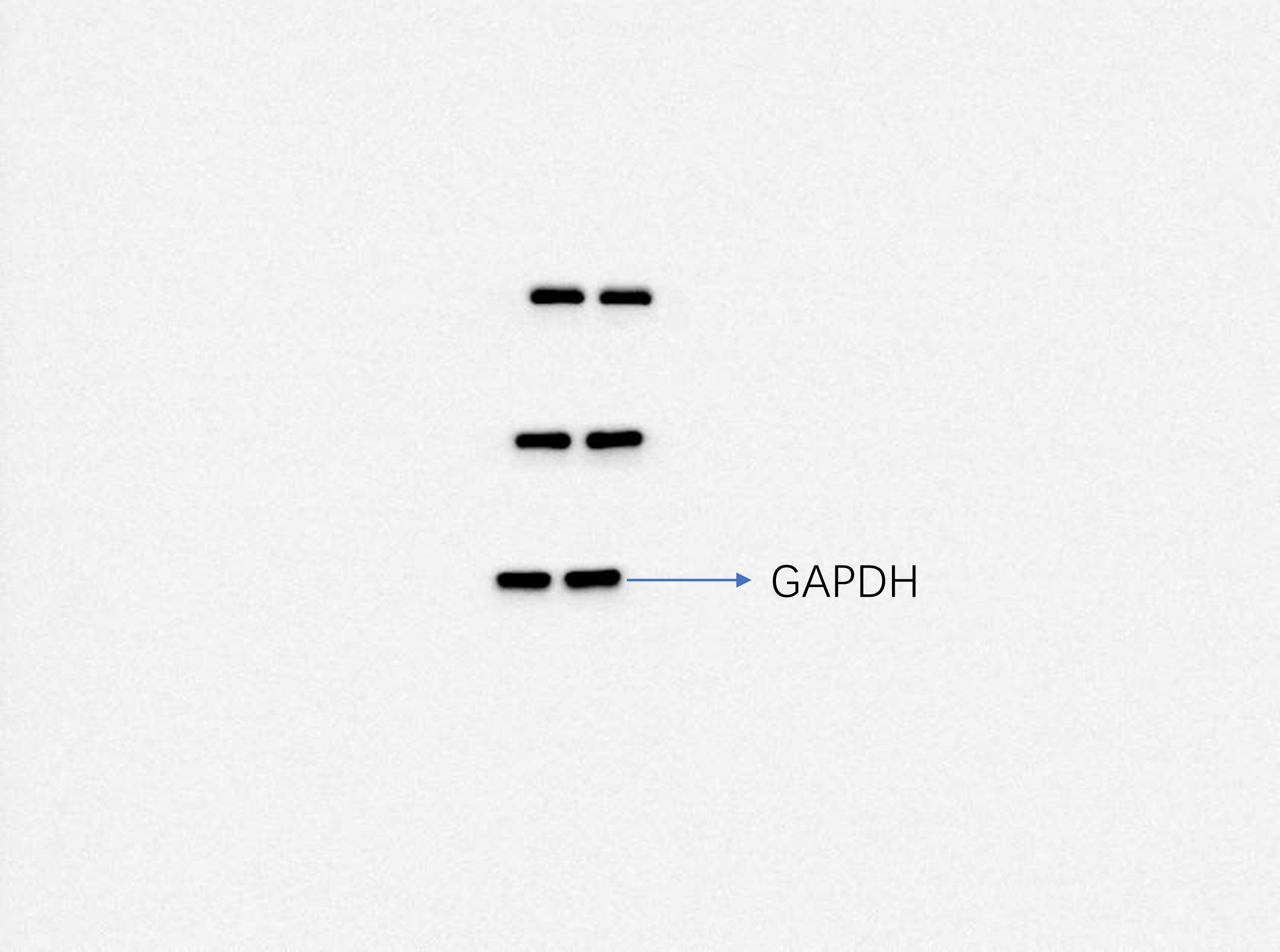

Result-4 (4C)

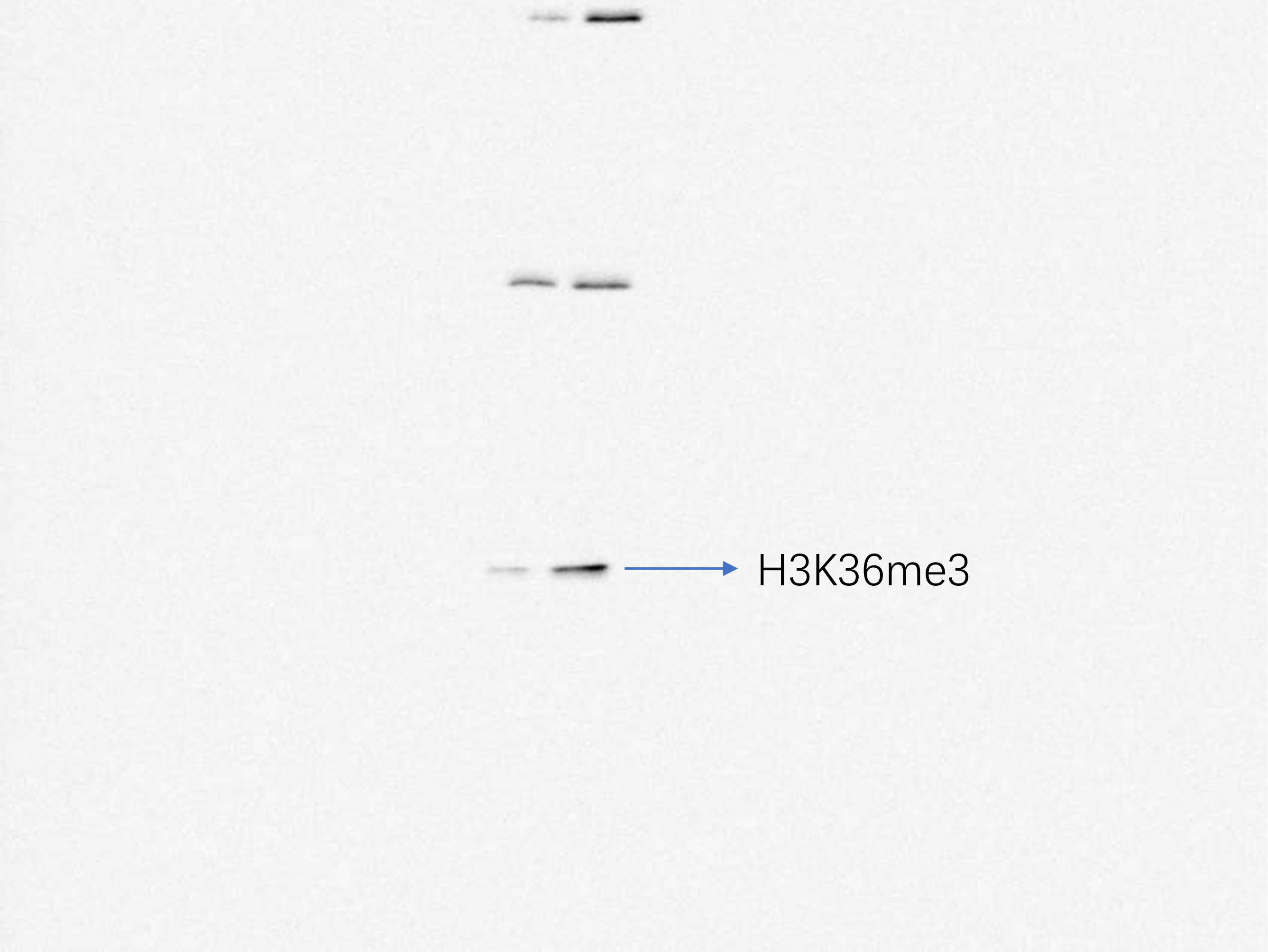

Result-4 (4C)

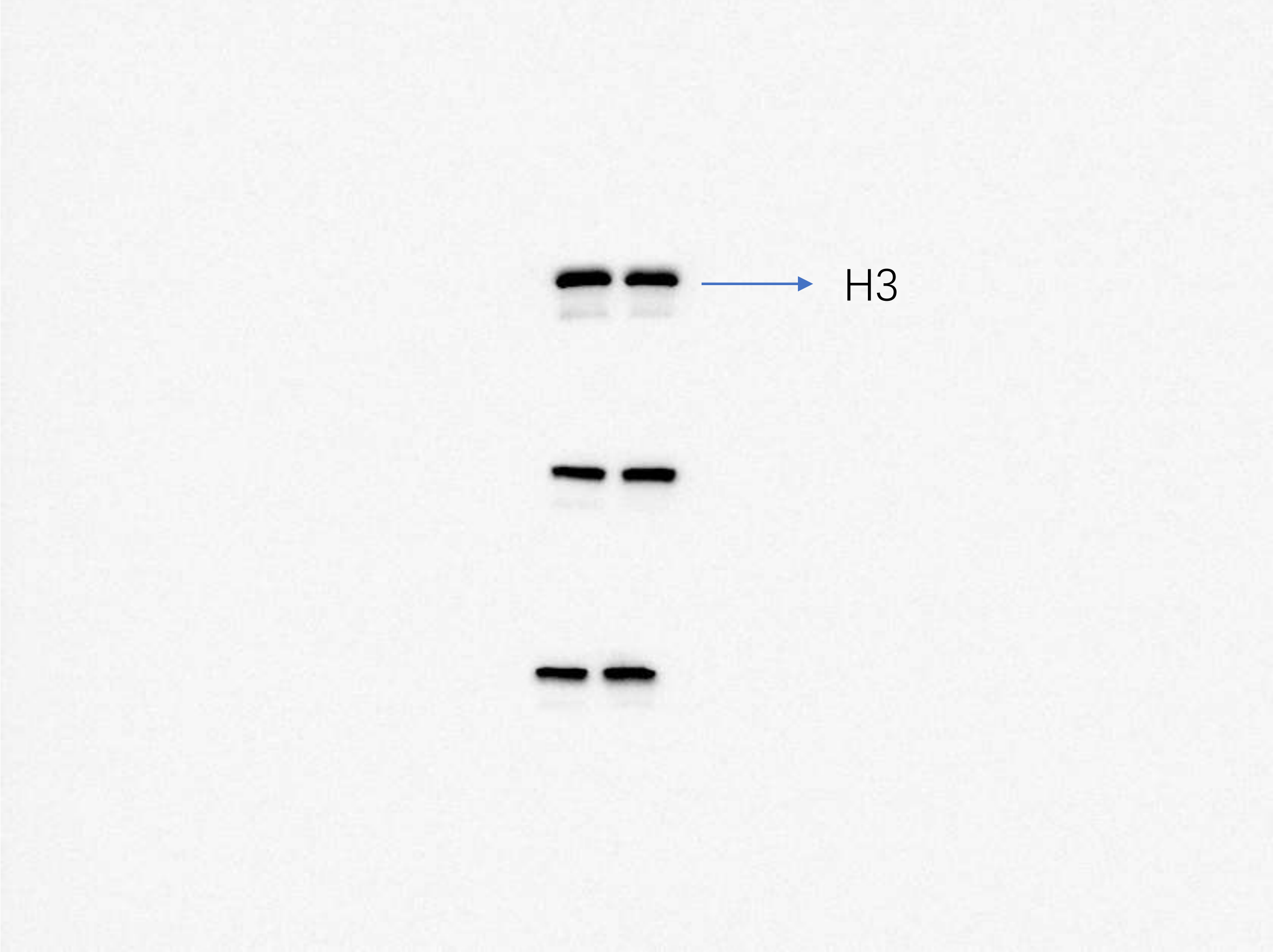

Result-4 (4H)

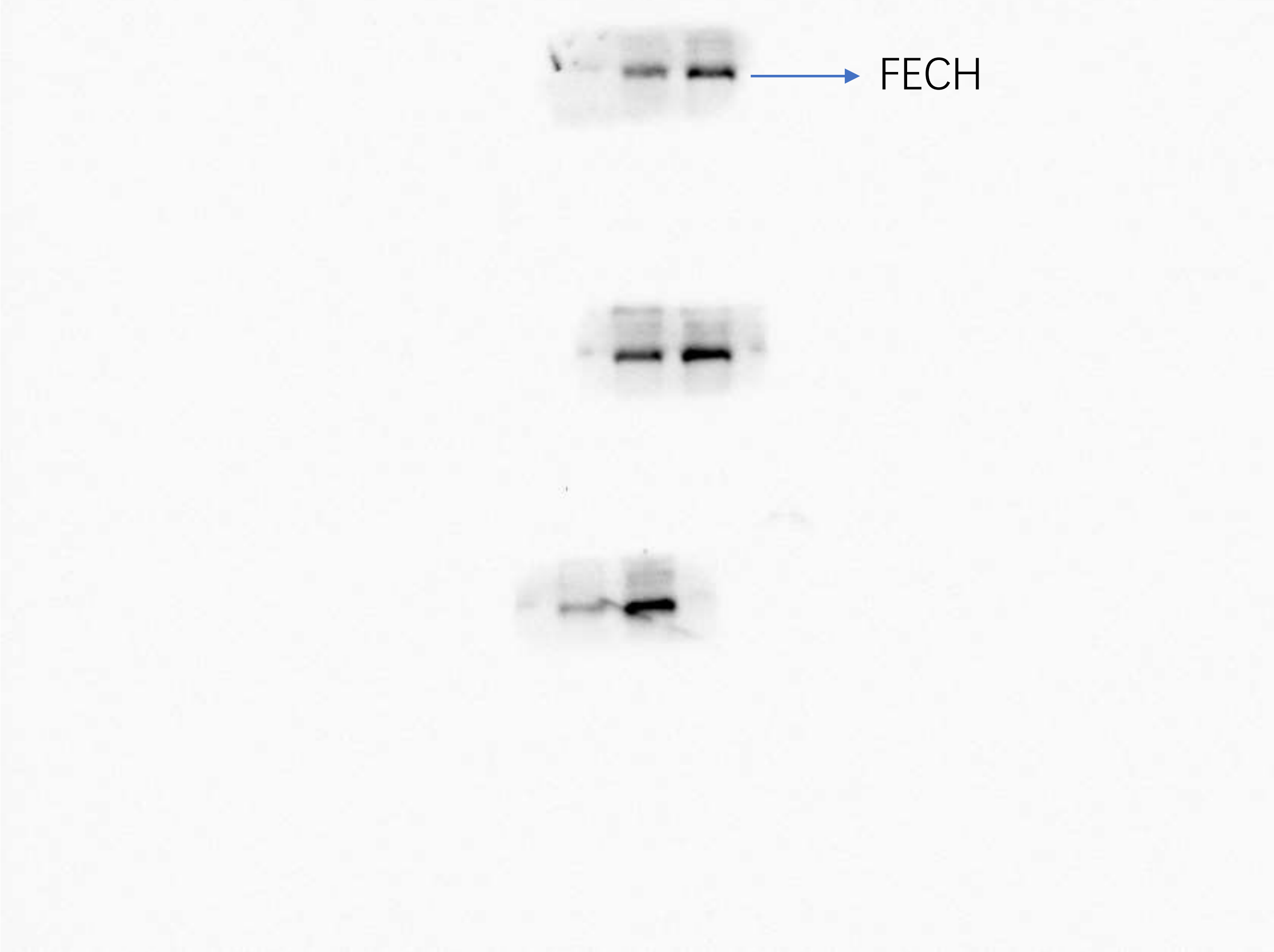

Result-4 (4H)

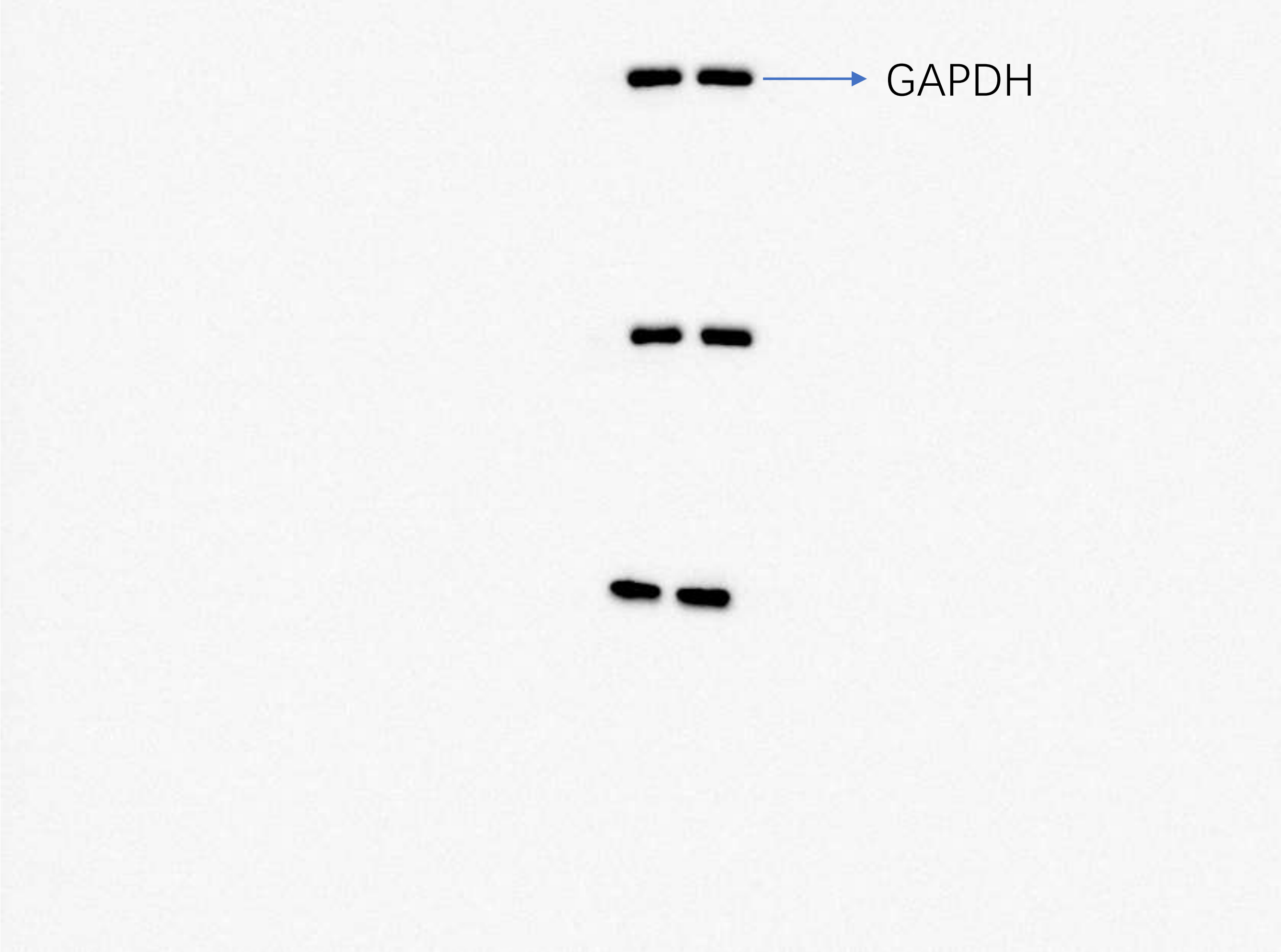

Result-4 (4H)

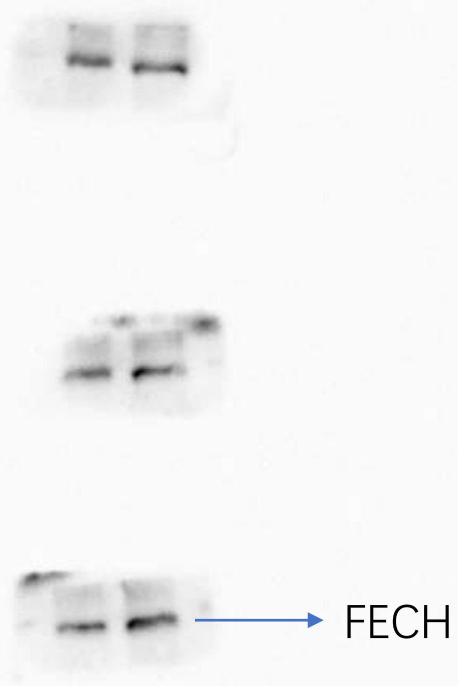

Result-4 (4H)

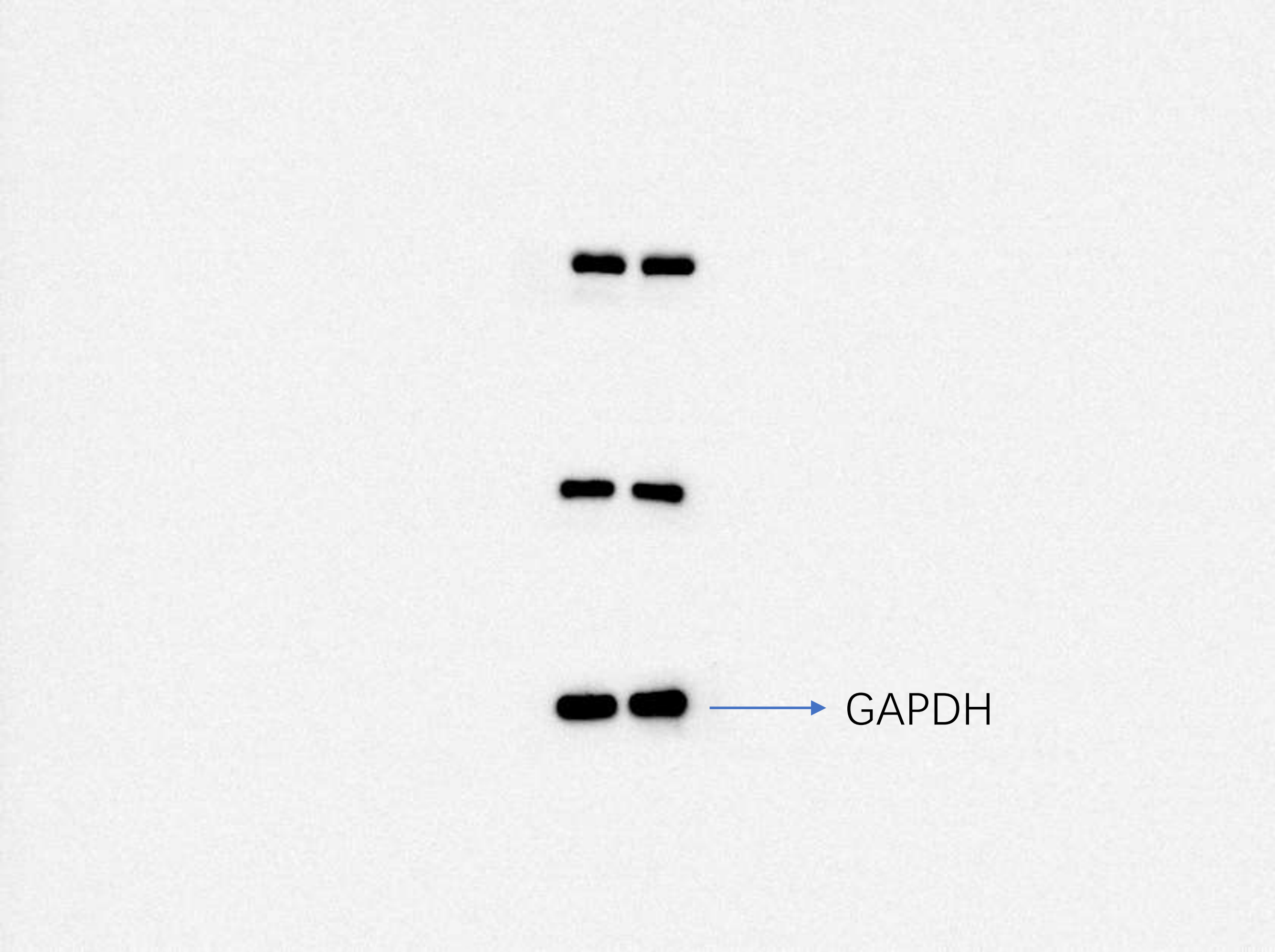

Result-5 (5G)

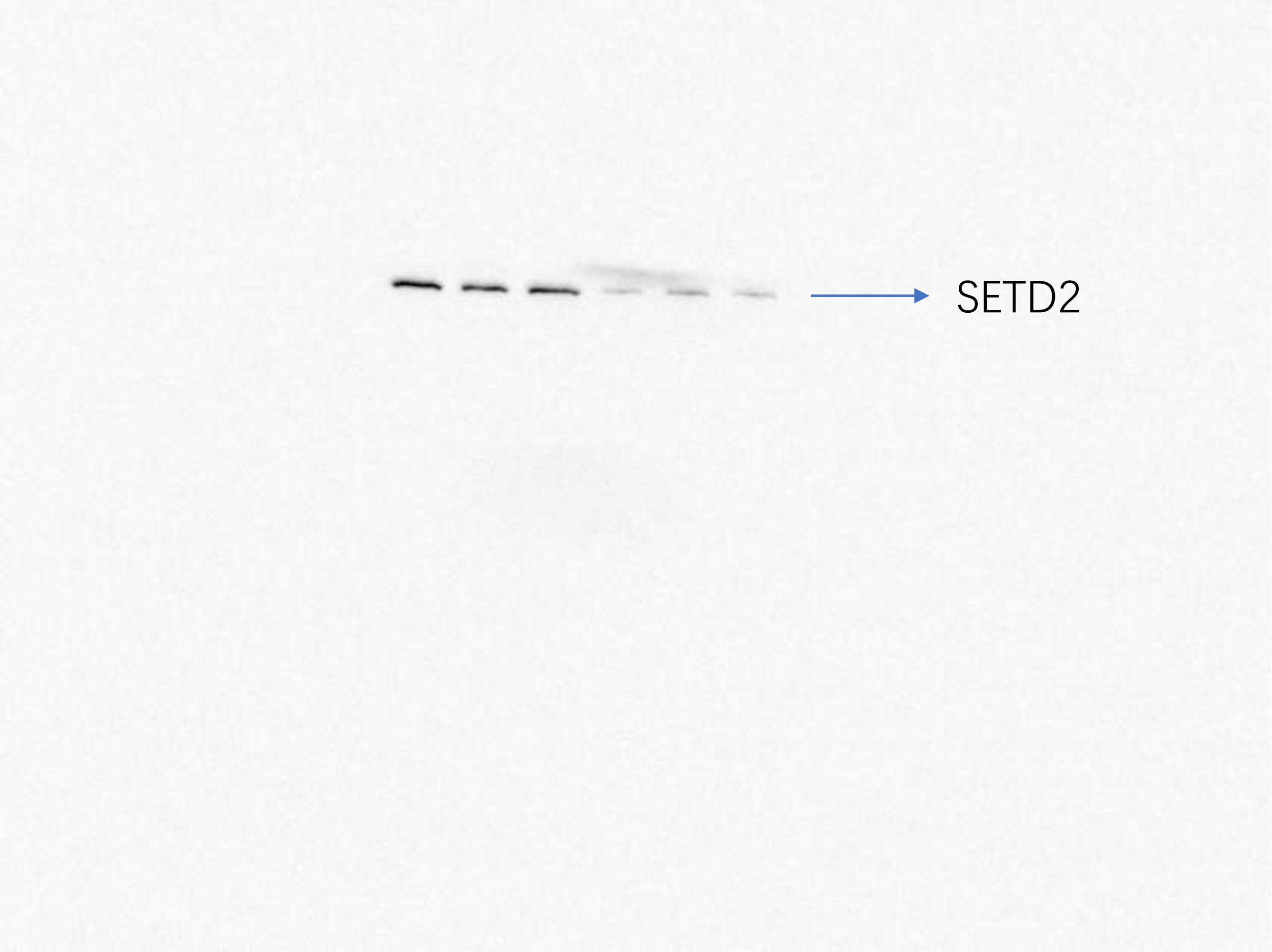

Result-5 (5G)

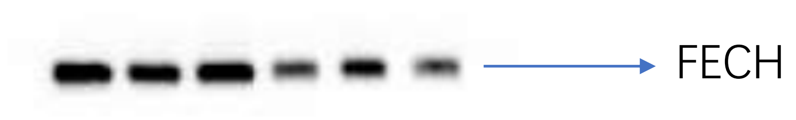

Result-5 (5G)

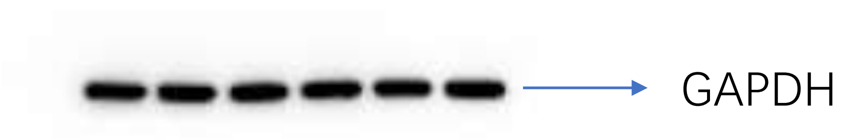

Result-6 (6B)

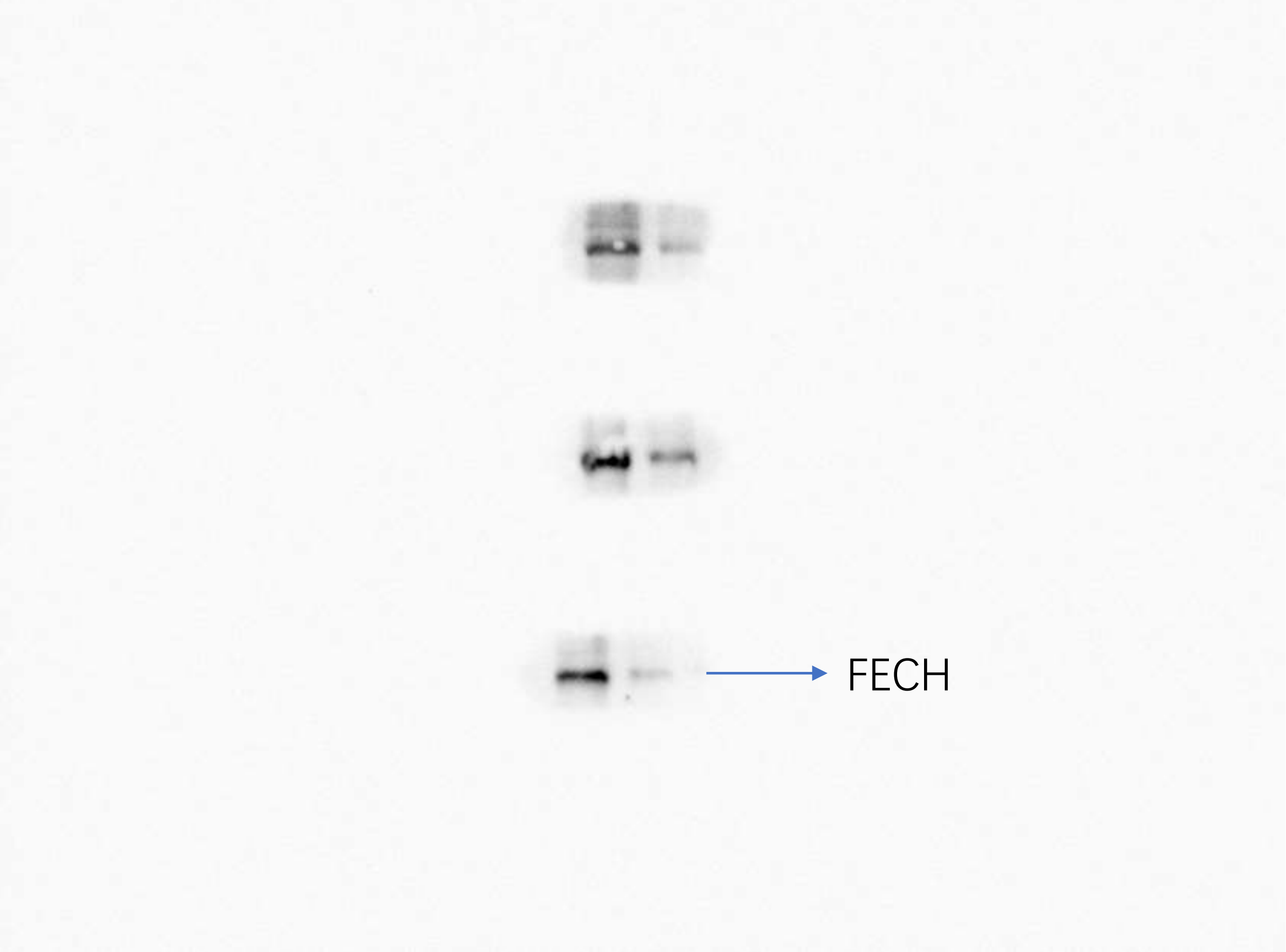

Result-6 (6B)

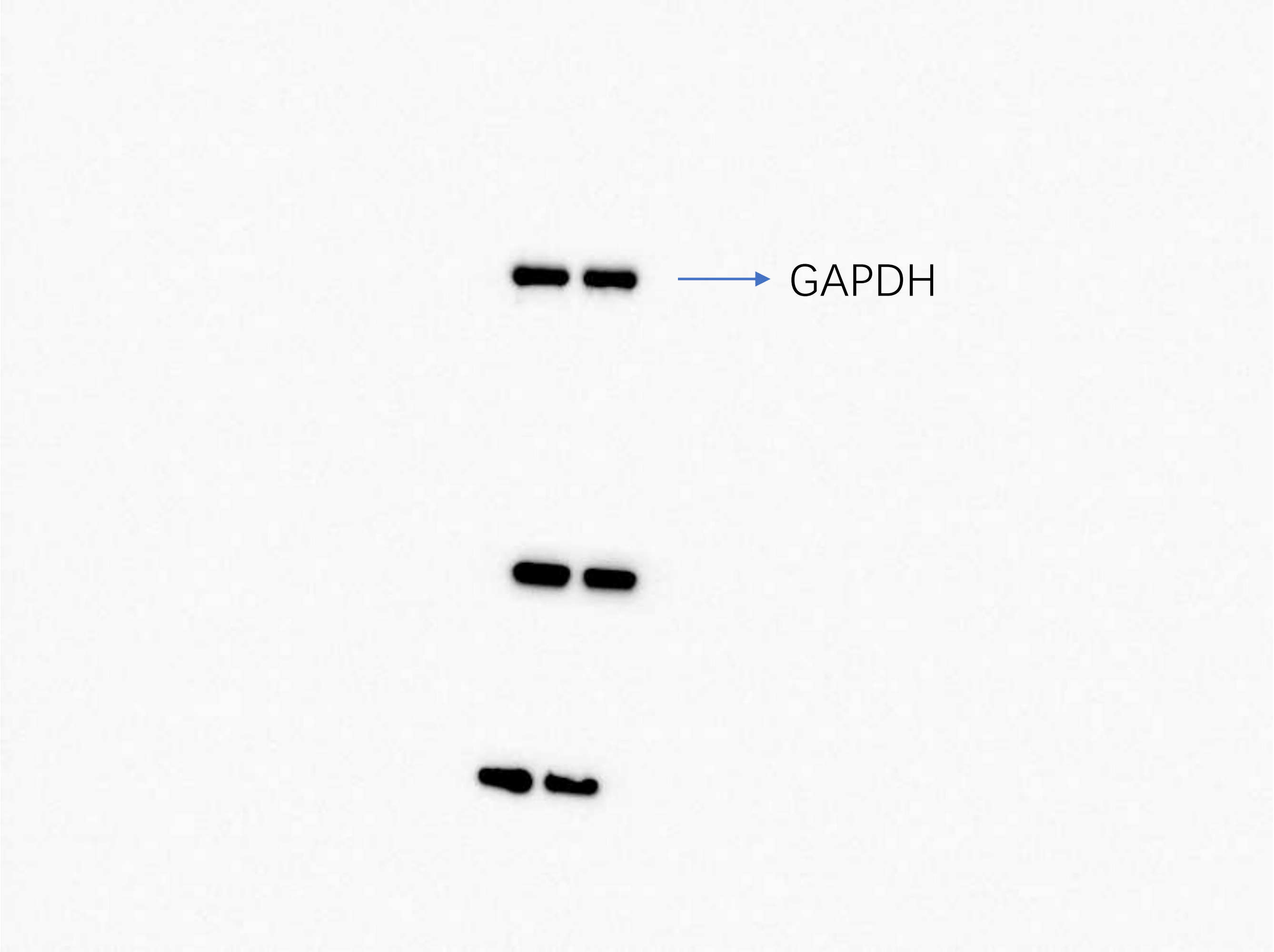

Result-6 (6B)

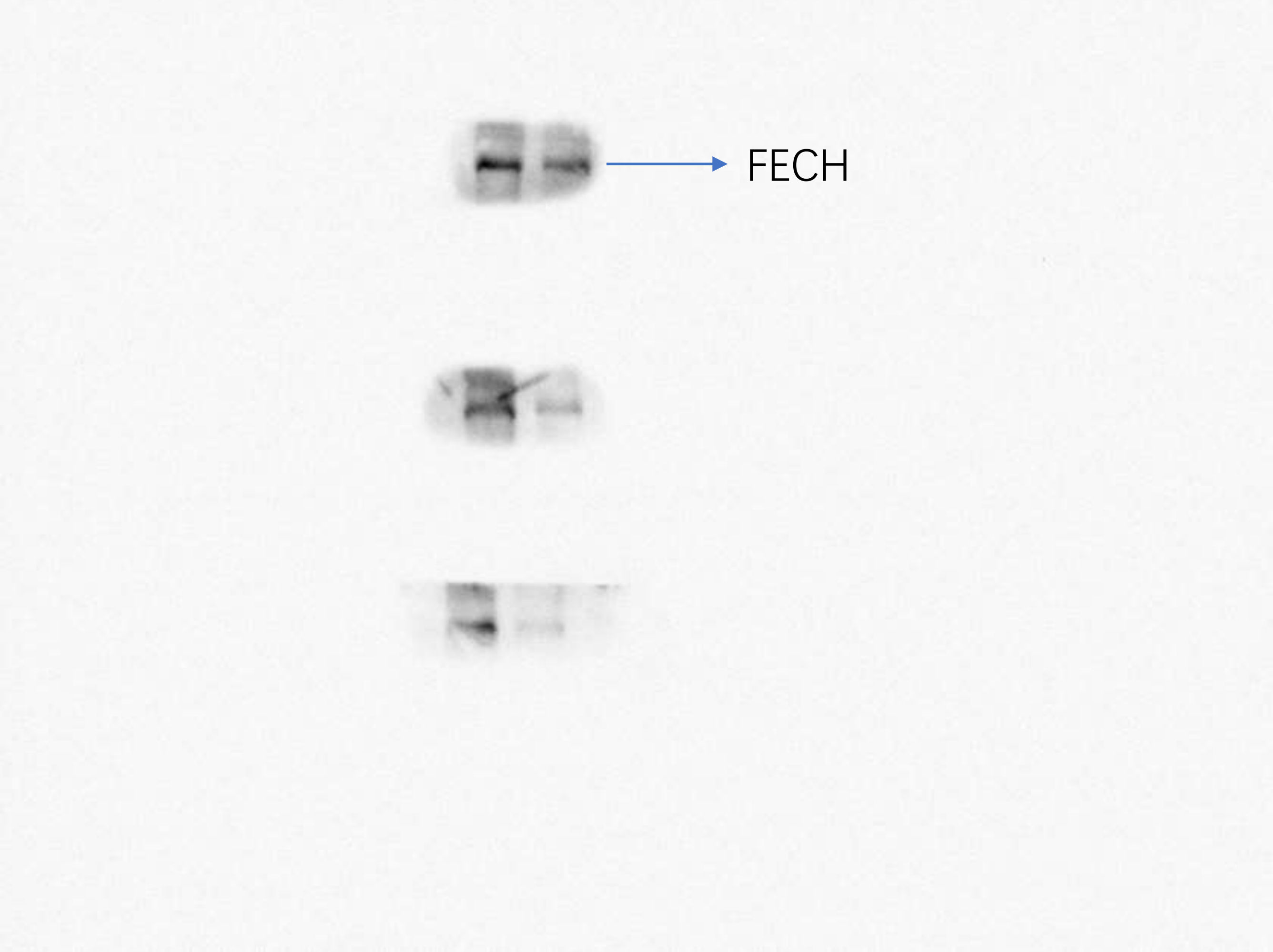

Result-6 (6B)

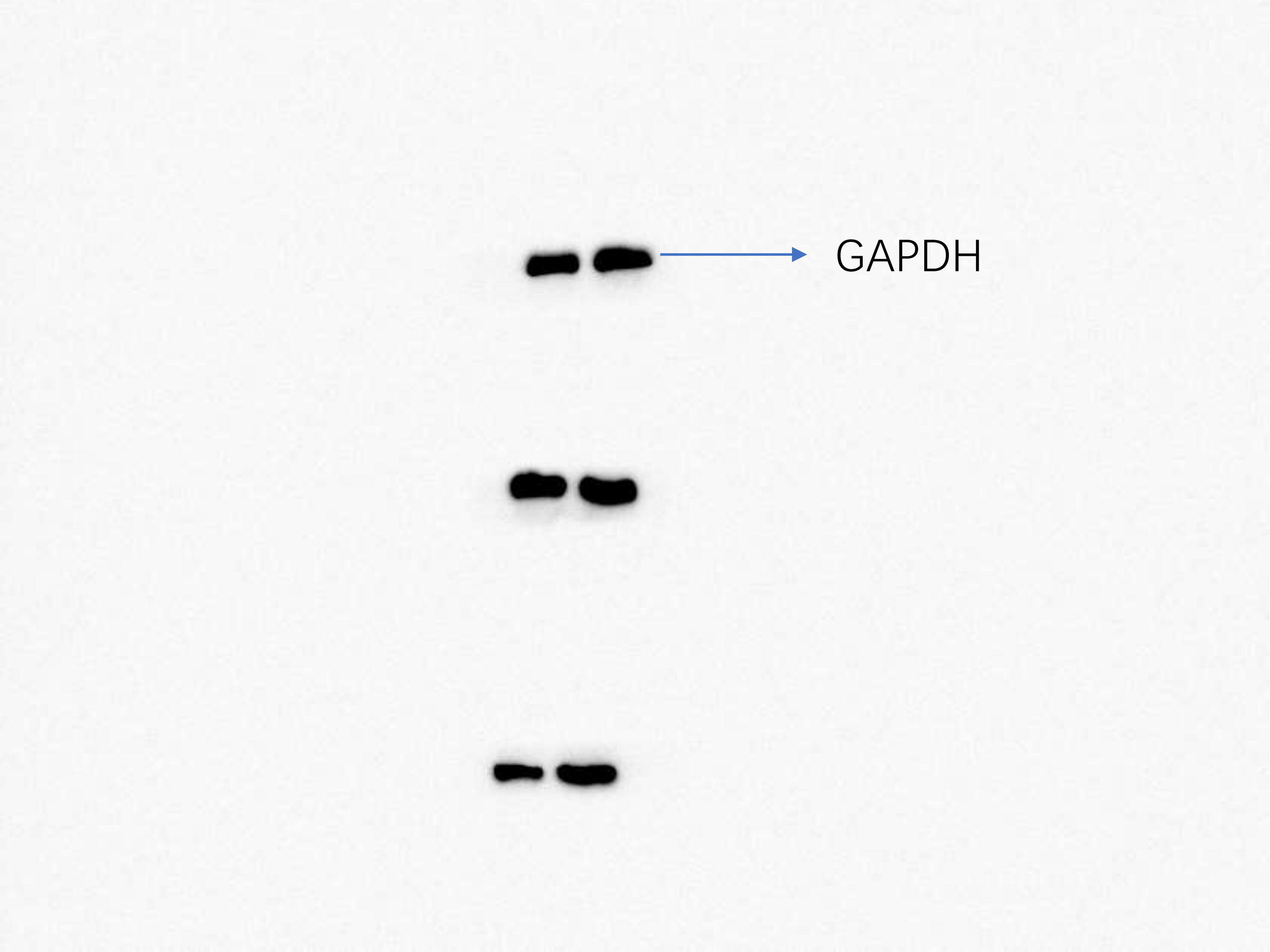

Result-6 (6I)

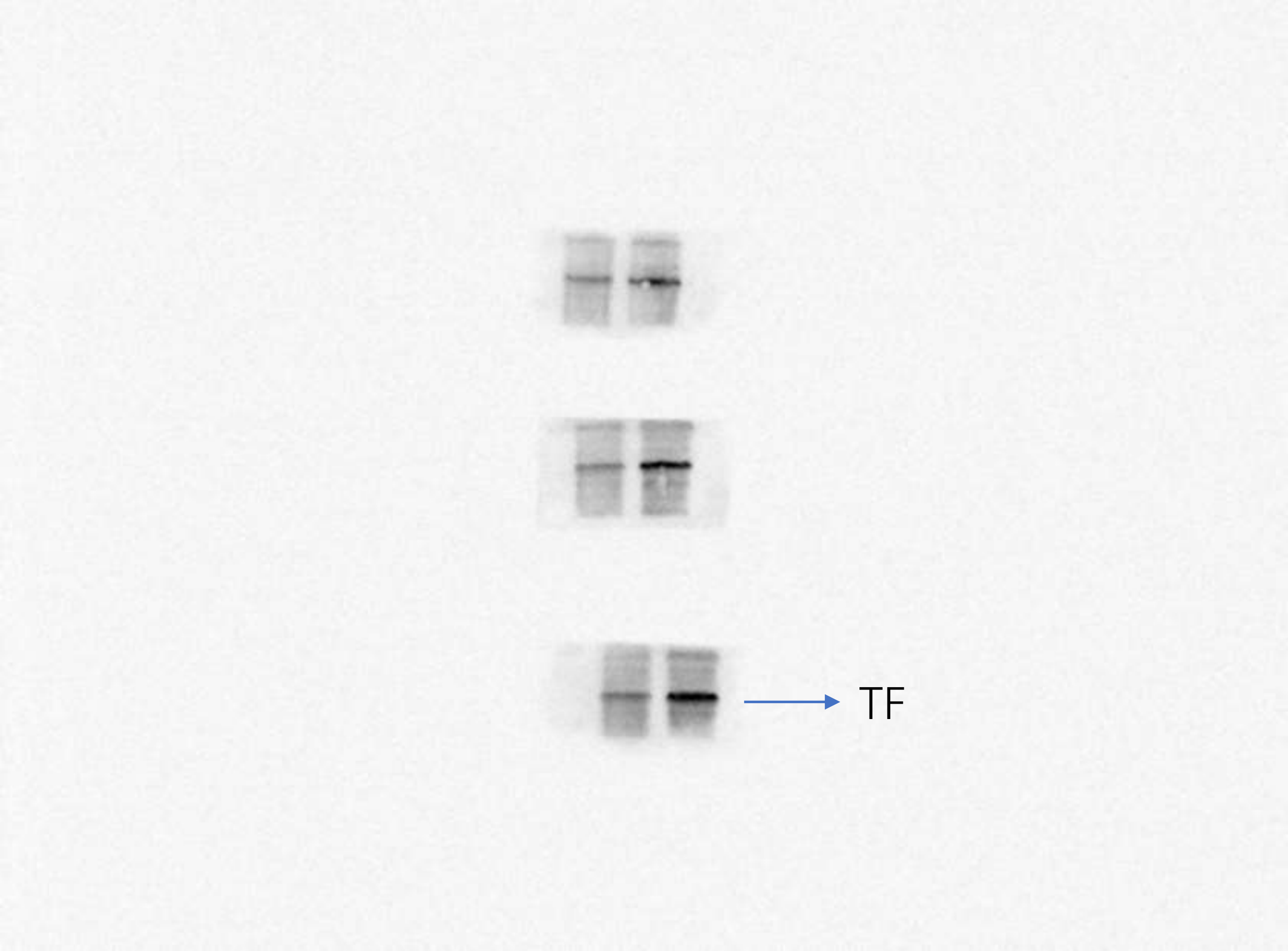

Result-6 (6I)

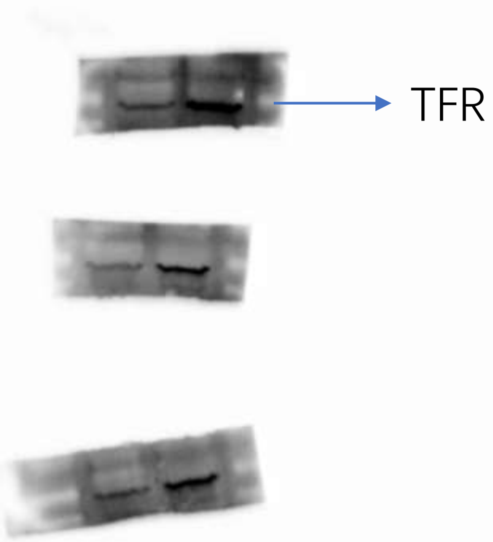

Result-6 (6I)

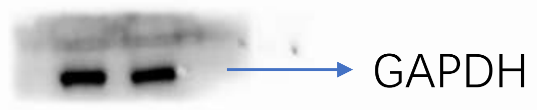

Result-6 (6I)

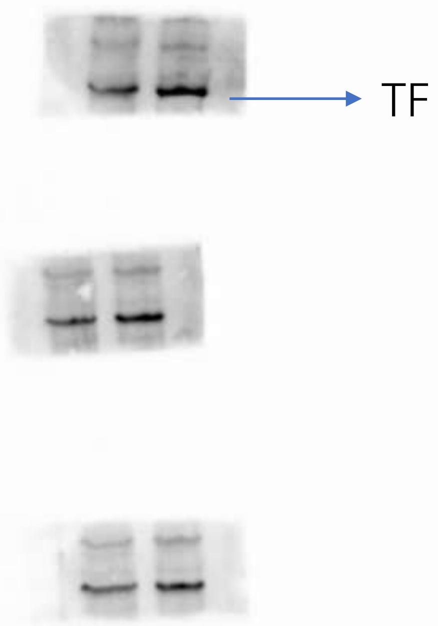

Result-6 (6I)

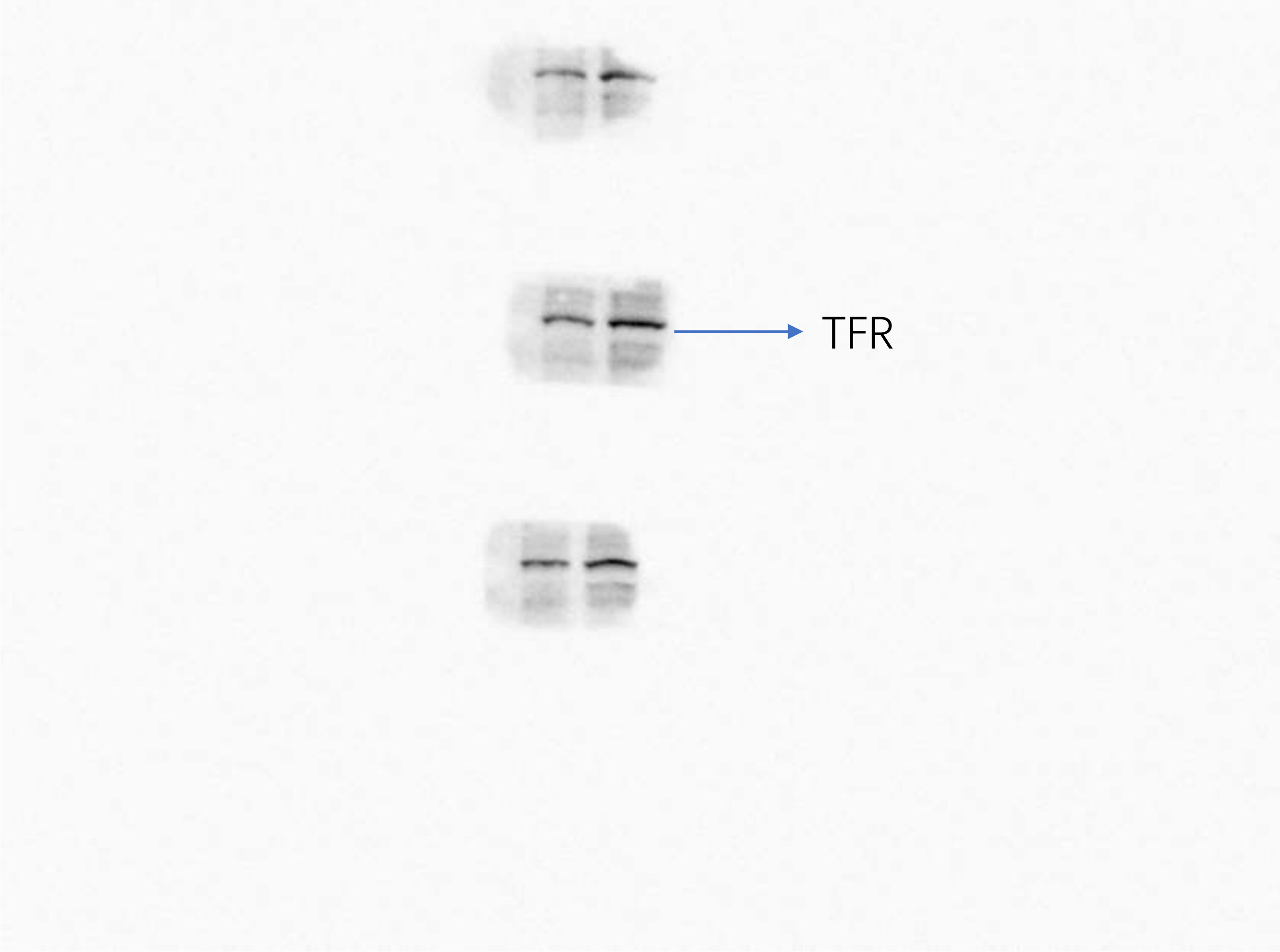

Result-6 (6I)

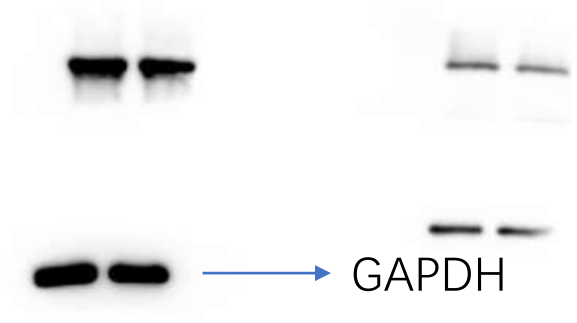

Result-7 (7F)

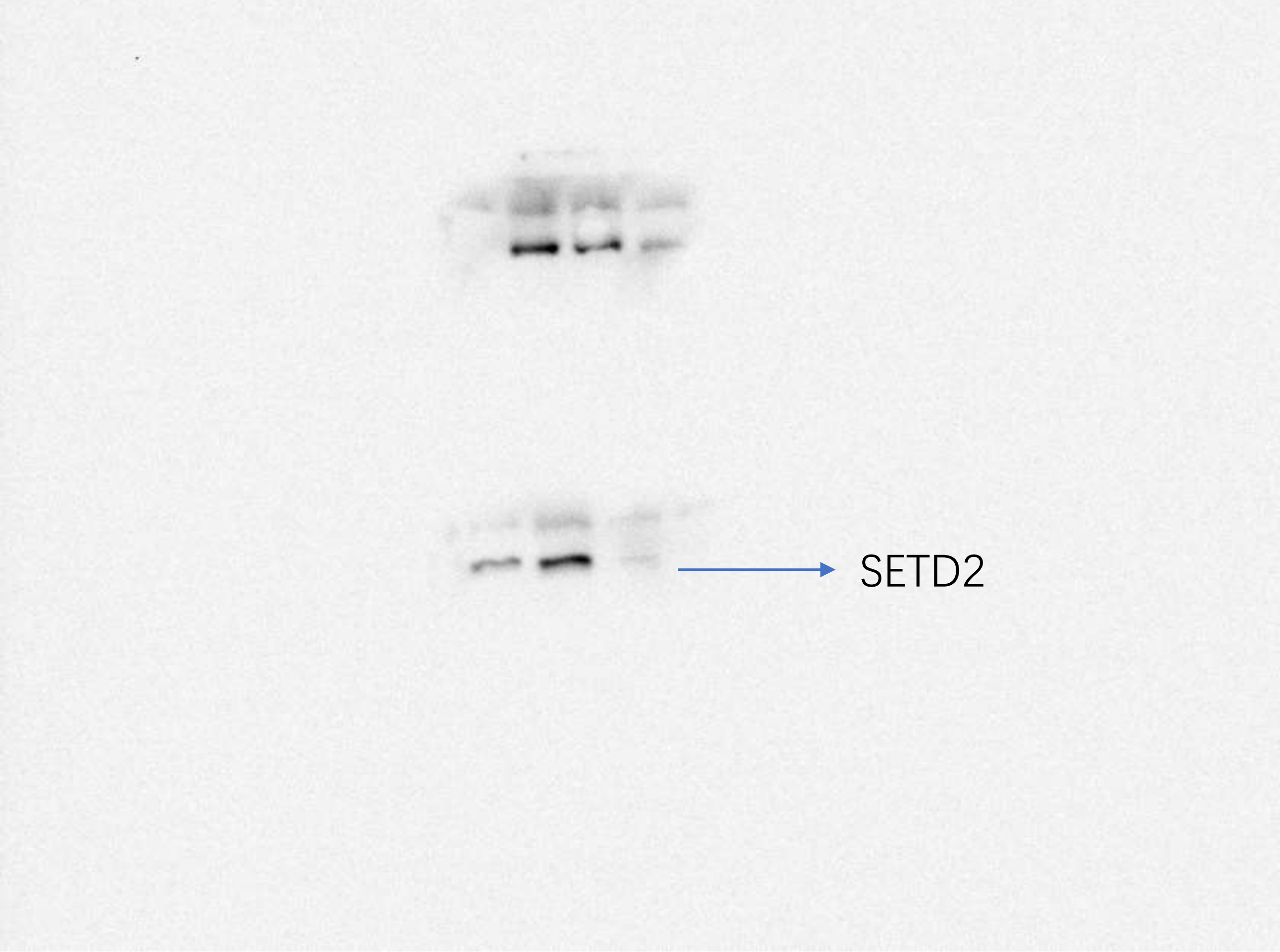

Result-7 (7F)

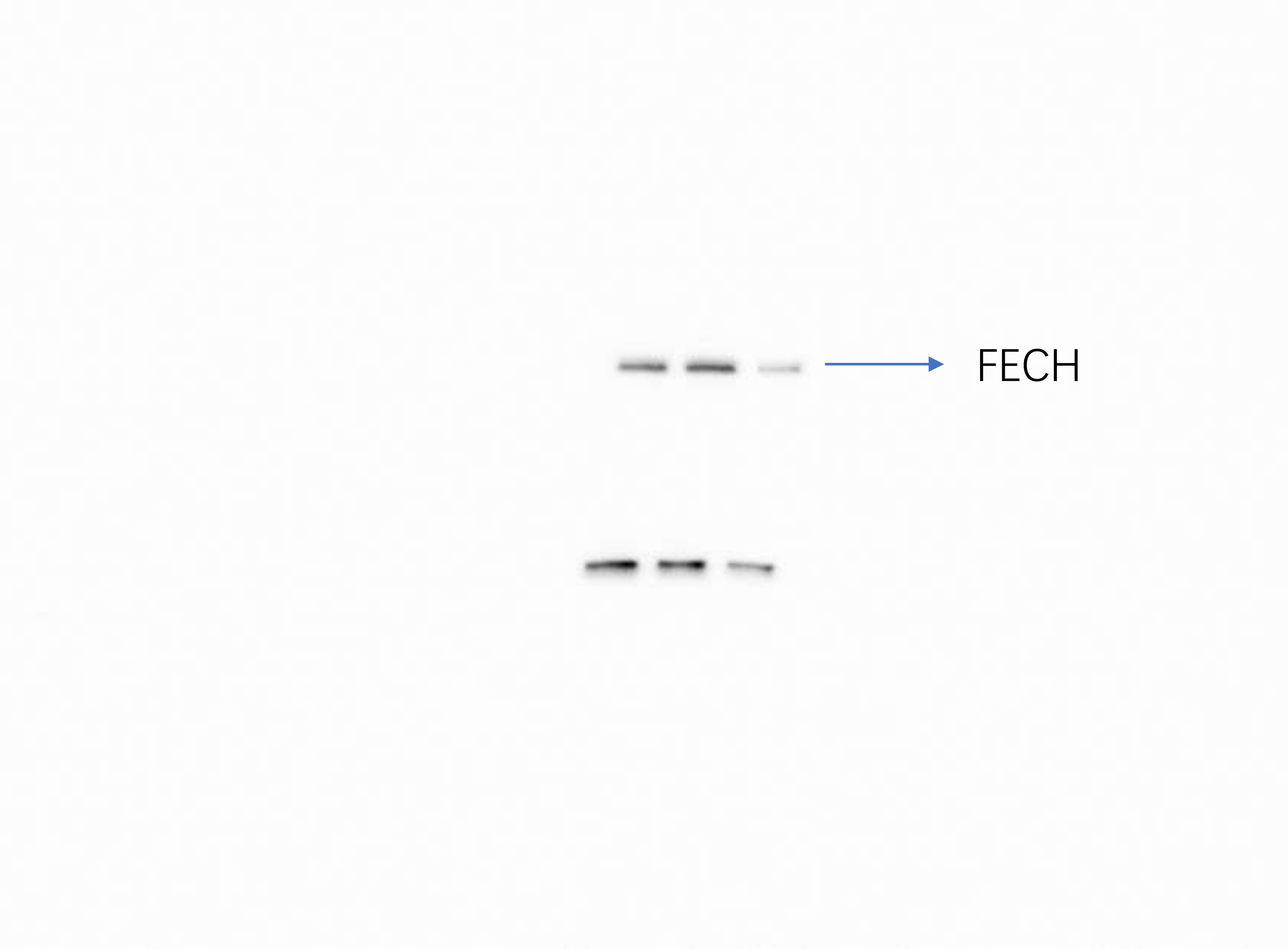

Result-7 (7F)

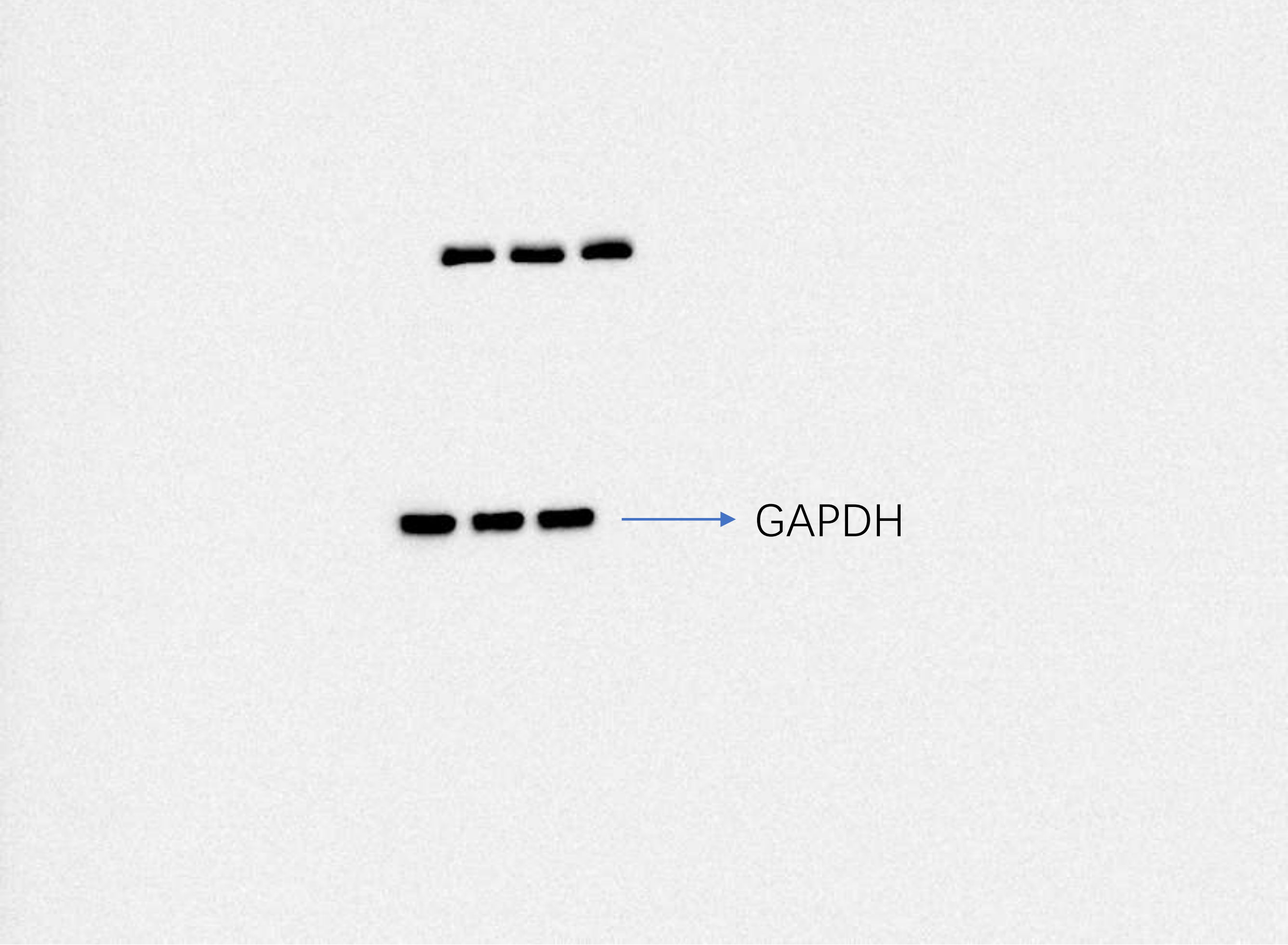

Supplement: Supplementary file 3 — Original Data File [file 41419_2023_6057_MOESM3_ESM.pdf]
